# ecpc: an R-package for generic co-data models for high-dimensional prediction

**DOI:** 10.1186/s12859-023-05289-x

**Published:** 2023-04-26

**Authors:** Mirrelijn M. van Nee, Lodewyk F. A. Wessels, Mark A. van de Wiel

**Affiliations:** 1 Epidemiology & Data Science, Amsterdam Public Health research institute, Amsterdam University Medical Centers, Amsterdam, The Netherlands; 2 Molecular Carcinogenesis, Netherlands Cancer Institute, Amsterdam, The Netherlands; 3 Computational Cancer Biology, Oncode Institute, Amsterdam, The Netherlands; 4 Intelligent Systems, Delft University Medical Centers, Delft, The Netherlands

**Keywords:** High-dimensional data, Penalised generalised linear models, Empirical Bayes, Prior information, **R**

## Abstract

**Background:**

High-dimensional prediction considers data with more variables than samples. Generic research goals are to find the best predictor or to select variables. Results may be improved by exploiting prior information in the form of co-data, providing complementary data not on the samples, but on the variables. We consider adaptive ridge penalised generalised linear and Cox models, in which the variable-specific ridge penalties are adapted to the co-data to give a priori more weight to more important variables. The **R**-package **ecpc** originally accommodated various and possibly multiple co-data sources, including categorical co-data, i.e. groups of variables, and continuous co-data. Continuous co-data, however, were handled by adaptive discretisation, potentially inefficiently modelling and losing information. As continuous co-data such as external *p* values or correlations often arise in practice, more generic co-data models are needed.

**Results:**

Here, we present an extension to the method and software for generic co-data models, particularly for continuous co-data. At the basis lies a classical linear regression model, regressing prior variance weights on the co-data. Co-data variables are then estimated with empirical Bayes moment estimation. After placing the estimation procedure in the classical regression framework, extension to generalised additive and shape constrained co-data models is straightforward. Besides, we show how ridge penalties may be transformed to elastic net penalties. In simulation studies we first compare various co-data models for continuous co-data from the extension to the original method. Secondly, we compare variable selection performance to other variable selection methods. The extension is faster than the original method and shows improved prediction and variable selection performance for non-linear co-data relations. Moreover, we demonstrate use of the package in several genomics examples throughout the paper.

**Conclusions:**

The **R**-package **ecpc** accommodates linear, generalised additive and shape constrained additive co-data models for the purpose of improved high-dimensional prediction and variable selection. The extended version of the package as presented here (version number 3.1.1 and higher) is available on (https://cran.r-project.org/web/packages/ecpc/).

**Supplementary Information:**

The online version contains supplementary material available at 10.1186/s12859-023-05289-x.

## Background

Generalised linear models (GLMs) [1] are the cornerstone of many statistical models for prediction and variable selection purposes, modelling the relation between outcome data and observed data. When observed data are high-dimensional, with the number of variables far exceeding the number of samples, these models may be penalised to account for the high-dimensionality. Well known examples include the ridge [2], lasso [3] and elastic net penalty [4]. One of the main assumptions underlying generalised linear models is that all variables are exchangeable. In many high-dimensional settings, however, this assumption is questionable [5]. For example, in cancer genomics, variables may be grouped according to some biological function. Variables within these groups may have a similar effect, while variables from different groups have a different effect. Hence, variables are exchangeable within groups, but not between groups. To alleviate the exchangeability assumption, shared information may be modelled explicitly in the prior distribution of the variables, e.g. by introducing shared group penalties, penalising variables in a group similarly and penalising more important groups of variables relatively less (as done by [6]). The shared prior information may be represented in data matrices, called co-data, to distinguish the main, observed data with information on the samples from the complementary data with information on the variables. In genomics, for example, the co-data matrix columns may contain *p* values representing the strength of association between each variable and outcome from external studies, correlations between mRNA and DNA, dummy variables for chromosomes and pathway information. When the co-data are related to the effect sizes of variables, these data may be exploited to improve prediction and variable selection in high-dimensional data settings.

Various **R**-packages accommodate approaches to incorporate some form of co-data. Early methods such as **grplasso** [7] and **gglasso** [8] allow for categorical, or grouped, co-data, by using group lasso penalties. As these penalties are governed by one overall penalty parameter, these types of penalties may be not flexible enough to model the relation between the effect sizes and grouped co-data. To increase this flexibility, other methods were developed that estimate multiple, group-specific penalty (or prior) parameters, using efficient empirical Bayes approaches. Examples include **GRridge** [6] for group-adaptive ridge penalties (normal priors), **graper** [9] for group-adaptive spike-and-slab priors and **gren** [10] for group-adaptive elastic net priors. Our method **ecpc** [11] presents a flexible empirical Bayes approach to extend the use of grouped co-data to various other (and potentially multiple) co-data types, such as hierarchical groups (e.g. gene ontologies) and continuous co-data, for multi-group adaptive ridge penalties. For continuous co-data, however, the normal prior variances corresponding to the ridge penalties are not modelled as a function of the continuous co-data variable, but rather as a function of groups of variables corresponding to the adaptively discretised co-data variable. When the relation between the prior variance and continuous co-data is non-constant and/or “simple”, e.g. linear, the adaptive discretisation may lead to a loss of information and/or inefficiently model the relation. The package **fwelnet** [12] develops feature-weighted elastic net for continuous co-data specifically (there called “features of features”). Regression coefficients are estimated jointly with co-data variable weights, modelling the variable-specific elastic net penalties by a normalised, exponential function of the co-data. For categorical co-data, **fwelnet** boils down to an elastic net penalty on the group level [12], governed by one overall penalty parameter. Hence, it may lack flexibility when compared to empirical Bayes methods estimating multiple penalties. The package **squeezy** [13] presents fast approximate marginal likelihood estimates for group-adaptive elastic net penalties, but is available for grouped co-data only.

Here, we present an extension of the **R**-package **ecpc** to generic co-data models, in particular for continuous co-data such as external *p* values. First, we show how a classical linear regression model may be used to regress the (unknown) variable-specific normal prior variances on the co-data. This provides a flexible parsimonious framework to obtain feature-specific penalties. The co-data variable weights are estimated with an empirical Bayes moment estimator, slightly modified from [11]. Then, we present how the estimation procedure may be extended straightforwardly to model the relation between the prior variances and co-data by generalised additive models [14] for modelling non-linear functions and by shape constrained additive models [15], e.g. for positive and monotonically increasing functions. This extension benefits the stability and interpretation of the estimated relation between co-data and the prior variances, especially when a basic linear model does not represent this relation well. Besides, we use ideas from [13] to transform the adaptive ridge penalties to elastic net penalties using the package **squeezy**. Either this approach or the previously implemented posterior selection approaches [11] may be used for variable selection.

### Contributions

The empirical Bayes estimation method [11] is extended to the continuous case. The main contributions of this software paper, newly extending the existing **R**-package **ecpc**, are as follows:

- Co-data are provided to the main function ecpc() in the more generic format of a co-data matrix (input argument Z), instead of a list of group sets (input argument groupsets). Besides dummy variables for group membership information, a co-data matrix may contain continuous co-data.
- The empirical Bayes estimates may be additionally penalised with a generalised ridge penalty (input argument paraPen, similar to the **R**-package **mgcv**) and/or subjected to constraints (input argument paraCon). This may be used to model the prior variances as non-linear and/or shape-constrained function of the co-data.
- The adaptive ridge penalty estimates given by ecpc() may be transformed with squeezy() to elastic net penalties to obtain sparse regression coefficient estimates.

## Implementation

**Fig. 1:**
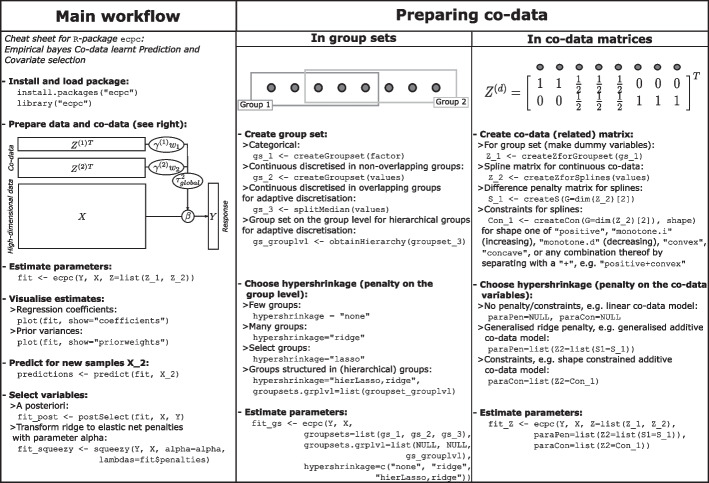
Cheat sheet for the main functions and work flow of the **R**-package **ecpc**, available as pdf-file on https://github.com/Mirrelijn/ecpc

The main function in the **R**-package is the eponymous function ecpc(), which fits a ridge penalised generalised linear model by estimating the co-data variable weights and regression coefficients subsequently. The function outputs an object of the S3-class ‘ecpc’, for which the methods summary(), print(), plot(), predict() and coef() have been implemented. See the index in ?“ecpc-package” for a list of all functions, including functions for preparing and visualising co-data, or see Fig. 1 for a cheat sheet of the main functions and workflow of the package.

### Data input

The function ecpc() considers the following data. The response data $\varvec{Y}\in {\mathbb {R}}^n$ are given in input argument Y. The observed high-dimensional data $X\in {\mathbb {R}}^{n\times p}$ with $p\gg n$, which contain information on the *n* samples of $\varvec{Y}$, are given in X. The co-data of possibly multiple co-data matrices $Z^{(d)}\in {\mathbb {R}}^{p\times G_d}$, $d=1,\ldots ,D$, which contain prior information on the *p* variables of *X*, are given in Z. Generally, co-data matrices may include continuous or categorical co-data. For categorical co-data, dummy variables should be provided. For categorical co-data with overlapping categories, dummy variables may be weighted accordingly to account for multiplicity (see [11]). The helper function createZforGroupset() may be used to create a co-data matrix from a list of (overlapping) groups. Co-data should not contain missing values. When the missingness is deemed uninformative, existing methods may be used to impute the missing values, e.g. by the co-data variable mean. When the missingness is suspected to be informative, missing values should be set to 0 and an extra categorical co-data variable (1 for missing 0 else) should be included.

### Response model and co-data model

**Fig. 2:**
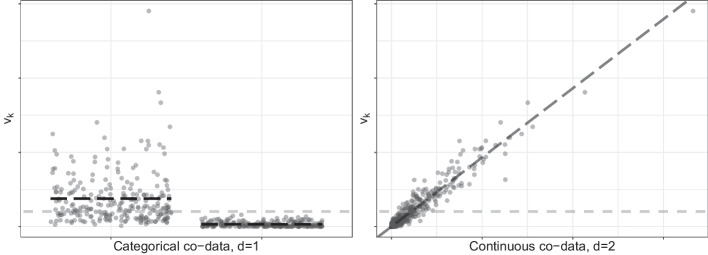
Illustration of the linear co-data model. Given the true effect sizes $\beta _k^2$ (points), the prior parameters may be interpreted as follows: (i) the scaling factor $\tau ^2_{global}$ (grey dashed line) quantifies the overall expected effect size, which is independent of the co-data; (ii) each scaled co-data variable weight $\tau ^2_{global}\gamma ^{(d)}_g$ (black dashed lines) quantifies the expected effect size in a group for categorical co-data or the expected increase in effect size for one unit increase in continuous co-data, i.e. the slope of the line; (iii) the co-data weights $w_d$, $d=1,2$, then quantify importance of multiple co-data sets. Note that in practice, the true effect sizes are unknown and estimation of the prior parameters is done by the empirical Bayes approach described below

Currently, ecpc() allows for a linear, logistic and Cox survival model (input argument model). Generally, the response is modelled with a generalised linear (or Cox) model with canonical link function $g(\cdot )$, parameterised with regression coefficients $\varvec{\beta }\in {\mathbb {R}}^p$. Furthermore, the regression coefficients follow a normal prior, with variance $v_k, k=1,\ldots ,p,$ inversely proportional to the variable-specific ridge penalty, in which the prior variance is regressed on the co-data. First, consider the linear co-data model in which the prior variance is modelled as a linear function of the co-data:

1 $$\begin{aligned} &Y_{i} | \varvec{X}_{i},\varvec{\beta } \overset{ind.}{\sim }\ \pi \left( Y_{i} | \varvec{X}_{i},\varvec{\beta }\right) ,\ E_{Y_{i}|\varvec{X}_{i},\varvec{\beta }}(Y_{i})=g^{-1}(\varvec{X}_{i}\varvec{\beta }),\ i=1,\ldots ,n,\\&\quad \beta _{k} \overset{ind.}{\sim }\ N(0,v_k),\ v_{k}=\tau _{global}^2\sum _{d=1}^D w_{d} \varvec{Z}_k^{(d)} \varvec{\gamma }^{(d)},\ k=1,\ldots ,p, \end{aligned}$$

with $\varvec{X}_i$ and $\varvec{Z}_k$ the $i^{th}$ and $k^{th}$ row of *X* and *Z* respectively, $\varvec{\gamma }^{(d)}\in {\mathbb {R}}^G$ the co-data variable weights for co-data matrix *d*, $\varvec{w}$ the co-data matrix weights and $\tau _{global}^2$ a scaling factor which may improve numerical computations in practice. The linear co-data model and interpretation of the prior parameters are illustrated in Fig. 2. When the data *X* consist of multiple data modalities, like gene expression data, copy number data and methylation data in genomics, scaling factors specific to the data modalities may be used [16, 17] and estimated with **ecpc**.

#### Co-data models

**Fig. 3:**
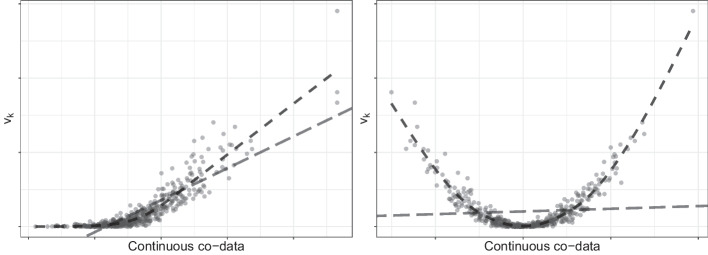
Illustration of the non-linear co-data models. Given the true effect sizes $\beta _k^2$ (points), a linear co-data model may capture the relation insufficiently, making estimation of non-linear relations desirable. A generalised additive model estimates a smooth non-linear relation. Additionally, one may further impose constraints, e.g. monotonicity (left) or convexity (right), in a shape-constrained additive model

The extension of **ecpc** implements three types of co-data models, based on a linear model, generalised additive model [14] and shape-constrained additive model [15]. The flexibility of the additive models is important in case the relation between the co-data variables and effect sizes is non-linear. A linear co-data model then inadequately exploits the co-data, while additive models are able to adapt to the underlying relation, as illustrated in Fig. 3. For illustration, consider one co-data source with *G* co-data variables and set the scaling parameter $\tau _{global}^2$ to 1:

$$\begin{aligned} \varvec{v}&= \sum _{g=1}^G \varvec{Z}_g\gamma _g&{\text {(linear co-data model)}}\\ \varvec{v}&= \sum _{g=1}^G s_g(\varvec{Z}_g)&{\text {(generalised additive co-data model)}}\\ \varvec{v}&= \sum _{g=1}^G c_g(\varvec{Z}_g)&{\text {(shape-constrained additive co-data model)}} \end{aligned}$$

with $Z_g$ extended to continuous co-data, $s_g$ a smooth function and $c_g$ some shape-constrained function, e.g. monotone or convex, both applied element-wise. Generally, the larger the variable-specific prior variance, the smaller the corresponding ridge penalty and the larger the a priori expected variable effect size.

In practice, the smooth and shape-constrained functions are estimated by using a basis expansion to recast the problem into a (constrained) linear model (as originally proposed by, for example, [18]). So, for a basis expansion consisting of $J_g$ basis functions $\phi _{g,j}(\cdot )$, $j=1,\ldots ,J_g$, for co-data variable $\varvec{Z}_g$:

$$\begin{aligned} s_g(\varvec{Z}_g)&= \sum _{j=1}^{J_g} \phi _{g,j}(\varvec{Z}_g)\gamma _{g,j} = \Phi _g\varvec{\gamma }_g,\ \qquad \varvec{v} = \sum _{g=1}^G \Phi _g\varvec{\gamma }_g, \end{aligned}$$

with $\Phi _g\in {\mathbb {R}}^{p\times J_g}$ the matrix of co-data variable vector $\varvec{Z}_g\in {\mathbb {R}}^p$ evaluated in all $J_g$ basis functions. Any basis expansion may be used by supplying the corresponding basis expansion matrix $\Phi _g$ as co-data in input argument Z in ecpc().

#### Choice of basis expansion

The type and number of basis functions should in general be chosen such that they are flexible enough to approximate the underlying function well. To avoid overfitting for too many basis functions, the coefficients may be estimated by optimising the likelihood penalised by a smoothing penalty. While our software allows the user to supply any basis expansion, we focus here on the popular p-splines (see [19] for an introduction). This approach combines flexible spline basis functions with a quadratic smoothing penalty on the differences of the spline coefficients (difference penalty matrix $S_g$ in Equation (5)). The level of smoothness is then automatically tuned by estimation of the smoothing penalty ($\lambda _g$ in Eq. (5)). For shape-constrained functions, we consider a p-spline basis expansion and constrain the spline coefficients [15].

The helper function createZforsplines() may be used to create the p-splines expansion matrix $\Phi _g$ corresponding to co-data variable $\varvec{Z}_g$ for input argument Z. The function createS() may be used to create the corresponding difference penalty matrix $S_g$ for input argument paraPen. The function createCon() may be used to create constraints for functions that are positive, monotonically increasing or decreasing, convex or concave, or any combination thereof.

### Model parameters estimation

Prior parameters and regression coefficients are estimated with an empirical Bayes approach, following [11]. In short, first the global scaling parameter $\tau ^2_{global}$ is estimated, then the co-data variable weights $\varvec{\gamma }^{(d)}$ for each co-data matrix *d* separately and then the co-data weights $\varvec{w}$. To ensure stability and identifiability of the estimates when co-data variables or sources are (increasingly) correlated, the co-data variable weights $\varvec{\gamma }^{(d)}$ may be penalised (e.g. see Eq. (5)) and the co-data source weights are estimated subject to the constraint $\varvec{w}\ge 0$. After, given the prior parameter estimates, the regression coefficients $\varvec{\beta }$ are estimated by maximising the penalised likelihood (equivalent to maximising the posterior).

Here, the empirical Bayes estimation for the co-data variable weights $\varvec{\gamma }^{(d)}$ [11] is extended for continuous co-data. The co-data variable weights are estimated with moment estimation by equating theoretical moments to empirical moments. For co-data that represent groups of variables [11], the empirical moments are averaged over all variables in that group, leading to a linear system of *G* equations and *G* unknowns. For continuous co-data, we simply form one group per variable, leading to the following linear system of *p* equations and *G* unknowns:

2 $$\begin{aligned}&(C\circ C)Z\varvec{\gamma }=\varvec{b}, \end{aligned}$$

with $\circ$ representing the Hadamard (element-wise) product. $C\in {\mathbb {R}}^{p\times p}$ and $\varvec{b}\in {\mathbb {R}}^{p}$ are derived in [11] and given by:

$$\begin{aligned} C&=(X^TWX+{\tilde{\Omega }})^{-1}X^TWX,\qquad \varvec{b}=\tilde{\varvec{\beta }}.^2-\tilde{\varvec{v}},\\ \tilde{\varvec{v}}&=\textrm{diag}((X^TWX+{\tilde{\Omega }})^{-1}X^TWX(X^TWX+{\tilde{\Omega }})^{-1}), \end{aligned}$$

with $\tilde{\varvec{\beta }}$ the maximum penalised likelihood estimate given an initial ${\tilde{\tau }}^2_{global}$ and corresponding constant diagonal ridge penalty matrix ${\tilde{\Omega }}$, with *W* a diagonal weight matrix used in the iterative weighted least squares algorithm to fit $\tilde{\varvec{\beta }}$, and with $\tilde{\varvec{v}}$ an estimate for the variance of $\tilde{\varvec{\beta }}$ with respect to the response data *Y*.

Note that storing the matrix *C* is memory-costly as it is a $p\times p$-dimensional matrix with *p* potentially tens of thousands of variables. *C* can, however, be written as matrix product of two smaller matrices $C=LR$ with $L\in {\mathbb {R}}^{p\times n}$ and $R\in {\mathbb {R}}^{n\times p}$. Instead of computing and storing *C* in one go, we compute it per block of *b* rows to alleviate memory costs: for each block of rows $C_{block}$ we only need to store the elements of $(C_{block}\circ C_{block})Z\in {\mathbb {R}}^{b\times G}$.

The main estimating equation boils down to solving a linear system, which is solved as is for linear co-data models, penalised with a generalised ridge penalty for generalised additive models or solved under constraints plus possibly penalised with a generalised ridge penalty for shape-constrained additive co-data models. The penalisation on the level of prior parameters ensures stable estimation of the co-data weights.

#### Linear co-data model

As the prior variance has to be positive, the resulting prior variance estimate is truncated at 0 after solving the linear system from (2):

3 $$\begin{aligned} \hat{\varvec{\gamma }}&= \underset{\varvec{\gamma }}{\textrm{argmin}}\ ||(C\circ C)Z\varvec{\gamma }-\varvec{b}||^2_2, \qquad \hat{\varvec{v}}=(Z\hat{\varvec{\gamma }})_+. \end{aligned}$$

In generalised linear models it is common to use a log-link for the response to enforce positivity, resulting in positive, multiplicative effects. Note that here, however, Equation (2) is the result of equating theoretical to empirical moments. Replacing $\varvec{b}$ by $\log (\varvec{b})$ would violate the moment equalities. Also, if we would enforce positivity instead by, for example, substituting $Z\varvec{\gamma }$ directly by $\varvec{v}=\exp (Z\varvec{\gamma }')$, the moment equations would not be linear anymore in $\varvec{\gamma }$, nor multiplicative, e.g. as $(C\circ C)\exp (Z\varvec{\gamma })\ne \exp ((C\circ C)Z\varvec{\gamma })=\prod _{g=1}^G\exp ((C\circ C)\varvec{Z}_g\gamma _g)$, with $\varvec{Z}_g$ the $g^{th}$ co-data variable. Hence, the advantage of simply post-hoc truncating $Z\hat{\varvec{\gamma }}$ is that the system of equations in see Eq. (3) is easily solved. Alternatively, shape constrained co-data models may be used to enforce positivity, as explained further on.

#### Generalised additive co-data model

For estimating the generalised additive co-data model coefficients in a non-linear co-data model, the least squares estimate in Eq. (3) is extended by penalising the coefficients with a difference penalty matrix $S_g$ with smoothing penalty parameter $\lambda _g$.

4 $$\begin{aligned} \hat{\varvec{\gamma }}_{GAM}&= \underset{\varvec{\gamma }}{\textrm{argmin}}\ \left\{ ||(C\circ C)Z_{GAM}\varvec{\gamma }-\varvec{b}||^2_2 + \sum _{g=1}^G \lambda _g \varvec{\gamma }^TS_g\varvec{\gamma }\right\} ,\\ \hat{\varvec{v}}&=(Z_{GAM}\hat{\varvec{\gamma }}_{GAM})_+, \end{aligned}$$

with $Z_{GAM}=[\Phi _1,\ldots , \Phi _G]$ the matrix of spline basis expansions for all *G* co-data variables and $\varvec{\gamma }_{GAM}=(\varvec{\gamma }_{1}^T,\ldots ,\varvec{\gamma }_{G}^T)^T$ the vector of all spline coefficients. This least-squares equation is of a form also known as penalised signal regression [20] and can be solved by the function gam() (or bam() for big data) of the **R**-package **mgcv**, for example. This function also provides fast and stable estimation of the penalties $\lambda _g$ [21] for multiple co-data sources and possibly multiple penalty matrices per co-data source jointly. Alternatively, when only one smoothing penalty matrix is provided per co-data source, the smoothing penalty and spline coefficients may be estimated per co-data source separately by using random splits as proposed in [11]. Our software uses bam() to solve $\hat{\varvec{\gamma }}$ by default and allows for random splits when only one smoothing penalty matrix is provided.

#### Shape-constrained additive co-data model

Prior assumptions on the shape of the relation between the prior variance and co-data, such as monotonicity or convexity, may be imposed by constrained optimisation of spline coefficients [15]. The co-data weight estimate is given by subjecting the possible solution of Eq. (4) to (in)equality constraints given in matrix $M_{(in)eq,g}$ and vector $\varvec{b}_{(in)eq,g}$:

5 $$\begin{aligned} &\left\{ \begin{array}{l} \hat{\varvec{\gamma }}_{g} = \underset{\varvec{\gamma }}{\textrm{argmin}}\ \left\{ ||(C\circ C)\Phi _{g}{\varvec{\gamma }}-{\varvec{b}}||^2_2 + \lambda _{g} {\varvec{\gamma }}^{TS}_{g}{\varvec{\gamma }}\right\} \\ \qquad {\text {s.t.}}\ M_{ineq,g}\varvec{\gamma }\le {\varvec{b}}_{ineq,g},\ M_{eq,g}\varvec{\gamma }={\varvec{b}}_{eq,g} \end{array}\right. \end{aligned}$$

Several shapes may be imposed by choosing $M_{ineq}$ and $b_{ineq}$ accordingly [15]: (i) positivity may be imposed by constraining the spline coefficients to be positive; (ii) monotonically increasing (decreasing) may be imposed by constraining the first order differences $\gamma _{i+1}-\gamma _i$ to be positive (negative); (iii) convexity (concavity) may be imposed by constraining second order differences $\gamma _{i+2} - 2\gamma _{i+1} + \gamma _i$ to be positive (negative); (iv) any combination of the shapes i-iii may be imposed by combining the corresponding constraints.

In [15] shape-constrained p-splines are developed to handle difficulties in optimising multiple smoothing penalties due to discontinuous gradients. Their **R**-package **scam**, however, cannot be readily used for signal regression, which differs from regular regression in that the spline basis matrix is multiplied by the known matrix $(C\circ C)$. Moreover, the smoothing parameter estimates are estimated using a generalised cross-validation (GCV) criterion, which we show below to overfit in the unconstrained case. Therefore, we rely on the simple approach of directly constraining the spline coefficients as in Equation (5).

We use the approach proposed in [11] to estimate the smoothing penalties: first we estimate the smoothing penalties $\lambda _g$ separately for each co-data variable $\varvec{Z}_g$ using random splits of the data. As this optimisation is in one dimension only, we use Brent’s algorithm from the general purpose optimisation **R**-package **optim**, which should be sufficient to handle discontinuous gradients. Then we estimate the spline coefficients $\varvec{\gamma }_g$ for each co-data variable $\varvec{Z}_g$ and corresponding spline basis function matrix $\Phi _g$.

When at least one of the co-data models is shape-constrained, the software uses the random splits in combination with lsqlincon() from the **R**-package **pracma** for constrained optimisation.

### Variable selection

The normal prior in Eq. (1) corresponding to adaptive ridge penalties leads to dense, i.e. non-zero, estimates for the regression coefficients $\varvec{\beta }$. To obtain sparser solutions, the adaptive ridge penalties may be transformed to elastic net penalties by modifying results from [13], as detailed below. The ridge penalties resulting from the fit with ecpc() are transformed with squeezy() from the **R**-package **squeezy**, which also estimates the elastic net penalised regression coefficients using the **R**-package **glmnet**. Alternatively, **ecpc** may use posterior selection to select variables, as formerly proposed [11]. The two approaches differ in how the level of sparsity is tuned: the user may tune the number of variables for posterior selection or tune the elastic net sparsity parameter $\alpha \in [0,1]$ when **squeezy** is used.

#### Transforming ridge penalties to elastic net penalties

In the proposed model in Eq. (1), the regression coefficients follow a normal prior corresponding to a ridge penalty. Now, suppose that each $\beta _k$ independently follows some other prior distribution $\pi (\beta _k)$, parameterised by variable-specific prior parameter $\lambda _k$ and with prior mean 0 and finite variance $\textrm{Var}(\beta _k)=Z\varvec{\gamma }=h(\lambda _k)$ for some known monotonic variance function $h(\cdot )$:

6 $$\begin{aligned} \beta _k\overset{ind.}{\sim }\pi (\beta _k),\ E(\beta _k)=0,\ \textrm{Var}(\beta _k)=h(\lambda _k)=\varvec{Z}_k\varvec{\gamma }. \end{aligned}$$

As example we consider the elastic net prior, corresponding to the elastic net penalty, with variable-specific elastic net penalty. Recently, it was shown that when the prior parameters are group-specific, the marginal likelihood -as function of $\lambda _k$- is approximately the same as the marginal likelihood as function of normal prior parameters $\varvec{\gamma }$, as the prior distribution of the linear predictor $\varvec{\eta }=X\varvec{\beta }$ is asymptotically normally distributed [13]:

$$\begin{aligned} \pi (\varvec{Y}|X,\varvec{\lambda })\approx \pi (\varvec{Y}|X,\varvec{\gamma }) \end{aligned}$$

This result also holds for priors with variable-specific, finite variance [22]. We may use this result to obtain approximate method of moment equations for other priors.

Denote by $\hat{\varvec{\beta }}_R(Y)$ the ridge penalised maximum likelihood estimate as function of the observed response data $\varvec{Y}$. The method of moments equations are given by equating the theoretical marginal moments to the empirical moments [11]:

$$\begin{aligned} E_{\varvec{Y}|\varvec{\lambda }}({\hat{\beta }}_{k,R}^2(\varvec{Y})) = {\hat{\beta }}_{k,R}^2(\varvec{Y}),\ \text {for } k=1,\ldots ,p. \end{aligned}$$

Using the normal approximation for the marginal likelihood we obtain:

$$\begin{aligned} E_{\varvec{Y}|\varvec{\lambda }}\left( {\hat{\beta }}_{k,R}^2(\varvec{Y})\right)&= \int _{\varvec{Y}} {\hat{\beta }}_{k,R}^2(\varvec{Y}) \pi (\varvec{Y}|X,\varvec{\lambda }) \textrm{d}\varvec{Y}\\&\quad \approx \int _{\varvec{Y}}{\hat{\beta }}_{k,R}^2(\varvec{Y}) \pi (\varvec{Y}|X,\varvec{\gamma })\textrm{d}\varvec{Y} = E_{\varvec{Y}|\varvec{\gamma }}\left( {\hat{\beta }}_{k,R}^2(\varvec{Y})\right) . \end{aligned}$$

So we may obtain the ridge estimates $\hat{\varvec{\gamma }}$ as above to estimate the variable-specific prior variances ${\hat{v}}_k=(\varvec{Z}_k\hat{\varvec{\gamma }})_+$, and transform these with the variance function to obtain the variable-specific prior parameters:

7 $$\begin{aligned} {\hat{\lambda }}_k = h^{-1}({\hat{v}}_k). \end{aligned}$$

This transformation can also be used to transform the prior variance estimates for the generalised additive co-data model in Equation (4) and for the shape-constrained co-data model in Eq. (5). Note, however, that the penalisation and constraints are applied to $\varvec{\gamma }$ and not to $\varvec{\lambda }$.

## Results

We include the full analyses with results in three vignettes corresponding to the three sections below; short examples (Additional file 1), simulation study (Additional file 2) and analysis example (Additional file 3). Here we summarise the main findings.

### Short examples

Use of **ecpc** for linear, generalised additive and shape-constrained additive co-data models is demonstrated in short examples. Besides, class-specific methods from ‘ecpc’ and transformation from ridge to elastic net penalties are illustrated.

### Simulation study

#### Estimation and prediction performance of various co-data models

**Fig. 4:**
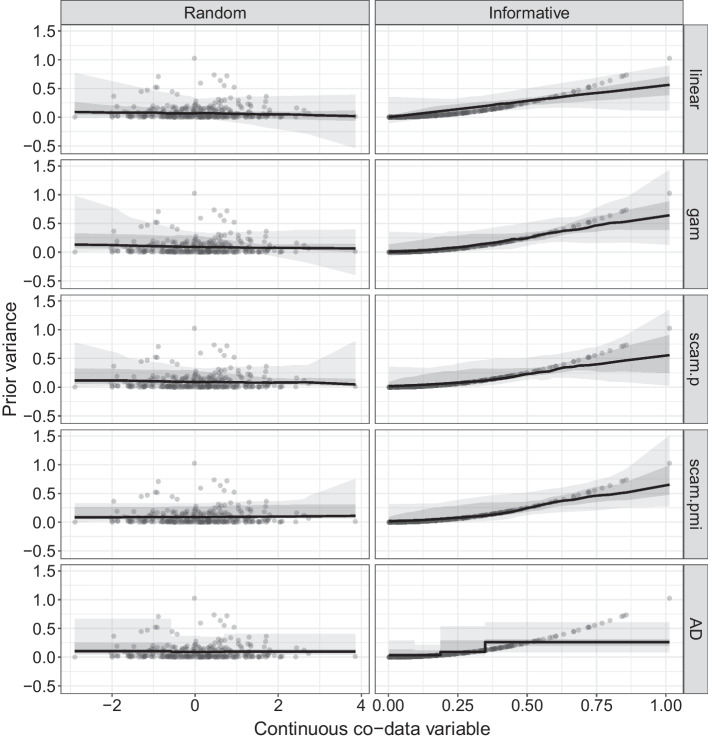
Simulation study based on 50 training and test sets and random co-data (left) or informative co-data (right). Estimated prior variance for various co-data models; (i) linear for linear co-data model; (ii) gam for generalised addive co-data model; (iii) scam.p for positive shape constrained co-data model; (iv) scam.pmi for positive and monotone increasing shape constrained co-data model, and (v) AD for adaptive discretisation. The lines indicate the pointwise median and the inner and outer shaded bands indicate the 25–75% and 5–95% quantiles respectively. Points indicate the true effect sizes $(\beta _k^0)^2$

The extension to **ecpc** proposes new co-data models for modelling continuous co-data in addition to the adaptive discretisation model proposed in the first version. We compare the newly proposed and former co-data models and a co-data agnostic ridge model in a simulation study. Figure 4 illustrates the prior variance estimates for the various co-data models. Results show that all co-data models lead to improved prediction performance compared to the co-data agnostic ridge model when co-data are informative and similar performance when co-data are random. The improvement for the newly proposed co-data models is slightly better than for the former, adaptive discretisation co-data model, as it better estimates the relation between the prior variance and co-data. Moreover, the newly proposed co-data models are around 3–6 times as fast as the former adaptive discretisation.

Besides, we compare robustness of the estimates for generalised additive co-data models for an increased number of splines and various methods for estimating the smoothing penalty, i.e. using random splits or any of the available methods in bam() in **mgcv** (“ML”, “fREML” and “GCV.Cp”). Using random splits leads to similar estimates as the methods “ML” and “fREML”, both for 20 and an increased number of 50 splines, while “GCV.Cp” leads to unstable estimates.

#### Variable selection compared to other methods

**Fig. 5:**
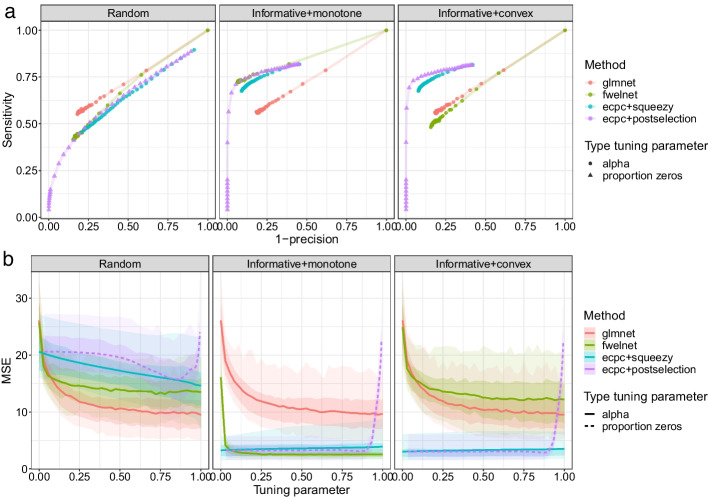
Simulation study for variable selection based on 50 training and test sets for various types of co-data. a) Average sensitivity and precision for several methods and various tuning parameters; b) Mean squared error prediction performance on the test data. The lines indicate the pointwise average and the inner and outer shaded bands indicate the 25–75% and 5-95% quantiles respectively

We compare variable selection of **ecpc** using posterior selection (ecpc+postselection) and elastic net penalties transformed with **squeezy** (ecpc+squeezy) with a co-data agnostic elastic net model (glmnet [23]) and feature-weighted elastic net (fwelnet [12]) in a simulation study. Results are shown in Fig. 5. Both variable selection methods implemented for **ecpc** show similar performance, besides differences resulting from the different type of tuning the level of sparsity. Results show that in the sparse setting, the co-data agnostic model glmnet outperforms the other co-data learnt methods when co-data are random, in contrast to the dense setting. When co-data are informative and the relation between the prior variances and co-data is monotone, the co-data learnt methods outperform glmnet, with fwelnet slightly outperforming **ecpc**. When co-data are informative and the relation between the prior variances and co-data is convex, **ecpc** outperforms fwelnet as the generalised additive co-data model is able to flexibly adapt to the non-exponential relation, whereas fwelnet is not.

#### Computation time and memory costs

**Fig. 6:**
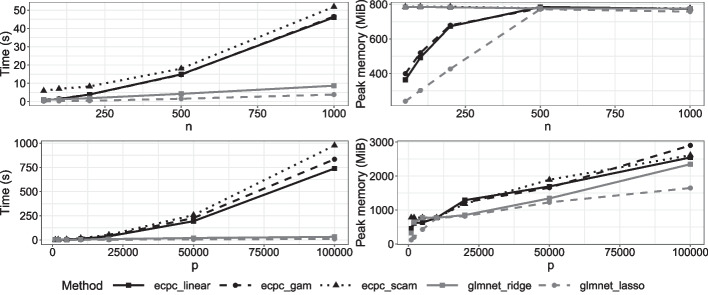
Simulation study for computation time and peak memory for varying numbers of samples *n* (*p* fixed at 5000) and number of variables *p* (*n* fixed at 200)

Figure 6 shows the computation time and peak memory used for various numbers of samples and variables and for the following models: **ecpc** with a linear co-data model, generalised additive co-data model (20 splines) or shape constrained additive co-data model (20 splines plus positivity constraint) and **glmnet** for a co-data agnostic ridge penalty or lasso penalty. As storing the memory-costly matrix $C\in {\mathbb {R}}^{p\times p}$ in Eq. (5) is avoided and only blocks of rows are stored, peak memory grows sub-quadratically with *p*.

### Analysis example

**Fig. 7:**
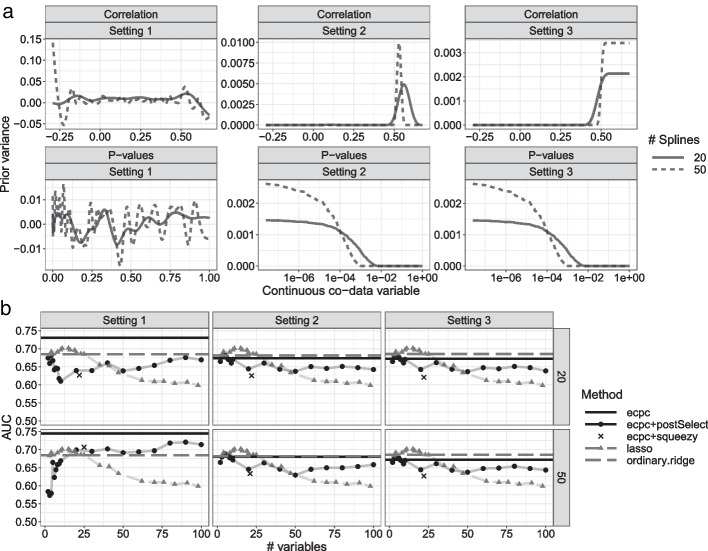
Data analysis example: **a** Estimated prior variance contributions of each co-data source, before multiplying with the co-data specific weight. Note that the *p* values are shown on the log-scale in Settings 2 and 3, to clearly show the non-zero peaks at the smallest *p* values; **b** corresponding prediction performance on the validation set for 20 or 50 spline basis functions. The settings correspond to different co-data models: (1) no constrains; (2) positive constrained shape; (3) positive and monotonically constrained shape

In [11, 13] we demonstrated the use of co-data to improve standard methods like ridge and lasso for several data sets. Here, we focus on a single data set with $n=133$ samples and $p=12838$ variables, that includes several types of co-data. We demonstrate the software on an application to the classification of lymph node metastasis from other types of cancer using high-dimensional RNA expression data. Three sources of co-data are available: categorical co-data for known signature genes, continuous co-data for cis-correlation between RNA and copy number and continuous co-data for *p* values from an external, similar study. More information on the data and details of the results are given in the vignette. We show results for several settings of co-data models and compare performances of dense and sparse models. Figure 7 shows the results for three settings: 1) a GAM, i.e. without constraints; 2) a SCAM with positivity constraints; 3) a SCAM with positivity and monotonicity constraints. Among the sparse models, using a generalised additive co-data model with 50 splines for the continuous co-data variables and posterior selection leads to the best performance on independent test data, though the simpler lasso model may be preferred as it shows competitive performance. Note, however, that lasso may render a rather unstable set of selected variables [24], and that the use of co-data improves this stability [11]. Overall, the dense model using a generalised additive co-data model with 50 splines shows the best prediction performance.

## Conclusions

We presented an extension to the **R**-package **ecpc** that accommodates linear co-data models, generalised additive co-data models and shape constrained additive co-data models for the purpose of high-dimensional prediction and variable selection. These co-data models are particularly useful for continuous co-data. The newly proposed co-data models are shown to run faster and lead to slightly better prediction performance when compared to adaptive discretisation. Moreover, the estimated variable-specific ridge penalties may be transformed to elastic net penalties with the **R**-package **squeezy** to allow for variable selection. We showed in a simulation study that this approach and the previously proposed posterior selection approach lead to similar performance, outperforming other methods when the effect sizes are (non-exponentially) related to the co-data. We have provided a vignette with several short examples to demonstrate general usage of the code (Additional file 1), a vignette to reproduce the simulation study (Additional file 2) and a vignette with an analysis example to a cancer genomics application (Additional file 3).

## Supplementary Information

Additional file 1. Vignette as pdf file to reproduce the short examples on general usage of the package.

Additional file 2. Vignette as pdf file to reproduce the simulation study.

Additional file 3. Vignette as pdf file to reproduce the analysis example.

## Data Availability

All three vignettes reproducing the simulations and examples, including data may be found on https://github.com/Mirrelijn/ecpc/vignettes. **Project name:** ecpc **Project home page:** https://github.com/Mirrelijn/ecpc **Operating system(s):** platform independent **Programming language:** **R** **Other requirements:** **R**$\ge 3.5.0$ **License:** GPL ($\ge 3$) **Any restrictions to use by non-academics:** none

## Abbreviations

GLM: Generalised linear models
GAM: Generalised additive model
SCAM: Shape-constrained additive model
