# Supplementary material for: ecpc: an R-package for generic co-data models for high-dimensional prediction
Source: BMC Bioinformatics. 2023 Apr 26;24:172. doi: 10.1186/s12859-023-05289-x (PMC10134536; doi:10.1186/s12859-023-05289-x)
Supplement: Supplementary file 1 — Additional file 1. Vignette as pdf file to reproduce the short examples on general usage of the package. [file 12859_2023_5289_MOESM1_ESM.pdf]

Vignette ecpc: general usage

Mirrelijn van Nee

Contents

Overview **1**
Model and co-data model 1
Model estimation 2
Installing and loading the R-package 2

Short examples co-data models **2**
Linear co-data model 2
Generalised additive co-data models 5
Shape-constrained co-data models 8

Short examples prediction and variable selection **10**

References **11**

Overview

This vignette presents short examples on the general usage of the R-package ecpc for high-dimensional prediction and variable selection, as supplementary material to [1].

Model and co-data model

We consider response data $\mathbf{Y} \in \mathbb{R}^n$, observed high-dimensional data $X \in \mathbb{R}^{n \times p}$ with $p \gg n$, which contain information on the n samples of $\mathbf{Y}$, and possibly multiple co-data matrices $Z^{(d)} \in \mathbb{R}^{p \times G_d}$, $d = 1, .., D$, which contain prior information on the p variables of X. We consider a generalised linear model for the response with canonical link function $g(\cdot)$, parameterised with regression coefficients $\beta \in \mathbb{R}^p$. Furthermore, we model the regression coefficients with a normal prior, corresponding to a ridge penalty, in which the prior variance is regressed on the co-data:

$$\begin{aligned} Y_i|\mathbf{X}_i, \beta &\overset{ind.}{\sim} \pi\left(Y_i|\mathbf{X}_i, \beta\right),\ E_{Y_i|\mathbf{X}_i,\beta}(Y_i) = g^{-1}(\mathbf{X}_i\beta),\ i = 1, .., n, \\ \beta_k &\overset{ind.}{\sim} N(0, v_k),\ v_k = \tau^2_{global} \sum_{d=1}^{D} w_d \mathbf{Z}_k^{(d)} \gamma^{(d)},\ k = 1, .., p, \end{aligned} \tag{1}$$

with $\mathbf{X}_i$ and $\mathbf{Z}_k$ the ith and k^{th} row of X and Z respectively, $\gamma^{(d)} \in \mathbb{R}^G$ the co-data variable weights for co-data matrix d, $\mathbf{w}$ the co-data matrix weights and τ^2_{global} a scaling factor which may improve numerical computations in practice.

The prior model on β assumes that the prior variance is a linear combination of the co-data variables. Besides a linear co-data model, the software allows for generalised additive co-data models and shape-constrained additive models.

Model estimation

The prior parameters are estimated with an empirical Bayes approach described in [1]. In short, they are estimated in three steps:

1. Global scaling parameter τ^2_{global} is estimated first by cross-validation or maximising the marginal likelihood
2. Co-data variable weights $\gamma^{(d)}$ are then estimated for $d = 1, .., D$, co-data sources separately, given the estimate for τ^2_{global} from the first step. Estimates may be penalised with an extra level of shrinkage, where the type of shrinkage can differ per co-data source to suit varying co-data models per co-data source, e.g. a linear co-data model for the first, generalised additive co-data model for the second and shape-constrained additive co-data model for the third.
3. Co-data source weights $\mathbf{w}$ are estimated similarly to the co-data variable weights given the estimates from the first and second step and constraining them to be positive.

After estimation of the prior parameters, the regression coefficients β are estimated by maximising the penalised likelihood.

Below, we illustrate the co-data models from the estimation of co-data variable weights in the second step. Where deemed unnecessary for the illustration, we consider the co-data models in their most basic form, so omit the global scaling parameter τ^2_{global} (i.e. set it to 1) and drop the annotation for the co-data source number $^{(d)}$.

Remark

The first version of ecpc, as described in [2] and with version number < 3.1, only handles (possibly overlapping) groups of variables. Co-data is then supplied in a list of group sets, in the argument `groupsets`. Continuous co-data variables may be handled by adaptive discretisation. The new version discussed here allows for both (undiscretised) continuous and grouped co-data. In the new version of ecpc, one needs to supply co-data as a list of co-data matrices in the argument `Z`. The function `createZforGroupset()` may be used to obtain the co-data matrix with dummy variables corresponding to a group set for grouped co-data.

Installing and loading the R-package

Install the latest version of the package with:

```
install.packages("ecpc")
```

Load the R-package with:

```
library(ecpc)
```

Short examples co-data models

Linear co-data model

The first co-data model is a basic linear relation between the prior variances and the co-data:

$$\mathbf{v} = Z\gamma.$$

The co-data variable weight estimates are an ordinary least squares solution to a linear system, with the estimated prior variance truncated to 0 as a variance cannot be negative:

$$\hat{\gamma} = \text{argmin}_{\gamma} ||(C \circ C)Z\gamma - \mathbf{b}||_2^2, \qquad \hat{\mathbf{v}} = (Z\hat{\gamma})_+, \tag{2}$$

with an expression of the matrix C and derivation of this estimate given in [1].

Interpretation co-data weights

The interpretation of the co-data weights γ (scaled by τ^2_{global}) is similar as in regular linear regression: when co-data variable Z_g increases with one unit, while the other co-data variables are kept fixed, then the prior variance increases with γ_g ($\gamma_g\tau^2_{global}$). Consequently, when the prior variance for the effect β_k of some variable X_k increases with γ_g ($\gamma_g\tau^2_{global}$), then the a priori expected squared effect size, $E(\beta_k^2) = v_k$, increases with γ_g ($\gamma_g\tau^2_{global}$). In other words, when we would compare the effect of two variables X_k and X_l with the same co-data values, except for one co-data variable which is one unit higher for X_k than for X_l, then we would a priori expect β_k^2 to be on average γ_g ($\gamma_g\tau^2_{global}$) larger than β_l^2.

Short example in ecpc

For this short example and the ones below, simulate some linear response data:

```
set.seed(1)
p <- 300 #number of covariates
n <- 100 #sample size training data set
n2 <-100 #sample size test data set
beta <- rnorm(p, mean=0, sd=0.1) #simulate effects
X <- matrix(rnorm(n*p, mean=0, sd=1), n, p) #observed training data
Y <- rnorm(n, mean = X%*%beta, sd=1) #response training data
X2 <- matrix(rnorm(n2*p, mean=0, sd=1), n, p) #observed test data
Y2 <- rnorm(n2, mean = X2%*%beta, sd=1) #response test data
```

As co-data, suppose that we have two co-data variables; one informative co-data variable containing the true absolute effect sizes and one non-informative co-data variable containing random normally distributed values:

```
Z1 <- abs(beta) #informative co-data
Z2 <- rnorm(p, mean=0, sd=1) #random, non-informative co-data
Z <- cbind(Z1, Z2) #(px2)-dimensional co-data matrix
```

Then we fit the linear co-data model and test the fit on the test data. Besides, we set `postselection=FALSE` to only estimate the dense model, without selecting variables a posteriori:

```
fit <- ecpc(Y, X, Z=list(Z), X2=X2, Y2=Y2, postselection=FALSE)
#> [1] "Estimate global tau^2 (equiv. global ridge penalty lambda)"
#> [1] "Estimate co-data weights and (if included) hyperpenalties with mgcv"
#> [1] "Estimate regression coefficients"
```

Note that the co-data matrix is provided in a list, as it is also possible to provide a list of multiple co-data matrices. This will be used to explicitly distinguish linear co-data variables from smooth or constrained ones, as exemplified below. The performance of the fit on the test data may be given for both the co-data learnt model fit with `ecpc()` and for the co-data agnostic model fit with one global ridge penalty:

```
print(fit$MSEecpc)
#> [1] 2.521757
```

```
print(fit$MSEridge)
#> [1] 2.889294
```

A (summary of) the fitted prior parameters, prior variances and regression coefficients can be retrieved by the methods `print()` and `summary()`:

```
print(fit)
#> ecpc fit
#>
#> Estimated co-data variable weights:
#> 0.2125547 -0.001632461
```

```
#>
#> Estimated co-data weights:
#> 1
summary(fit)
#> Summary estimated prior variances:
#>      Min.   1st Qu.    Median      Mean   3rd Qu.      Max.
#> 0.0000000 0.0000000 0.0008287 0.0068645 0.0106837 0.0471067
#>
#> Summary estimated regression coefficients:
#>      Min.   1st Qu.    Median      Mean   3rd Qu.      Max.
#> -0.251409  0.000000  0.000000  0.002368  0.002004  0.269845
#>
#> Estimated intercept:  0.07294278
```

Alternatively, the `plot()` method provides a graph of the regression coefficients and prior variances. If the R-packages ggplot2 [3] and ggpubr [4] are installed, the output looks as shown below, else a similar plot will be made with the base R plot-function.

```
plot(fit, show="coefficients")
#> Loading required namespace: ggpubr
```

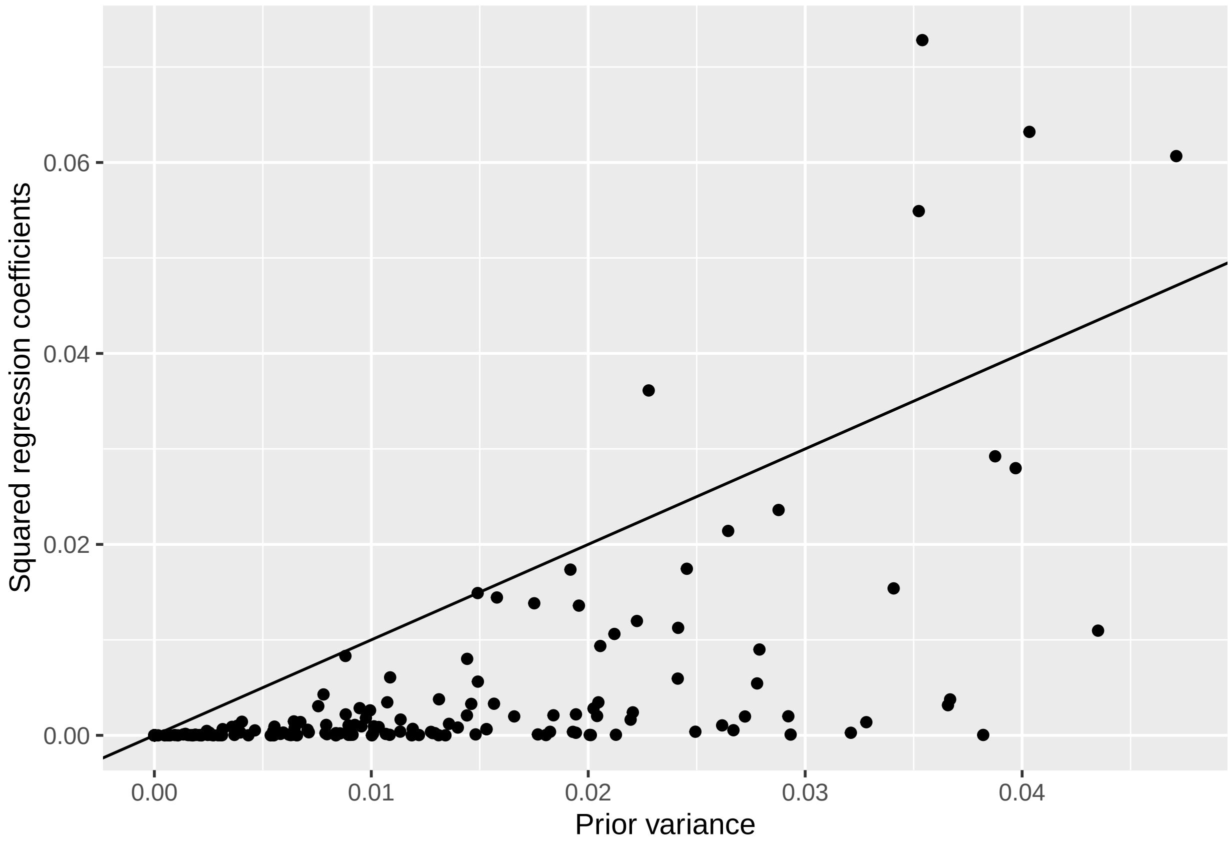


```
plot(fit, show="priorweights", Z=list(Z))
#> [1] "Prior variance contribution per group and co-data source is plotted."
#> [1] "Note that for splines, the original continuous co-data values should be provided in values"
```

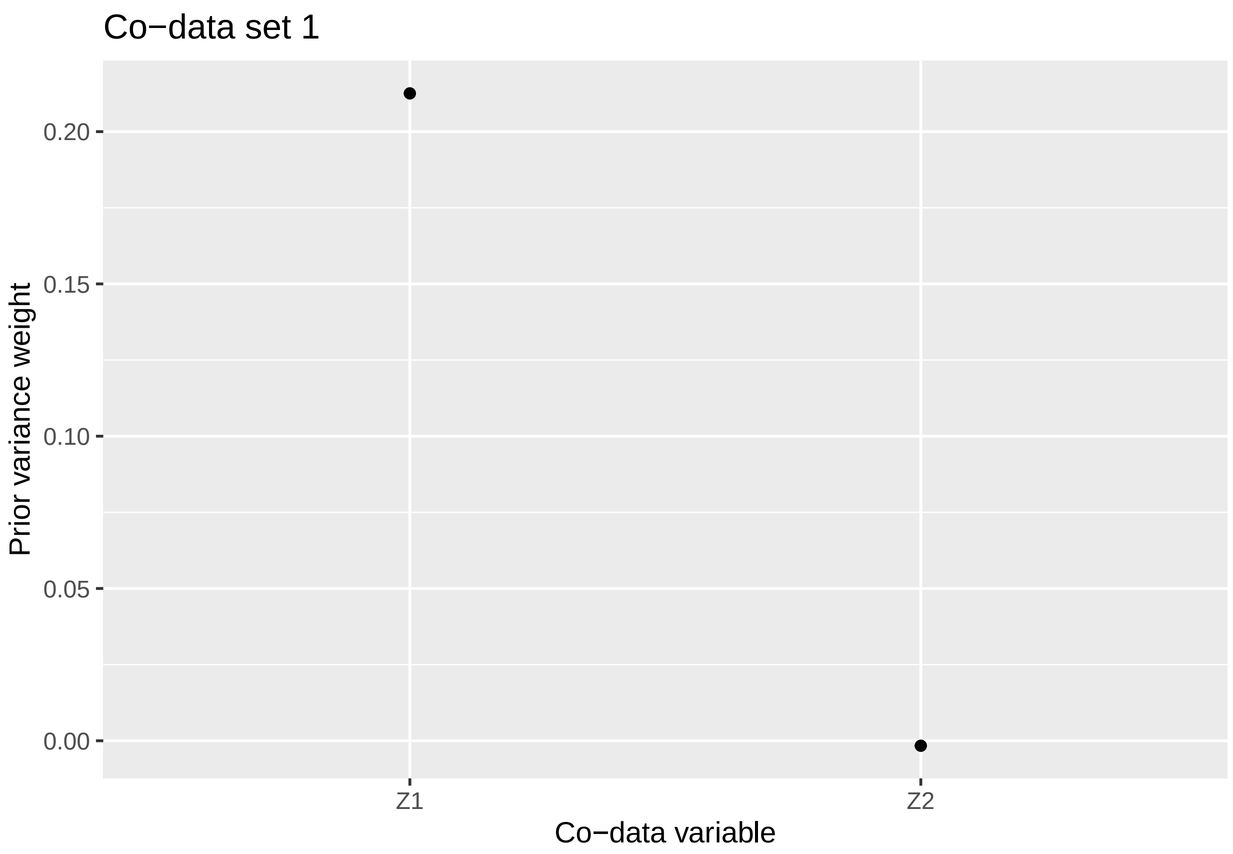

Lastly, the regression coefficients may be re-estimated for different prior parameters. First, the function `penalties()` may be used to change some prior parameters and retrieve the corresponding ridge penalties. Then, the method `coef()` re-estimates the regression coefficients given these penalties. For example, if one would alter the global level of regularisation by multiplying τ^2_{global} by 2:

```
new_penalties <- penalties(fit, tauglobal = fit$tauglobal * 2, Z=list(Z))
new_coefficients <- coef(fit, penalties=new_penalties, X=X, Y=Y)
```

Note that in general, however, altering the prior parameters by hand will not be needed as these parameters are optimised by the function `ecpc()`. The functions above, however, may be used to conveniently skip prior parameter estimation when prior parameters are known, e.g. when results have been saved and need to be checked quickly. For example, here we just set all prior parameters to one and only estimate the regression coefficients:

```
new_penalties2 <- penalties(tauglobal = 1, sigmahat = 1, gamma = c(1,1),
                            w = 1, Z=list(Z))
new_coefficients2 <- coef.ecpc(penalties=new_penalties2, X=X, Y=Y)
```

Generalised additive co-data models

Generalised additive models (GAMs), originally proposed in [5], have been widely applied to model non-linear relations. Applied here, we assume that the relation between the prior variance and co-data may be modeled by a sum of smooth functions, $s_1(\cdot), .., s_G(\cdot)$, of the co-data variables:

$$\mathbf{v} = \sum_{g=1}^{G} s_g(\mathbf{Z}_g).$$

In practice, the smooth functions are estimated by using a basis expansion to recast the problem into a linear model (as originally proposed by, for example, [6]). So, for a basis expansion consisting of J_g basis functions $\phi_{g,j}(\cdot)$, $j = 1, .., J_g$, for co-data variable $\mathbf{Z}_g$:

$$s_g(\mathbf{Z}_g) = \sum_{j=1}^{J_g} \phi_{g,j}(\mathbf{Z}_g)\gamma_{g,j} = \Phi_j \gamma_g, \qquad \mathbf{v} = \sum_{g=1}^{G} \Phi_j \gamma_g = Z_{GAM}\gamma_{GAM},$$

with $\Phi_g \in \mathbb{R}^{p \times J_g}$ the matrix of co-data variable vector $\mathbf{Z}_g \in \mathbb{R}^p$ evaluated in all J_g basis functions, $Z_{GAM} = [\Phi_1, .., \Phi_G]$ and $\gamma_{GAM} = (\gamma_1^T, .., \gamma_G^T)^T$.

The type and number of basis functions should in general be chosen such that they are flexible enough to approximate the underlying function well. To avoid overfitting for too many basis functions, the coefficients may be estimated by optimising the likelihood penalised by a smoothing penalty. While our software allows the user to supply any basis expansion, we focus here on the popular p-splines (see [7] for an introduction). This approach combines flexible spline basis functions with a quadratic smoothing penalty on the differences of the spline coefficients. So, the smoothing penalty is of the form $\gamma_{GAM}^T \left(\sum_g \lambda_g S_g\right) \gamma_{GAM}$, where the difference penalty matrix S_g smooths the non-linear function of the co-data variable $\mathbf{Z}_g$ and where λ_g is the corresponding smoothing penalty parameter. Hence, the least squares estimate for the linear co-data model is extended to the following estimate for the GAM coefficients in a non-linear co-data model:

$$\hat{\gamma}_{GAM} = \text{argmin}_{\gamma} \left\{ ||(C \circ C) Z_{GAM}\gamma - \mathbf{b}||_2^2 + \sum_{g=1}^{G} \lambda_g \gamma^T S_g \gamma \right\}, \; \hat{\mathbf{v}} = (Z_{GAM}\hat{\gamma}_{GAM})_+. \tag{3}$$

This least-squares equation is of a form also known as penalised signal regression [8] and can be solved by the function `gam()` (or `bam()` for big data) of the R-package mgcv, for example. This function also provides fast and stable estimation of the penalties λ_g [9]. Alternatively, when only one smoothing penalty matrix is provided, the smoothing penalty may be estimated by using random splits as proposed in [2].

Remark

Note that grouped co-data may be coded as group sets or as dummies in a co-data matrix `Z`. The former option, however, does not allow for a generalised ridge penalty, but for other penalties including the ordinary ridge and (hierarchical) lasso penalty.

Short example in ecpc

We continue with the simulated data from above. First, we use the helper functions `createZforSplines()` and `createS()` to create spline basis matrices and corresponding smoothing penalty matrices respectively. The degree of the spline functions and order of the penalty matrices are set to 3 and 2 by default, respectively. We set the number of splines to 20 for the first co-data variable and to 30 for the second in this example.

```
Z1.s <- createZforSplines(values=Z1, G=20, bdeg=3)
S1.Z1 <- createS(orderPen=2, G=20)
Z2.s <- createZforSplines(values=Z2, G=30, bdeg=3)
S1.Z2 <- createS(orderPen=2, G=30)
```

Before we fit the model, we first concatenate the two co-data matrices in a list. The variables of this list are always renamed such that the ith element is named `Zi`. The smoothing penalty matrices should be given in a separate argument `paraPen`, similar to the eponymous argument in `gam()`. Each element in this argument's list should match one of the names `Zi`, for which the corresponding smoothing matrix is given in `S1` (and optionally `S2`, `S3`, et cetera for multiple smoothing matrices for one co-data matrix).

```
Z.all <- list(Z1=Z1.s, Z2=Z2.s)
paraPen.all <- list(Z1=list(S1=S1.Z1), Z2=list(S1=S1.Z2))
```

Then we fit the model and test it on the test data as follows. Note that an intercept is included by default:

```
fit.gam <- ecpc(Y, X, Z = Z.all, paraPen = paraPen.all,
                intrcpt.bam=TRUE, X2=X2, Y2=Y2, postselection=FALSE)
#> [1] "Estimate global tau^2 (equiv. global ridge penalty lambda)"
#> [1] "Estimate co-data weights and (if included) hyperpenalties with mgcv"
#> [1] "Estimate regression coefficients"
fit.gam$MSEecpc
#> [1] 2.472784
```

The non-linear relation between the prior variance and each co-data source may again be plotted with the `plot()` method, either with the co-data spline variables on the x-axis, or the continuous co-data values on the x-axis:

```
plot(fit.gam, show="priorweights", Z=Z.all)
#> [1] "Prior variance contribution per group and co-data source is plotted."
#> [1] "Note that for splines, the original continuous co-data values should be provided in values"
```

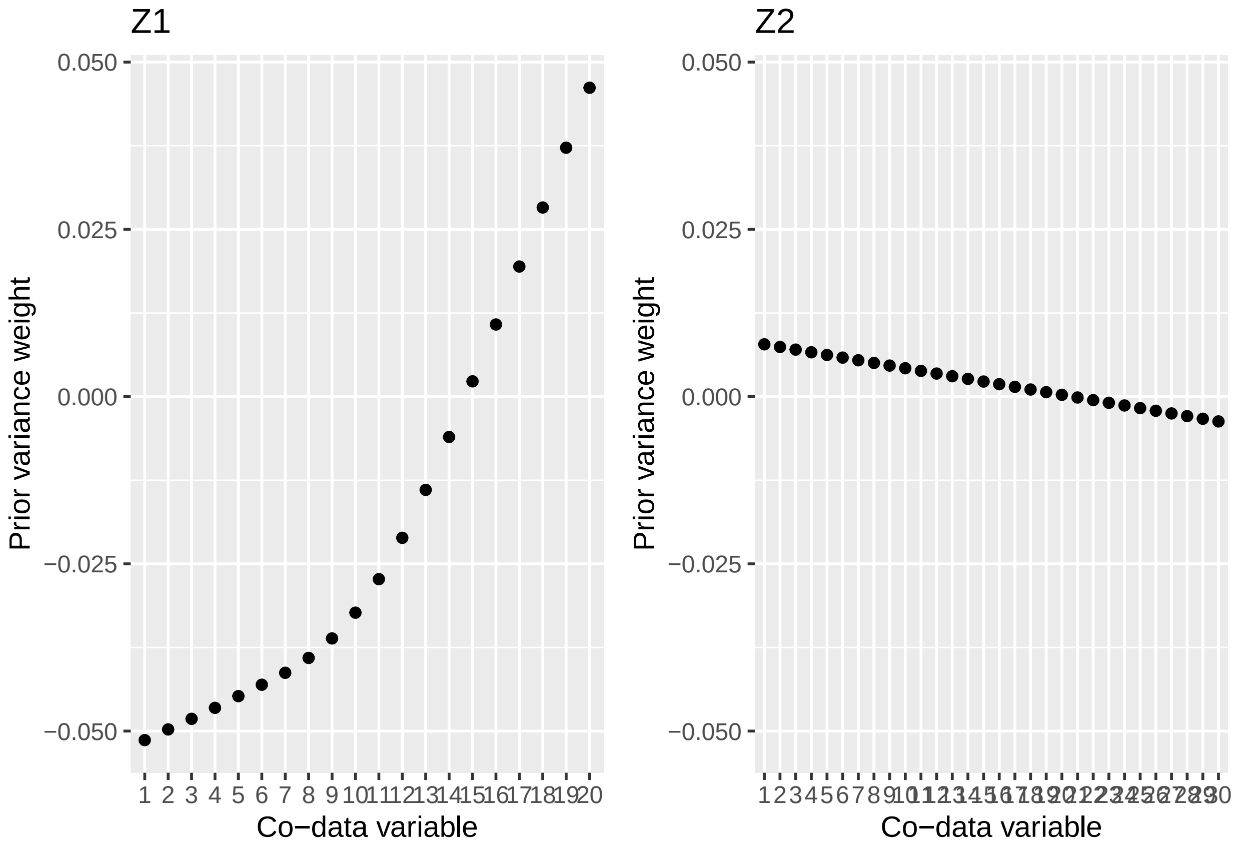


```
values <- list(Z1, Z2)
plot(fit.gam, show="priorweights", Z=Z.all, values = values)
#> [1] "Prior variance contribution per group/continuous values and co-data source is plotted."
```

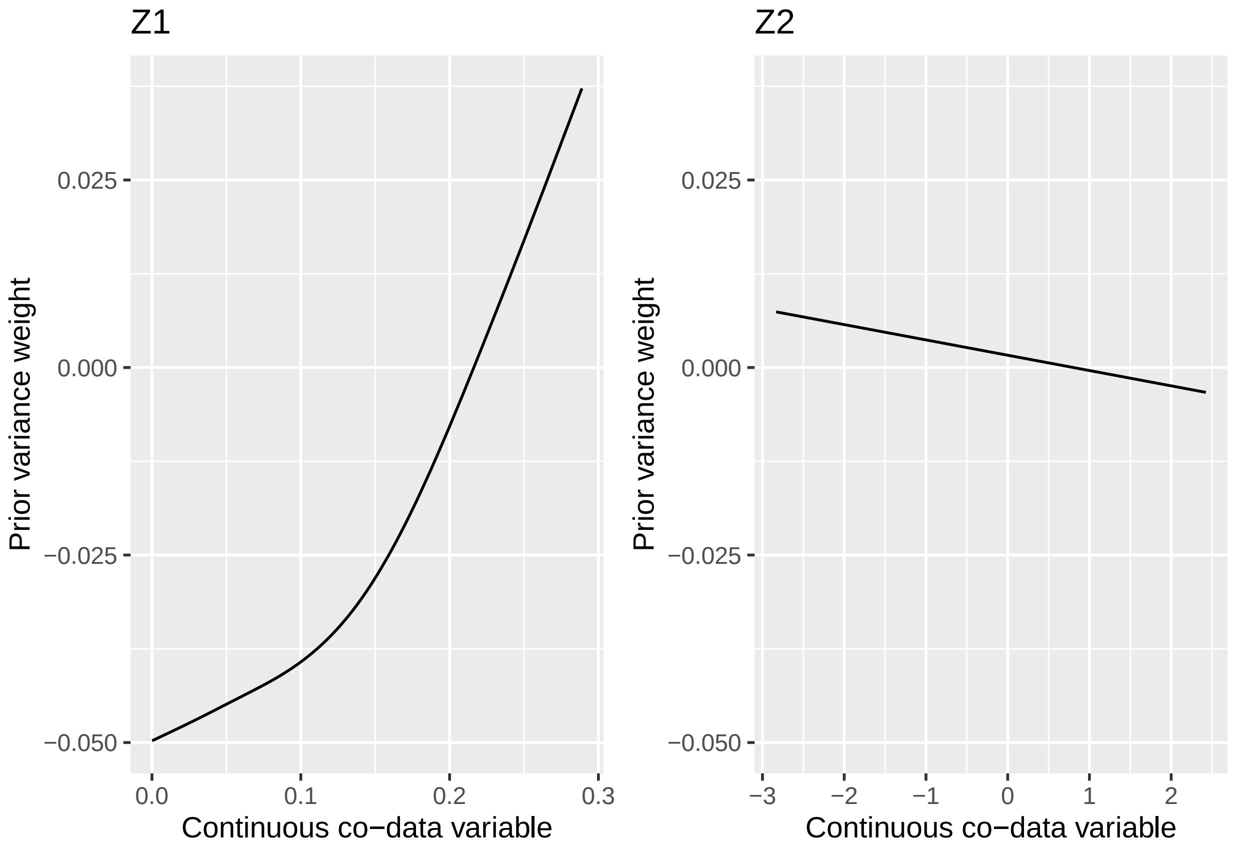

Alternatively, one may plot the non-linear relation directly. The spline variable coefficients are given in `fit\$gamma` for both co-data matrices and have an attribute `codataSource` to indicate for each coefficient to which co-data matrix it belongs. The non-linear relation of one co-data matrix is then plot as follows (output not shown):

```
codataNO <- attributes(fit.gam$gamma)$codataSource
i <- 2 #1 for informative, 2 for non-informative
sk <- as.vector(Z.all[[i]]%*%fit.gam$gamma[codataNO==i])*fit.gam$tauglobal
par(mfrow=c(1,1))
plot(Z[,i],sk)
```

Shape-constrained co-data models

Prior assumptions on the shape of the relation between the prior variance and co-data, such as monotonicity or convexity, may be imposed by constrained optimisation of spline coefficients [10]. So, for shape-constrained co-data models, we estimate the spline coefficients γ_g for each co-data variable $\mathbf{Z}_g$ and corresponding spline basis function matrix Φ_g subjectedto (in)equality constraints given in matrix $M_{(in)eq,g}$ and vector $\mathbf{b}_{(in)eq,g}$:

$$\begin{cases} \hat{\gamma}_g = \text{argmin}_{\gamma} \left\{ ||(C \circ C)\Phi_g \gamma - \mathbf{b}||_2^2 + \lambda_g \gamma^T S_g \gamma \right\} \\ \quad\quad \text{s.t. } M_{ineq,g}\gamma \leq \mathbf{b}_{ineq,g}, \;\; M_{eq,g}\gamma = \mathbf{b}_{eq,g} \end{cases} \tag{4}$$

Several shapes may be imposed by choosing M_{ineq} and b_{ineq} accordingly [10]. The helper function `createCon()` allows for the following examples:

- positivity may be imposed by constraining the spline coefficients to be positive;
- monotonically increasing (decreasing) may be imposed by constraining the first order differences $\gamma_{i+1} - \gamma_i$ to be positive (negative);

- convexity (concavity) may be imposed by constraining second order differences $\gamma_{i+2} - 2\gamma_{i+1} + \gamma_i$ to be positive (negative);
- any combination of the first three shapes may be imposed by combining the corresponding constraints.

Remark

Multiple co-data matrices $Z^{(1)}, .., Z^{(D)}$ may be provided in a list to the function `ecpc()`, or stacked and provided in a list of one co-data matrix $Z = [Z^{(1)}, .., Z^{(D)}]$. When the function `bam()` from mgcv is used, multiple smoothing parameters may be used for either representation, and are estimated jointly. After, the co-data variable weights γ are jointly estimated for all co-data matrices as well. As a result, the co-data weights $\mathbf{w}$ do not need to be estimated as they are implicitly accounted for in the joint estimate of γ. In contrast, when the random splitting is used, only one smoothing parameter per co-data matrix may be estimated. Therefore, the co-data matrix weights are estimated to combine multiple co-data matrices. By default, the function `ecpc()` uses `bam()` when co-data is provided in co-data matrices and no constraints are provided. This may be changed by setting `hypershrinkage="none"` when no penalty for the moment estimates is used or to `hypershrinkage="ridge"` when a generalised ridge penalty as in a GAM is used with random splits for estimating the penalty parameter. When constraints are provided, the function `ecpc()` automatically switches to the random splits.

Short example in ecpc

We continue the short example from above for shape-constrained functions. Say we would like to find a positive and monotonically increasing function for the first co-data variable, and a convex function for the second variable. We can use the helper function `createCon()` to obtain the constraint matrix M_{ineq} and vector $\mathbf{b}_{ineq}$ in the desired format for argument `paraCon`:

```
Con.Z1 <- createCon(G=20, shape="positive+monotone.i")
Con.Z2 <- createCon(G=30, shape="convex")
paraCon <- list(Z1=Con.Z1, Z2=Con.Z2)
```

Then we fit the model and plot the estimated shape-constrained functions as follows:

```
fit.scam <- ecpc(Y, X, Z = Z.all, paraPen = paraPen.all,
                  paraCon = paraCon, X2=X2, Y2=Y2, postselection=FALSE)
#> [1] "Estimate global tau^2 (equiv. global ridge penalty lambda)"
#> [1] "Co-data matrix 1: estimate hyperlambda for ridge+constraints hypershrinkage"
#> [1] "Estimate weights of co-data source 1"
#> [1] "Co-data matrix 2: estimate hyperlambda for ridge+constraints hypershrinkage"
#> [1] "Estimate weights of co-data source 2"
#> [1] "Estimate co-data source weights"
#> [1] "Estimate regression coefficients"
fit.scam$MSEecpc
#> [1] 2.490368
plot(fit.scam, show="priorweights", Z=Z.all, values=values)
#> [1] "Prior variance contribution per group/continuous values and co-data source is plotted."
```

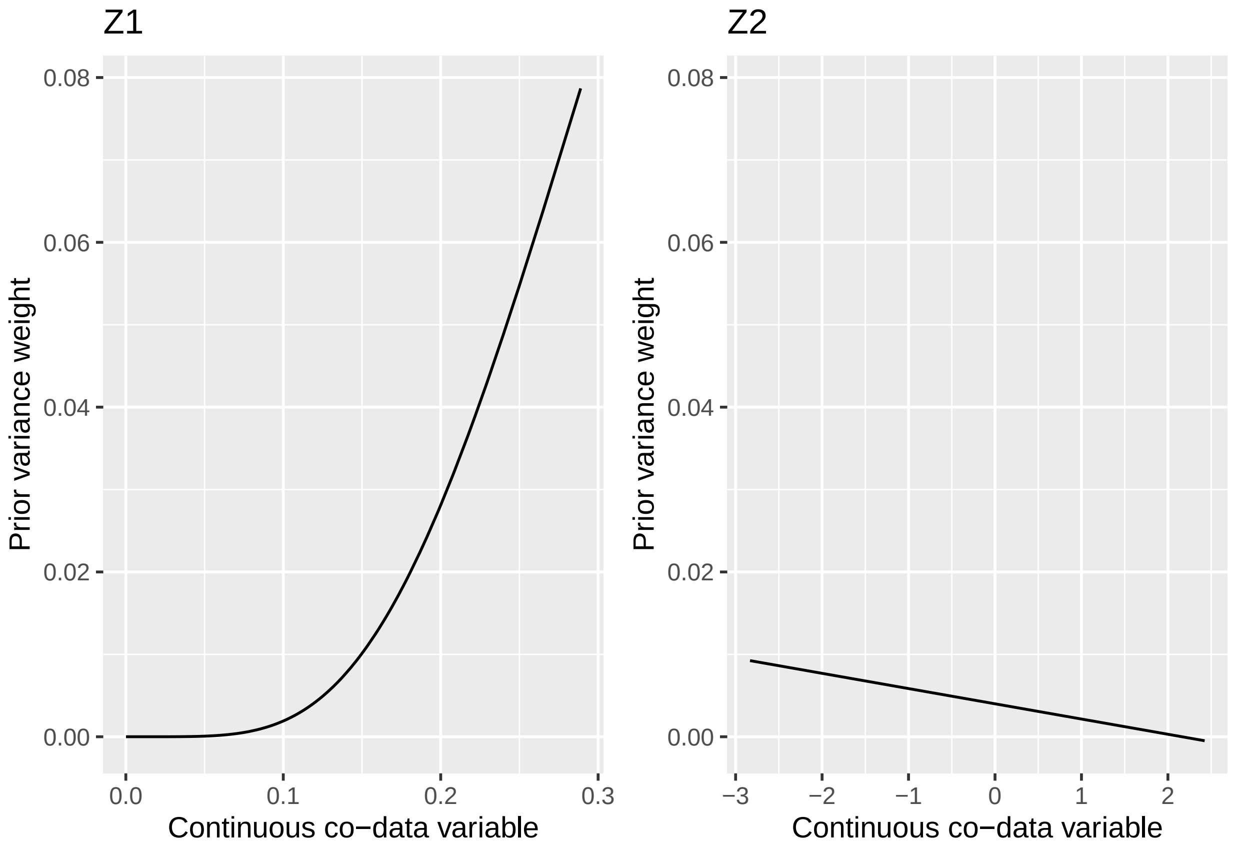

Note that an intercept is excluded by default, but that it can easily be included by appending a column of ones to Z.

Short examples prediction and variable selection

We continue with the simulated data from above. After fitting the model, responses may be predicted for new observed data given in `X2`:

```
Ypred <- predict(fit, X2=X2)
```

Variable selection may be done with posterior selection or by using the R-package squeezy to transform adaptive ridge penalties to elastic net penalties for simultaneous estimation and variable selection [1,11]. As example, we use the elastic net parameter $\alpha = 0.3$, for which we summarise the obtained elastic net penalties and regression coefficients. First, install the package if it is not available:

```
if(!requireNamespace("squeezy")) install.packages("squeezy")
```

Then, load the library and select variables using `squeezy()`:

```
library("squeezy")
fit.EN <- squeezy(Y, X, alpha=0.3, X2=X2, Y2=Y2, lambdas=fit$penalties)
summary(fit.EN$lambdapApprox) #transformed elastic net penalties
#>    Min. 1st Qu.  Median    Mean 3rd Qu.    Max.
#>   33.19  100.45  520.73     Inf     Inf     Inf
summary(fit.EN$betaApprox) #fitted elastic net regression coefficients
#>       Min.    1st Qu.     Median       Mean    3rd Qu.       Max.
#> -0.2677508  0.0000000  0.0000000  0.0008475  0.0000000  0.3351423
```

Alternatively, variables may be selected a posteriori with the function `postSelect()` on the model fit obtained with `ecpc()`. For example, if we want to select five or ten variables:

```
maxsel= c(5,10)
sparseModels <- postSelect(fit, X=X, Y=Y, maxsel=maxsel)
```

References

[1] van Nee, M.M., Wessels, L.F.A., van de Wiel, M.A.: ecpc: an R-package for generic co-data models for high-dimensional prediction

[2] van Nee, M.M., Wessels, L.F.A., van de Wiel, M.A.: Flexible co-data learning for high-dimensional prediction. Statistics in Medicine 40(26), 5910–5925 (2021)

[3] Wickham, H.: ggplot2: Elegant Graphics for Data Analysis. Springer, New York (2016). https://ggplot2.tidyverse.org

[4] Kassambara, A.: Package ggpubr. R package version 0.1 6 (2020)

[5] Hastie, T., Tibshirani, R.: Generalized additive models. Statistical Science 1(3), 297–318 (1986)

[6] Wahba, G.: Spline Bases, Regularization, and Generalized Cross Validation for Solving Approximation Problems with Large Quantities of Noisy Data. University of WISCONSIN, Madison (1980)

[7] Eilers, P.H., Marx, B.D.: Practical Smoothing: The Joys of P-splines. Cambridge University Press, Cambridge (2021)

[8] Marx, B.D., Eilers, P.H.: Generalized linear regression on sampled signals and curves: a p-spline approach. Technometrics 41(1), 1–13 (1999)

[9] Wood, S.N.: Fast stable restricted maximum likelihood and marginal likelihood estimation of semiparametric generalized linear models. Journal of the Royal Statistical Society: Series B (Methodological) 73(1), 3–36 (2011)

[10] Pya, N., Wood, S.N.: Shape constrained additive models. Stat. Comput. 25(3), 543–559 (2015)shape

[11] van Nee, M.M., van de Brug, T., van de Wiel, M.A.: Fast marginal likelihood estimation of penalties for group-adaptive elastic net. Journal of Computational and Graphical Statistics (2022)
