# Supplementary material for: ecpc: an R-package for generic co-data models for high-dimensional prediction
Source: BMC Bioinformatics. 2023 Apr 26;24:172. doi: 10.1186/s12859-023-05289-x (PMC10134536; doi:10.1186/s12859-023-05289-x)
Supplement: Supplementary file 2 — Additional file 2. Vignette as pdf file to reproduce the simulation study. [file 12859_2023_5289_MOESM2_ESM.pdf]

Vignette ecpc: simulation study

Mirrelijn van Nee

Contents

Overview 1

Simulation study 1: Estimation and prediction performance of various co-data models 2
- Run analysis . . . 2
- Results . . . 11

Simulation study 2: Variable selection compared to other methods 21
- Run analysis . . . 22
- Results . . . 30

Simulation study 3: Computation time and memory costs 34
- Load libraries . . . 34
- Generate data . . . 35
- Run analysis for varying n . . . 35
- Run analysis for varying p . . . 37
- Plot results . . . 39

References 44

Overview

This vignette can be used to reproduce the simulation results from [1].

Estimation and prediction performance have been compared for several methods in [2]. Here, we focus on continuous co-data to exemplify the newly proposed co-data models. First, we perform a simulation study to compare the estimates of the prior variance and prediction performance for different co-data models proposed here and the adaptive discretisation proposed in the first version of ecpc. Secondly, we perform a simulation study to compare different variable selection methods. Lastly, we perform a simulation study on computation time and peak memory costs of fitting various co-data models compared to a co-data agnostic ridge and lasso.

First, make sure the following libraries are installed:

```
if(!requireNamespace("ggplot2")) install.packages("ggplot2")
if(!requireNamespace("ggpubr")) install.packages("ggpubr")
if(!requireNamespace("ecpc")) install.packages("ecpc")
if(!requireNamespace("squeezy")) install.packages("squeezy")
if(!requireNamespace("dplyr")) install.packages("dplyr")
if(!requireNamespace("RColorBrewer")) install.packages("RColorBrewer")
if(!requireNamespace("foreach")) install.packages("foreach")
if(!requireNamespace("doParallel")) install.packages("doParallel")
if(!requireNamespace("glmnet")) install.packages("glmnet")
if(!requireNamespace("mvtnorm")) install.packages("mvtnorm")
if(!requireNamespace("pROC")) install.packages("pROC")
```

```r
if(!requireNamespace("devtools")) install.packages("devtools")
library(devtools)
if(!requireNamespace("fwelnet")) install_github("kjytay/fwelnet")
if(!requireNamespace("CoRF")) install_github("DennisBeest/CoRF")
if(!requireNamespace("ggh4x")) install_github("teunbrand/ggh4x")
```

The sections below on the three different simulation studies can be run independently from each other. Results are pre-saved and stored in the following folder:

```r
pathResults <- "./Results_sim_study/"
```

Simulation study 1: Estimation and prediction performance of various co-data models

We use the same simulation set-up as in [2] and simulate 50 training and test data sets for some true vector of regression coefficients $\beta^0 \in \mathbb{R}^{300}$. Again, we consider random and informative co-data, but now continuous versions of it:

- Random: generate standard normal co-data $Z_k \overset{i.i.d.}{\sim} N(0,1)$ for $k = 1,..,p$.
- Informative: use the true regression coefficients to inform the co-data; $Z_k = |\beta_k^0|$.

We compare the following co-data models:

- ridge: a co-data agnostic, global ridge penalty, corresponding to the co-data intercept only model. Any co-data method should outperform this baseline method when co-data is informative, and preferably not lose much when co-data is not informative;
- linear: a linear co-data model with an intercept and one (non-)informative co-data variable;
- gam: a generalised additive co-data model using p-splines of degree 3 and with difference penalty matrix of second order differences as suggested in [3]. We use 20 splines and the marginal likelihood method available in `bam()` from the mgcv R-package unless stated otherwise;
- scam.p: same as the generalised additive model but with shape constrained to be positive;
- scam.pmi: same as the generalised additive model but with shape constrained to be positive and monotonically increasing;
- AD: adaptive discretisation of the continuous co-data as proposed in [2]. We use a minimum of 20 variables in the smallest groups, which leads to seven hierarchical groups.

Run analysis

To rerun the analysis and generation of toy data, first set the following variables to `TRUE`:

```r
runLinear <- FALSE
runGAM <- FALSE
runSCAM <- FALSE
runSCAMpmi <- FALSE
runAD <- FALSE

generateData <- TRUE #set to true if not generated before
nSim <- 50 #set to lower number for quicker run
runParallel <- FALSE
```

Load libraries

Load the libraries needed for the analysis. Use multiple cores if available and if `runParallel=TRUE`.

```r
library(ecpc)
library(dplyr) #for data wrangling results
```

```
#>
#> Attaching package: 'dplyr'
#> The following objects are masked from 'package:stats':
#>
#>     filter, lag
#> The following objects are masked from 'package:base':
#>
#>     intersect, setdiff, setequal, union
library(ggplot2) #for plotting results
#> Warning: package 'ggplot2' was built under R version 4.2.2
library(RColorBrewer) #for plotting results
library(foreach) #for parallel computing
library(doParallel) #for parallel computing
#> Loading required package: iterators
#> Loading required package: parallel
library(scales)
#> Warning: package 'scales' was built under R version 4.2.2


if(runParallel){ #set to 1 to setup parallel backend to use many processors
  cores=detectCores()
  if(!("cl"%in%ls())){
    cl <- makeCluster(cores-1) #not to overload your computer
    registerDoParallel(cl)
  }
}
```

Generate data

Simulate multiple training and test data sets.

```
# Simulate toy data ------
p<-300 #number of covariates
n<-100 #sample size training data set
n2<-100 #sample size test data set
sigma <- 1
tauglobal <- 0.1 #prior variance

#simulate all betas i.i.d. from beta_k~N(mean=0,sd=sqrt(0.1)):
set.seed(7474)
Dat <- list()
Dat$beta <- rnorm(p,sd=sqrt(tauglobal))
Dat$Xctd <- matrix(rnorm(n*p) ,n,p)
Dat$X2ctd <- matrix(rnorm(n*p) ,n,p)
Dat$Y <- rnorm(n,mean=c(Dat$Xctd%*%Dat$beta),sd=sigma)

if(generateData){
  AllData <- list()
  for(i in 1:nSim){
    AllData[[i]] <- list()
    AllData[[i]]$beta <- Dat$beta
    AllData[[i]]$Xctd <- matrix(rnorm(n*p) ,n,p)
    AllData[[i]]$X2ctd <- matrix(rnorm(n*p) ,n,p)
    AllData[[i]]$Y <- rnorm(n,mean=c(AllData[[i]]$Xctd%*%AllData[[i]]$beta),sd=sigma)
```

```
    AllData[[i]]$Y2 <- rnorm(n2,mean=c(AllData[[i]]$X2ctd%*%AllData[[i]]$beta),sd=sigma)
  }
  save(Dat,AllData,file=paste(pathResults,"SimData.Rdata",sep=''))
}else{
  load(paste(pathResults,"SimData.Rdata",sep=''))
}
```

Configure co-data

For the co-data models using splines, we use either 20 or 50 splines. We make lists of the co-data sources:

```
#Co-data settings----
G <- c(20,50) #number of splines
Z.all <- list()
ZI.all <- list() #co-data matrix with intercept
Zs.all <- list()
S1 <- list()
Con.p <- list() #positivity constraints
Con.pmi <- list() #positivity and monotonically increasing constraints
#setting 1: non-informative, unequally spaced
Z.all[["noninformative"]] <- rnorm(p) #for linear co-data model
ZI.all[["noninformative"]] <- cbind(rep(1,p), Z.all[["noninformative"]])
#for generalised additive co-data model:
for(g in 1:length(G)){
  Zs.all[[g]] <- list()
  Zs.all[[g]][["noninformative"]] <- createZforSplines(
    values=Z.all[["noninformative"]], G=G[g], bdeg=3)
  #create 2nd order difference penalty matrix (same for all co-data)
  S1[[g]] <- createS(orderPen=2, G=G[g])

  Con.p[[g]] <- createCon(G=G[g], shape="positive") #create constraints
  Con.pmi[[g]] <- createCon(G=G[g], shape="positive+monotone.i") #create constraints
}
#plot(Z.all[[1]],Dat$beta^2)

#setting 2: informative, unequally spaced, information at edge
Z.all[["size.edge"]] <- abs(Dat$beta)
ZI.all[["size.edge"]] <- cbind(rep(1,p), Z.all[["size.edge"]])
#for generalised additive co-data model:
for(g in 1:length(G)){
  Zs.all[[g]][["size.edge"]] <- createZforSplines(values=Z.all[["size.edge"]],
                                                  G=G[g], bdeg=3)
}
#plot(Z.all[[2]],Dat$beta^2)

#setting 3: non-informative, transformed for equally spacing
Z.all[["noninformative+transformed"]] <- order(order(Z.all[["noninformative"]],
                                                     decreasing=FALSE),
                                               decreasing=FALSE)
ZI.all[["noninformative+transformed"]] <- cbind(rep(1,p),
                                                Z.all[["noninformative+transformed"]])
#for generalised additive co-data model:
for(g in 1:length(G)){
  Zs.all[[g]][["noninformative+transformed"]] <- createZforSplines(
```

```
    values=Z.all[["noninformative+transformed"]], G=G[g], bdeg=3)
}
#plot(Z.all[[3]],Dat$beta^2)

#setting 4: informative, transformed for equally spacing
Z.all[["size.edge+transformed"]] <- order(order(abs(Dat$beta),decreasing=FALSE),
                                          decreasing=FALSE)
ZI.all[["size.edge+transformed"]] <- cbind(rep(1,p), Z.all[["size.edge+transformed"]])
for(g in 1:length(G)){
  Zs.all[[g]][["size.edge+transformed"]] <- createZforSplines(
    values=Z.all[["size.edge+transformed"]], G=G[g], bdeg=3)
}
#plot(Z.all[[4]],Dat$beta^2)

#Compare with adaptive discretisation of continuous co-data----
groupsets <- list()
hierarchy.grouplevel <- list()
Z.AD <- list() #useful for computing prior variances

#setting 1: non-informative hierarchical discretisation
#Use adaptive discretisation to find a good discretisation of the continuous co-data;
# discretise in groups of covariates of various sizes:
groupsets[["noninformative"]] <- splitMedian(values=Z.all[["noninformative"]],index = 1:p,
                                             minGroupSize = 50,split="both")
Z.AD[["noninformative"]] <- createZforGroupset(groupsets[["noninformative"]])
# and obtain group set on group level that defines the hierarchy:
hierarchy.grouplevel[["noninformative"]] <- obtainHierarchy(
  groupset = groupsets[["noninformative"]])

#setting 2: informative hierarchical discretisation
#Use adaptive discretisation to find a good discretisation of the continuous co-data;
# discretise in groups of covariates of various sizes:
groupsets[["size.edge"]] <- splitMedian(values=Z.all[["size.edge"]],index = 1:p,
                                        minGroupSize = 50,split="both")
Z.AD[["size.edge"]] <- createZforGroupset(groupsets[["size.edge"]])
# and obtain group set on group level that defines the hierarchy:
hierarchy.grouplevel[["size.edge"]] <- obtainHierarchy(
  groupset = groupsets[["noninformative"]])
```

Linear co-data model

To rerun the analysis, run the following (collapsed) code block:

```
#Fit ecpc on simulated data sets: linear co-data model----
fname <- paste(pathResults,"SimResAppNoteLinear",".Rdata",sep="")

if(runLinear){
  #df <- data.frame()
  #for(sim in 1:nSim){
  finalMatrix <- foreach(sim=1:nSim, .combine=rbind,
                         .packages = c("ecpc")) %dopar% {
                           df <- data.frame()
                           for(setting in 1:4){
```

```
                              for(method in "none"){#c("ML","fREML","GCV.Cp")){
                                tic<-proc.time()[[3]]
                                fit <- ecpc(Y=AllData[[sim]]$Y,X=AllData[[sim]]$Xctd,
                                            Z=ZI.all[setting],
                                            #bam.method=method,intrcpt.bam = F,
                                            model="linear",maxsel=c(5,10,15,20),
                                            Y2=AllData[[sim]]$Y2,X2=AllData[[sim]]$X2ctd,
                                            est_beta_method = "multiridge")
                                toc <- proc.time()[[3]]-tic

                                vk <- (ZI.all[[setting]]%*%fit$gamma )*fit$tauglobal


                                temp<-data.frame(vkfit=vk,truebeta=Dat$beta,truevk=Dat$beta^2)
                                temp$Z <- Z.all[[setting]]
                                temp$Time <- toc
                                temp$Covariate <- 1:p
                                temp$G <- 2
                                temp$setting <- setting
                                temp$bam.method <- method
                                temp$method <- "linear"
                                temp$MSEridge <- fit$MSEridge
                                temp$MSEecpc <- fit$MSEecpc
                                temp$Sim <- sim
                                temp$Codata <- names(Z.all)[setting]
                                temp$Transform <- F; if(setting>=3) temp$Transform <- T
                                temp$ZUntransformed <- Z.all[[setting]]
                                if(setting>=3) temp$ZUntransformed <- Z.all[[setting-2]]
                                df <- rbind(df,temp)
                              }
                            }
                            list("df"=df)
                          }
  #str(finalMatrix)
  df <- finalMatrix[[1]]; for(i in 2:nSim) df <- rbind(df,finalMatrix[[i]])
  save(finalMatrix,df,file=fname)
}
```

Generalised additive co-data model

To rerun the analysis, run the following (collapsed) code block:

```
#Fit ecpc on simulated data sets: generalised additive co-data model----
fname <- paste(pathResults,"SimResAppNoteGAM.Rdata",sep="")
print(fname)

if(runGAM){
  #df <- data.frame()
  #for(sim in 1:nSim){
  finalMatrix <- foreach(sim=1:nSim, .combine=rbind,
                        .packages = c("ecpc")) %dopar% {
                          df <- data.frame()
                          for(setting in 1:4){
                            for(g in 1:length(G)){
```

```r
                    for(method in c("splits","ML","fREML","GCV.Cp")){
                      tic<-proc.time()[[3]]

                      if(method=="splits"){
                        fit <- ecpc(Y=AllData[[sim]]$Y,X=AllData[[sim]]$Xctd,
                                    Z=Zs.all[[g]][setting],
                                    paraPen=list(Z1=list(S1=S1[[g]])),
                                    intrcpt.bam = F,
                                    model="linear",maxsel=c(5,10,15,20),
                                    Y2=AllData[[sim]]$Y2,X2=AllData[[sim]]$X2ctd,
                                    hypershrinkage = "ridge",
                                    est_beta_method = "multiridge")
                      }else{
                        fit <- ecpc(Y=AllData[[sim]]$Y,X=AllData[[sim]]$Xctd,
                                    Z=Zs.all[[g]][setting],
                                    paraPen=list(Z1=list(S1=S1[[g]])),
                                    bam.method=method,intrcpt.bam = F,
                                    model="linear",maxsel=c(5,10,15,20),
                                    Y2=AllData[[sim]]$Y2,X2=AllData[[sim]]$X2ctd,
                                    est_beta_method = "multiridge")
                      }

                      toc <- proc.time()[[3]]-tic

                      vk <- (Zs.all[[g]][[setting]]%*%fit$gamma )*fit$tauglobal
                      #plot(Z.all[[setting]],vk)



                      temp<-data.frame(vkfit=vk,truebeta=Dat$beta,
                                       truevk=Dat$beta^2)
                      temp$Z <- Z.all[[setting]]
                      temp$Time <- toc
                      temp$Covariate <- 1:p
                      temp$G <- G[g]
                      temp$setting <- setting
                      temp$bam.method <- method
                      temp$method <- "gam"
                      temp$MSEridge <- fit$MSEridge
                      temp$MSEecpc <- fit$MSEecpc
                      temp$Sim <- sim
                      temp$Codata <- names(Z.all)[setting]
                      temp$Transform <- F; if(setting>=3) temp$Transform <- T
                      temp$ZUntransformed <- Z.all[[setting]]
                      if(setting>=3) temp$ZUntransformed <- Z.all[[setting-2]]
                      df <- rbind(df,temp)
                    }
                  }
                }
                list("df"=df)
              }
#str(finalMatrix)
df <- finalMatrix[[1]]; for(i in 2:nSim) df <- rbind(df,finalMatrix[[i]])
```

```
  save(finalMatrix,df,file=fname)
}
```

Positive shape-constrained additive co-data model

To rerun the analysis, run the following (collapsed) code block:

```
#Fit ecpc on simulated data sets: positive constrained generalised additive co-data model----
fname <- paste(pathResults,"SimResAppNoteSCAMp.Rdata",sep="")
print(fname)

if(runSCAM){
  #df <- data.frame()
  #for(sim in 1:nSim){
  finalMatrix <- foreach(sim=1:nSim, .combine=rbind,
                         .packages = c("ecpc")) %dopar% {
                           df <- data.frame()
                           for(setting in 1:4){
                             for(g in 1:length(G)){
                               for(method in c("splits")){
                                 tic<-proc.time()[[3]]

                                 fit <- ecpc(Y=AllData[[sim]]$Y,X=AllData[[sim]]$Xctd,
                                             Z=Zs.all[[g]][setting],
                                             paraPen=list(Z1=list(S1=S1[[g]])),
                                             paraCon = list(Z1=Con.p[[g]]),
                                             intrcpt.bam = F,
                                             model="linear",maxsel=c(5,10,15,20),
                                             Y2=AllData[[sim]]$Y2,X2=AllData[[sim]]$X2ctd,
                                             est_beta_method = "multiridge")
                                 toc <- proc.time()[[3]]-tic

                                 vk <- (Zs.all[[g]][[setting]]%*%fit$gamma )*fit$tauglobal
                                 plot(Z.all[[setting]],vk)



                                 temp<-data.frame(vkfit=vk,truebeta=Dat$beta,
                                                  truevk=Dat$beta^2)
                                 temp$Z <- Z.all[[setting]]
                                 temp$Time <- toc
                                 temp$Covariate <- 1:p
                                 temp$G <- G[g]
                                 temp$setting <- setting
                                 temp$bam.method <- method
                                 temp$method <- "scam.p"
                                 temp$MSEridge <- fit$MSEridge
                                 temp$MSEecpc <- fit$MSEecpc
                                 temp$Sim <- sim
                                 temp$Codata <- names(Z.all)[setting]
                                 temp$Transform <- F; if(setting>=3) temp$Transform <- T
                                 temp$ZUntransformed <- Z.all[[setting]]
                                 if(setting>=3) temp$ZUntransformed <- Z.all[[setting-2]]
                                 df <- rbind(df,temp)
```

```
                            }
                          }
                        }
                        list("df"=df)
                      }
  #str(finalMatrix)
  df <- finalMatrix[[1]]; for(i in 2:nSim) df <- rbind(df,finalMatrix[[i]])
  save(finalMatrix,df,file=fname)
}
```

Positive and monotone increasing shape-constrained additive co-data model

To rerun the analysis, run the following (collapsed) code block:

```
#Fit ecpc on simulated data sets: positive+monotone increasing constrained
#generalised additive co-data model----
fname <- paste(pathResults,"SimResAppNoteSCAMpmi.Rdata",sep="")
print(fname)

if(runSCAMpmi){
  #df <- data.frame()
  #for(sim in 1:nSim){
  finalMatrix <- foreach(sim=1:nSim, .combine=rbind,
                         .packages = c("ecpc")) %dopar% {
                           df <- data.frame()
                           for(setting in 1:4){
                             for(g in 1:length(G)){
                               for(method in c("splits")){
                                 tic<-proc.time()[[3]]

                                 fit <- ecpc(Y=AllData[[sim]]$Y,X=AllData[[sim]]$Xctd,
                                             Z=Zs.all[[g]][setting],
                                             paraPen=list(Z1=list(S1=S1[[g]])),
                                             paraCon = list(Z1=Con.pmi[[g]]),
                                             intrcpt.bam = F,
                                             model="linear",maxsel=c(5,10,15,20),
                                             Y2=AllData[[sim]]$Y2,X2=AllData[[sim]]$X2ctd,
                                             est_beta_method = "multiridge")
                                 toc <- proc.time()[[3]]-tic

                                 vk <- (Zs.all[[g]][[setting]]%*%fit$gamma )*fit$tauglobal
                                 #plot(Z.all[[setting]],vk)



                                 temp<-data.frame(vkfit=vk,truebeta=Dat$beta,
                                                  truevk=Dat$beta^2)
                                 temp$Z <- Z.all[[setting]]
                                 temp$Time <- toc
                                 temp$Covariate <- 1:p
                                 temp$G <- G[g]
                                 temp$setting <- setting
                                 temp$bam.method <- method
                                 temp$method <- "scam.pmi"
```

```
                                    temp$MSEridge <- fit$MSEridge
                                    temp$MSEecpc <- fit$MSEecpc
                                    temp$Sim <- sim
                                    temp$Codata <- names(Z.all)[setting]
                                    temp$Transform <- F; if(setting>=3) temp$Transform <- T
                                    temp$ZUntransformed <- Z.all[[setting]]
                                    if(setting>=3) temp$ZUntransformed <- Z.all[[setting-2]]
                                    df <- rbind(df,temp)
                                  }
                                }
                              }
                              list("df"=df)
                            }
  #str(finalMatrix)
  df <- finalMatrix[[1]]; for(i in 2:nSim) df <- rbind(df,finalMatrix[[i]])
  save(finalMatrix,df,file=fname)
}
```

Adaptive discretisation co-data model

To rerun the analysis, run the following (collapsed) code block:

```
#Fit ecpc on simulated data sets: adaptive discretisation co-data model----
fname <- paste(pathResults,"SimResAppNoteAD.Rdata",sep="")
print(fname)

if(runAD){
  #df <- data.frame()
  #for(sim in 1:nSim){
  finalMatrix <- foreach(sim=1:nSim, .combine=rbind,
                         .packages = c("ecpc")) %dopar% {
                           df <- data.frame()
                           for(setting in 1:2){
                             #for(g in 1:length(G)){
                             for(method in c("splits")){
                               tic<-proc.time()[[3]]

                               fit <- ecpc(Y=AllData[[sim]]$Y,X=AllData[[sim]]$Xctd,
                                           groupsets=groupsets[setting],
                                           groupsets.grouplvl = hierarchy.grouplevel[setting],
                                           hypershrinkage = "hierLasso,ridge",
                                           model="linear",maxsel=c(5,10,15,20),
                                           Y2=AllData[[sim]]$Y2,X2=AllData[[sim]]$X2ctd,
                                           est_beta_method = "multiridge")
                               toc <- proc.time()[[3]]-tic

                               vk <- as.vector(Z.AD[[setting]]%*%fit$gamma*fit$tauglobal)
                               #plot(Z.all[[setting]],vk)


                               temp<-data.frame(vkfit=vk,truebeta=Dat$beta,
                                                truevk=Dat$beta^2)
                               temp$Z <- Z.all[[setting]]
                               temp$Time <- toc
```

```
                                temp$Covariate <- 1:p
                                temp$G <- length(groupsets[[setting]])
                                temp$setting <- setting
                                temp$bam.method <- method
                                temp$method <- "AD"
                                temp$MSEridge <- fit$MSEridge
                                temp$MSEecpc <- fit$MSEecpc
                                temp$Sim <- sim
                                temp$Codata <- names(Z.all)[setting]
                                temp$Transform <- F; if(setting>=3) temp$Transform <- T
                                temp$ZUntransformed <- Z.all[[setting]]
                                if(setting>=3) temp$ZUntransformed <- Z.all[[setting-2]]
                                df <- rbind(df,temp)
                              }
                              #}
                            }
                            list("df"=df)
                          }
  #str(finalMatrix)
  df <- finalMatrix[[1]]; for(i in 2:nSim) df <- rbind(df,finalMatrix[[i]])
  save(finalMatrix,df,file=fname)
}
```

Results

Load results

First, set some plotting parameters and load the pre-saved results:

```
#Plots: general parameters----
wdth<-600
hght<-wdth*5/8
wdthpdf <- wdth/75
hghtpdf <- hght/75
ts <- 16 #basis text size in figures
ls <- 1.5 #basis line size in figures
ps <- 2 #basis point size in figures
sz <- 2 #point size
strk <- 1.5 #stroke size
palette <- "Dark2"
colpal <- "Dark2"
colsfill <- brewer.pal(3,"Dark2")[1:2]
colsAUC <- brewer.pal(10,"RdYlBu")
colsAUC <- seq_gradient_pal(low="black",high="white")((0:4)/5)



#Load data for plots----
#All estimates and predictions
dfAll <- data.frame()
#linear
fname <- paste(pathResults,"SimResAppNoteLinear.Rdata",sep="")
load(fname)
dfAll <- rbind(dfAll,df)
```

```
#gam
fname <- paste(pathResults,"SimResAppNoteGAM",".Rdata",sep="")
load(fname)
dfAll <- rbind(dfAll,df)
#scam positive
fname <- paste(pathResults,"SimResAppNoteSCAMp",".Rdata",sep="")
load(fname)
dfAll <- rbind(dfAll,df)
#scam positive+monotone increasing
fname <- paste(pathResults,"SimResAppNoteSCAMpmi",".Rdata",sep="")
load(fname)
dfAll <- rbind(dfAll,df)
#hierarchical adaptive discretisation
fname <- paste(pathResults,"SimResAppNoteAD",".Rdata",sep="")
load(fname)
dfAll <- rbind(dfAll,df)

dfAll$G <- as.factor(dfAll$G)
dfAll$method <- factor(dfAll$method, levels=unique(dfAll$method),
                       labels=unique(dfAll$method))
dfAll$Codata <- factor(dfAll$Codata,levels=unique(dfAll$Codata),
                       labels=c("Random","Informative",
                                "Random+transformed","Informative+transformed"))
dfAll$Codata2 <- factor(dfAll$Codata,levels=unique(dfAll$Codata),
                        labels=c("Random","Informative",
                                 "Random","Informative"))

#summarise estimated variance pointwise for continuous co-data per setting
dfEst <- dfAll %>% group_by(G,method,Codata,Covariate,bam.method,
                            Z, ZUntransformed,Transform,Codata2) %>%
  summarise(meanVk = mean(vkfit),q50Vk = quantile(vkfit,0.5),
            q95Vk = quantile(vkfit,0.95),q05Vk = quantile(vkfit,0.05),
            q75Vk = quantile(vkfit,0.75),q25Vk = quantile(vkfit,0.25),
            truevk = mean(truevk)) %>% ungroup()
#> `summarise()` has grouped output by 'G', 'method', 'Codata', 'Covariate',
#> 'bam.method', 'Z', 'ZUntransformed', 'Transform'. You can override using the
#> `.groups` argument.

dfPred1 <- dfAll[dfAll$Covariate==1,] %>% group_by(G,method,Codata,
  bam.method,Sim,Transform,Codata2) %>%
  summarise(MSE=mean(MSEridge),method2="ridge")
#> `summarise()` has grouped output by 'G', 'method', 'Codata', 'bam.method',
#> 'Sim', 'Transform'. You can override using the `.groups` argument.
dfPred2 <- dfAll[dfAll$Covariate==1,] %>% group_by(G,method,Codata,
  bam.method,Sim,Transform,Codata2) %>%
  summarise(MSE=mean(MSEecpc),method2="ecpc")
#> `summarise()` has grouped output by 'G', 'method', 'Codata', 'bam.method',
#> 'Sim', 'Transform'. You can override using the `.groups` argument.
dfPred <- rbind(dfPred1,dfPred2)

#Comparison different smoothing parameter methods
dfGAMs <- data.frame()
#gam
```

```
fname <- paste(pathResults,"SimResAppNoteGAM",".Rdata",sep="")
load(fname)
dfGAMs <- rbind(dfGAMs,df)
dfGAMs$G <- as.factor(dfGAMs$G)
dfGAMs$method <- factor(dfGAMs$method, levels=unique(dfGAMs$method),
                        labels=unique(dfGAMs$method))
dfGAMs$Codata <- factor(dfGAMs$Codata,levels=unique(dfGAMs$Codata),
                        labels=c("Random","Informative",
                                 "Random+transformed","Informative+transformed"))
dfGAMs$Codata2 <- factor(dfGAMs$Codata,levels=unique(dfGAMs$Codata),
                         labels=c("Random","Informative",
                                  "Random","Informative"))
#summarise estimated variance pointwise for continuous co-data per setting
dfEstGAMs <- dfGAMs %>% group_by(G,method,Codata,Covariate,bam.method, Z,
                                 ZUntransformed,Transform,Codata2) %>%
  summarise(meanVk = mean(vkfit),q50Vk = quantile(vkfit,0.5),
            q95Vk = quantile(vkfit,0.95),q05Vk = quantile(vkfit,0.05),
            q75Vk = quantile(vkfit,0.75),q25Vk = quantile(vkfit,0.25),
            truevk = mean(truevk)) %>% ungroup()
#> `summarise()` has grouped output by 'G', 'method', 'Codata', 'Covariate',
#> 'bam.method', 'Z', 'ZUntransformed', 'Transform'. You can override using the
#> `.groups` argument.

dfPred1GAMs <- dfGAMs[dfGAMs$Covariate==1,] %>% group_by(G,method,Codata,
  bam.method,Sim,Transform,Codata2) %>%
  summarise(MSE=mean(MSEridge),method2="ridge")
#> `summarise()` has grouped output by 'G', 'method', 'Codata', 'bam.method',
#> 'Sim', 'Transform'. You can override using the `.groups` argument.
dfPred2GAMs <- dfGAMs[dfGAMs$Covariate==1,] %>% group_by(G,method,Codata,
  bam.method,Sim,Transform,Codata2) %>%
  summarise(MSE=mean(MSEecpc),method2="ecpc")
#> `summarise()` has grouped output by 'G', 'method', 'Codata', 'bam.method',
#> 'Sim', 'Transform'. You can override using the `.groups` argument.
dfPredGAMs <- rbind(dfPred1GAMs,dfPred2GAMs)
```

Estimation and prediction performance for varying co-data models

The figure below shows prior variance estimates for different co-data models with corresponding prediction performance the next one below. As expected, the estimated prior variance is flat for random co-data and increasing for informative co-data, leading to prediction performance similar to and better than the co-data agnostic ordinary ridge, respectively. The estimates of the (constrained) generalised additive co-data models are slightly more non-linear than the linear estimate, but lead to similar prediction performance. The variance of the estimates of the constrained generalised additive models in the random co-data reflects the effect of adding constraints, e.g. the estimates vary mostly in the positive direction for the positively constrained model. The linear and (constrained) generalised additive model slightly outperform the adaptive discretisation.

```
#Figure estimated variance vs continuous co-data for all simulations,
#fixed G and fixed bam.method----
g <- G[1]
ggplot(dfEst[dfEst$G%in%c(1,2,g,7) & dfEst$Transform==F &
               dfEst$bam.method%in%c("ML","none","splits"),])+
  aes(x=Z)+
  #facet_wrap(Codata~method,scales="free_x",nrow = 2)+
  facet_grid(method~Codata,scales="free_x")+
```

```
geom_point(aes(x=Z,y=truevk),alpha=0.3,col="grey20")+
geom_ribbon(aes(ymin=q05Vk,ymax=q95Vk),linetype=0,alpha=0.1)+
geom_ribbon(aes(ymin=q25Vk,ymax=q75Vk),linetype=0,alpha=0.2)+
geom_line(aes(y=q50Vk),linewidth=1)+ #median
#geom_line(aes(y=meanVk),size=1)+ #mean
labs(y="Prior variance",x="Continuous co-data variable")+
theme_bw()+
theme(axis.text.x=element_text(size=ts),
      axis.text.y=element_text(size=ts),
      axis.title.x=element_text(size=ts+2),
      axis.title.y=element_text(size=ts+2),
      legend.text=element_text(size=ts),
      legend.title=element_text(size=ts+2),
      strip.text=element_text(size=ts))#,
```

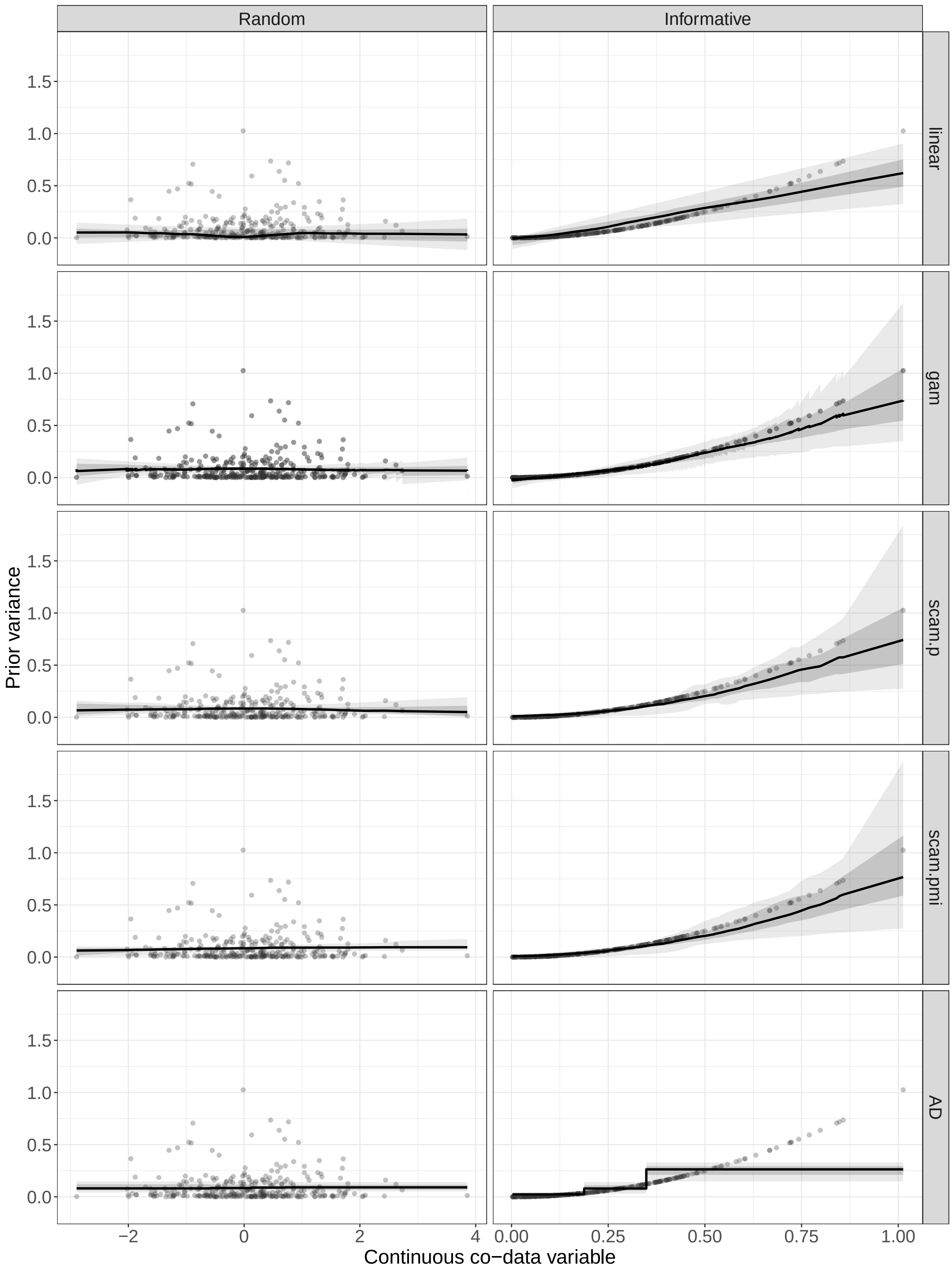
Random
Informative
linear
gam
scam.p
scam.pmi
AD
Prior variance
0.0
0.5
1.0
1.5
−2
0
2
4
0.00
0.25
0.50
0.75
1.00
Continuous co−data variable

```
#Figure boxplot prediction performance vs co-data setting for all simulations,
#fixed G and fixed bam.method----
g <- G[1]
temp <- dfPred[dfPred$G%in%c(1,2) & dfPred$bam.method%in%c("none") &
                 dfPred$method2=="ridge" ,]
temp$method <- "ridge"
temp$G <- factor(1,levels=c(1,2,7,20,50),labels=c(1,2,7,20,50))
temp2 <- dfPred[dfPred$G%in%c(1,2,g,7) &
                  dfPred$bam.method%in%c("none","splits","ML") &
                  dfPred$method2=="ecpc" ,]
temp2$G <- factor(temp2$G,levels=c(1,2,7,20,50),labels=c(1,2,7,20,50))
temp2 <- rbind(temp,temp2)
temp2$method <- factor(temp2$method, levels=unique(temp2$method)[c(1,2,4,5,6,3)],
                       labels=unique(temp2$method)[c(1,2,4,5,6,3)])

ggplot(temp2[temp2$Transform==F,])+#& !(dfPred$Codata=="noninformative"),])+
  aes(x=method,y=MSE)+
  geom_boxplot(fill="grey80")+
  facet_grid(.~Codata2)+
  #coord_cartesian(ylim=c(8,32))+
  #scale_y_log10()+
  labs(y="MSE",x="Co-data model")+
  theme_bw()+
  theme(axis.text.x=element_text(size=ts,angle=30,vjust=1,hjust=1),
        axis.text.y=element_text(size=ts),
        axis.title.x=element_text(size=ts+2),
        axis.title.y=element_text(size=ts+2),
        legend.text=element_text(size=ts),
        legend.title=element_text(size=ts+2),
        strip.text=element_text(size=ts))#,
```

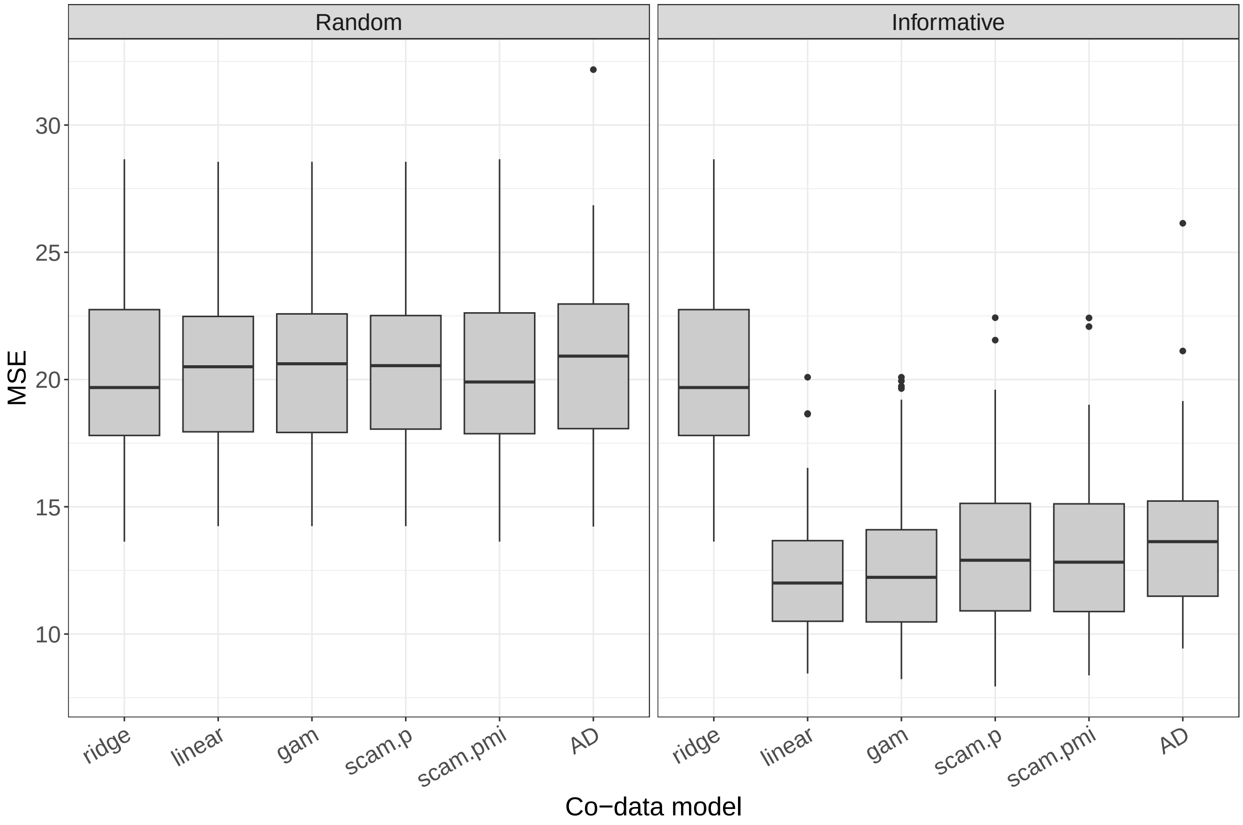

Estimation and performance for different smoothing penalty estimation methods

One advantage of the additive models using p-splines over the adaptive discretisation is that the p-splines can estimate local changes on a finer level; while the adaptive discretisation method is limited to discretisations in which each discretised group contains at least one variable (in our case, at least 20 variables per discretised group), this is not needed for p-splines, as they are penalised with a difference penalty. To illustrate, the figures below shows the generalised additive model estimates in one training data set when $G = 20$ or $G = 50$ splines are used and when the difference penalty is estimated with one of the methods provided in the R-function `bam()` or with the random splits as used in the first version of ecpc. Except for the generalised cross-validation criterion `GCV.Cp`, the estimates and corresponding prediction performance seem to be robust for the number of splines.

```
#Figure estimated variance vs continuous co-data for 1 run, different bam.methods----
g <- G[1]
Sim <- 1
ggplot(dfGAMs[dfGAMs$Sim==Sim & dfGAMs$Transform==F & dfGAMs$G%in%c(1,G) ,])+
  aes(x=Z,col=bam.method)+
  facet_grid(.~Codata,scales="free_x")+
  geom_point(aes(x=Z,y=truevk),alpha=0.1,col="black")+
  geom_line(aes(y=vkfit,linetype=G),linewidth=1.5)+
  labs(y="Prior variance",x="Continuous co-data variable")+
  scale_color_manual(values=colsAUC[1:4])+
  theme_bw()+
  theme(axis.text.x=element_text(size=ts),
        axis.text.y=element_text(size=ts),
        axis.title.x=element_text(size=ts+2),
        axis.title.y=element_text(size=ts+2),
```

```
        legend.text=element_text(size=ts),
        legend.title=element_text(size=ts+2),
        strip.text=element_text(size=ts))#,
```

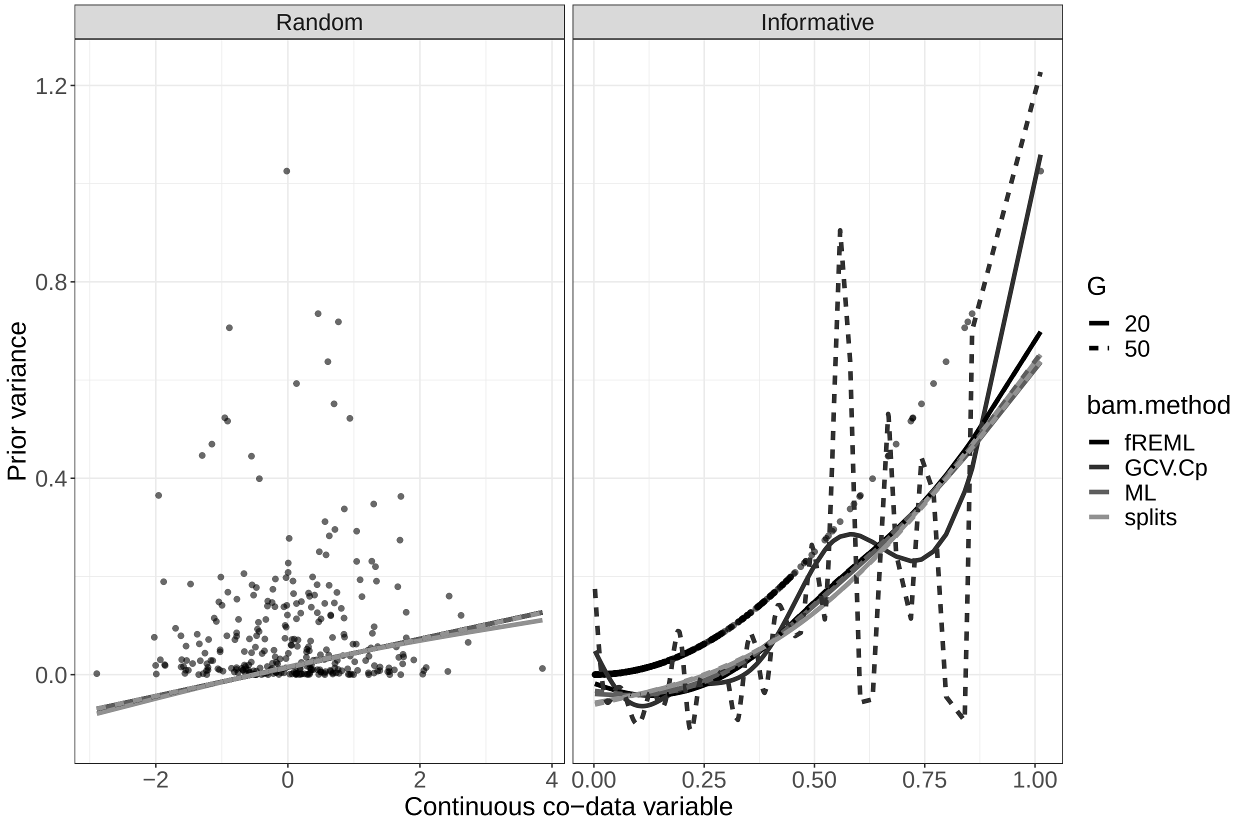


```
#Figure estimated variance vs continuous co-data for all simulations,
#gam only, fixed G and different bam.method----
g <- G[1]
ggplot(dfEstGAMs[dfEstGAMs$G%in%c(1,2,g) & dfEstGAMs$Transform==F ,])+
  aes(x=Z)+
  facet_grid(bam.method~Codata,scales="free_x")+
  geom_point(aes(x=Z,y=truevk),alpha=0.3,col="black")+
  geom_ribbon(aes(ymin=q05Vk,ymax=q95Vk),linetype=0,alpha=0.1)+
  geom_ribbon(aes(ymin=q25Vk,ymax=q75Vk),linetype=0,alpha=0.2)+
  geom_line(aes(y=q50Vk),linewidth=1)+ #median
  labs(y="Prior variance",x="Continuous co-data variable")+
  theme_bw()+
  theme(axis.text.x=element_text(size=ts),
        axis.text.y=element_text(size=ts),
        axis.title.x=element_text(size=ts+2),
        axis.title.y=element_text(size=ts+2),
        legend.text=element_text(size=ts),
        legend.title=element_text(size=ts+2),
        strip.text=element_text(size=ts))#,
```

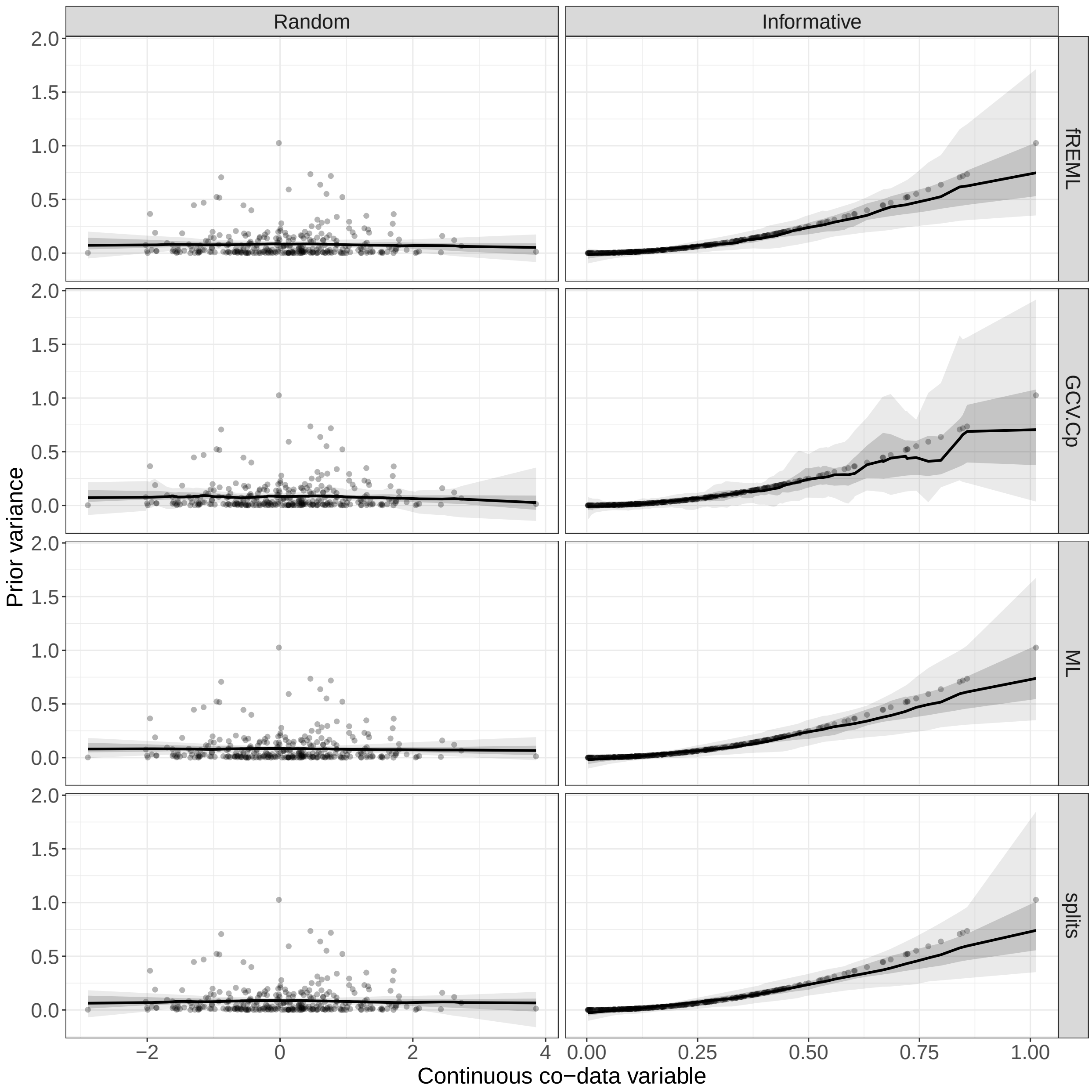


```
#Figure boxplot prediction performance vs co-data setting for all simulations,
#gam only, fixed G and different bam.method----
g <- G[1]
temp <- dfPred[dfPred$G%in%c(1,2) & dfPred$bam.method%in%c("none") &
                 dfPred$method2=="ridge" ,]
temp$method <- "ridge"
temp$G <- factor(1,levels=c(1,20,50),labels=c(1,20,50))
temp2 <- dfPredGAMs[dfPredGAMs$G%in%c(G) & dfPredGAMs$method%in%c("gam") &
                      dfPredGAMs$method2=="ecpc" ,]
temp2$G <- factor(temp2$G,levels=c(1,20,50),labels=c(1,20,50))
temp2 <- rbind(temp,temp2)
temp2$bam.method <- factor(temp2$bam.method, levels=unique(temp2$bam.method)[c(1,4,2,3,5)],
                           labels=c("ridge",unique(temp2$bam.method)[c(4,2,3,5)]))
```

```
ggplot(temp2[temp2$Transform==F,])+#& !(dfPred$Codata=="noninformative"),])+
  aes(x=bam.method,y=MSE)+
  geom_boxplot(aes(fill=G))+
  facet_grid(.~Codata2)+
  #scale_y_log10()+-
  labs(y="MSE",x="")+
  scale_fill_manual(values=rev(rev(colsAUC)[1:3]))+
  theme_bw()+
  theme(axis.text.x=element_text(size=ts,angle=30,vjust=1,hjust=1),
        axis.text.y=element_text(size=ts),
        axis.title.x=element_text(size=ts+2),
        axis.title.y=element_text(size=ts+2),
        legend.text=element_text(size=ts),
        legend.title=element_text(size=ts+2),
        strip.text=element_text(size=ts))#,
```

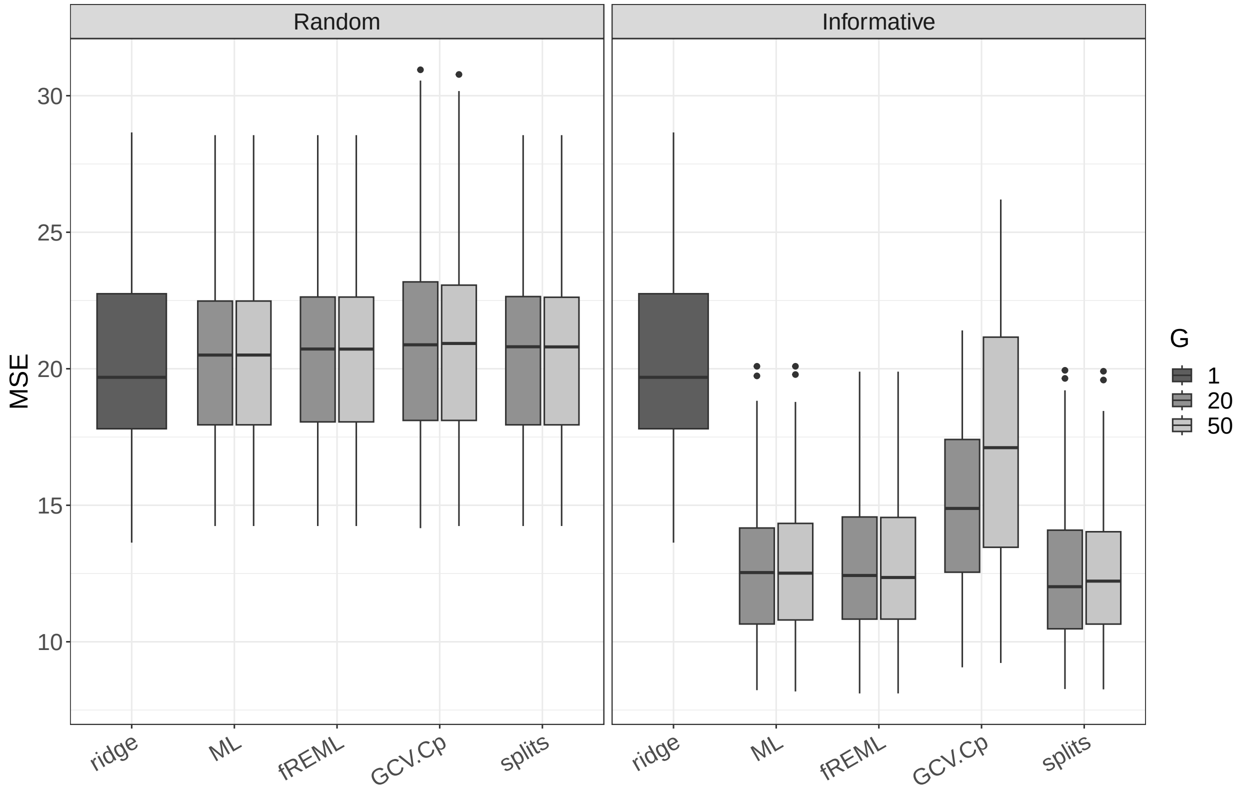

Table computation time

Finally, the table below shows the average run times of the methods. The adaptive discretisation is around 3-6 times as slow as the (constrained) additive co-data models.

```
#Table average computing times + standard deviation----
timeTable <- dfAll[dfAll$Covariate==1,] %>% group_by(method,bam.method,G) %>%
  summarise(meanTime=mean(Time),sdTime=sd(Time)) %>% ungroup()
#> `summarise()` has grouped output by 'method', 'bam.method'. You can override
#> using the `.groups` argument.
print(timeTable)
```

```
#> # A tibble: 14 x 5
#>    method    bam.method G     meanTime sdTime
#>    <fct>     <chr>      <fct>    <dbl>  <dbl>
#>  1 linear    none       2         12.7   4.54
#>  2 gam       fREML      20        12.3   5.57
#>  3 gam       fREML      50        13.0   5.61
#>  4 gam       GCV.Cp     20        11.6   5.49
#>  5 gam       GCV.Cp     50        12.1   5.81
#>  6 gam       ML         20        12.5   5.59
#>  7 gam       ML         50        12.9   5.58
#>  8 gam       splits     20        13.2   5.50
#>  9 gam       splits     50        16.5   5.76
#> 10 scam.p    splits     20        16.5  11.5
#> 11 scam.p    splits     50        19.8  10.6
#> 12 scam.pmi  splits     20        20.1  17.6
#> 13 scam.pmi  splits     50        26.2  20.4
#> 14 AD        splits     7         72.1   9.77
#library("writexl")
#fname <- paste(pathFigures,"TimeTableRaw.xlsx")
#write_xlsx(timeTable,fname)
```

Simulation study 2: Variable selection compared to other methods

We alter the simulation set-up from above for variable selection. We now set 250 regression coefficients to 0, leaving 50 non-zero coefficients. We scale the regression coefficients such that the L2-norm of β^0 and the scaled, sparse $\beta^{0,s}$ are the same. We use the following co-data:

1. Random: as in the simulation study above, so $Z_k^{(1)} \overset{i.i.d.}{\sim} N(0,1)$ for $k = 1,..,p$.
2. Informative+monotone: as in the simulation study above, but with white noise added such that the co-data is not exactly 0 for the zero coefficients, $Z_k^{(2)} \overset{ind.}{\sim} N(|\beta_k^{0,s}|, \sigma_0^2)$, for σ_0 a tenth of the sample standard deviation of $\beta^{0,s}$. The effect size $|\beta_k^{0,s}|^2$ is (up till some noise) a monotone, quadratic function of the co-data.
3. Informative+convex: similar to the Informative co-data but distinguishing negative from positive effects by $Z_k^{(3)} = sign(\beta_k^{0,s}) \cdot Z_k^{(2)}$. The effect size $|\beta_k|^2$ is now (up till some noise) not monotone but a convex, quadratic function of the co-data.

We compare the following variable selection methods:

- glmnet: a co-data agnostic elastic net model fitted with glmnet [**?**].
- fwelnet: an elastic net model with continuous co-data, fitted with fwelnet [**?**]. The elastic net penalties are a fixed, exponential function of the co-data weights.
- ecpc+squeezy: a GAM for the co-data fitted with ecpc, followed by a transformation of the ridge penalties to elastic net penalties by squeezy [**?**] for variable selection.
- ecpc+postselection: a GAM for the co-data, using the default option for posterior selection provided in the ecpc software.

The first three methods have one additional tuning parameter, the elastic net parameter $\alpha \in [0, 1]$, with 0 corresponding to the full model and 1 to the lasso model. The last method has one tuning parameter, the number of selected covariates (or equivalently, the proportion of estimated zero effects), ranging from 300 to 0 (0 to 1) for the full model to the most sparse model. In practice, one may choose one value from a range of values for the tuning parameter by comparing predictive performances and selecting the sparsest model that performs (nearly) optimal.

Run analysis

To rerun the analysis and generation of toy data, first set the following variables to `TRUE`:

```
run_ecpc <- FALSE
run_glmnet <- FALSE
run_fwelnet <- FALSE

generateData <- TRUE #set to true if not generated before
nSim <- 50 #set to lower number for quicker run
runParallel <- FALSE
```

Load libraries

Load the libraries needed for the analysis. Use multiple cores if available and if `runParallel=TRUE`.

```
#Load libraries----
library(ecpc)
library(squeezy)
library(glmnet)
#> Loading required package: Matrix
#> Loaded glmnet 4.1-4
library(fwelnet)
library(dplyr) #for data wrangling results
library(ggplot2) #for plotting results
library(RColorBrewer) #for plotting results
library(foreach) #for parallel computing
library(doParallel) #for parallel computing
library(mvtnorm)


#optional:
if(runParallel){ #set to 1 to setup parallel backend to use many processors
  cores=detectCores()
  if(!("cl"%in%ls())){
    cl <- makeCluster(cores-1) #not to overload your computer
    registerDoParallel(cl)
  }
}

setting_q <- 1
q <- 5/6 #proportion zeros
maxsel <- c(rep(2:10),10*2:29)
alp_range <- seq(0,1,length.out = length(maxsel))
```

Generate data

Simulate multiple training and test data sets.

```
#Simulate toy data ------
p<-300 #number of covariates
n<-100 #sample size training data set
n2<-100 #sample size test data set
sigma <- 1
tauglobal <- 0.1 #prior variance; 0.1, 0.5
model <- "linear"; fam <- "gaussian"
```

```r
rho <- 0 #correlation in observed X matrix
Sigma <- matrix(rho,p,p); diag(Sigma) <- 1

#simulate all betas i.i.d. from beta_k~N(mean=0,sd=sqrt(0.1)):
set.seed(7474)
Dat <- list()
Dat$beta <- rnorm(p,sd=sqrt(tauglobal))
ind0 <- sample(1:p,floor(q*p),replace=F)
Dat$beta[ind0] <- 0
Dat$beta <- Dat$beta/sqrt(sum(Dat$beta^2))*sqrt(tauglobal*p)
#Dat$Xctd <- matrix(rnorm(n*p) ,n,p)
#Dat$X2ctd <- matrix(rnorm(n2*p) ,n2,p)
Dat$Xctd <- rmvnorm(n,mean=rep(0,p),sigma=Sigma)
Dat$X2ctd <- rmvnorm(n2,mean=rep(0,p),sigma=Sigma)
Dat$lp2 <- Dat$X2ctd%*%Dat$beta
#hist(Dat$lp2)
Dat$Y <- rnorm(n,mean=c(Dat$Xctd%*%Dat$beta),sd=sigma)

#index true, non-zero betas
ind_nonzero <- which(Dat$beta!=0)
ind_zero <- which(Dat$beta==0)

if(generateData){
  AllData <- list()
  for(i in 1:nSim){
    AllData[[i]] <- list()
    AllData[[i]]$beta <- Dat$beta
    if(rho==0){
      AllData[[i]]$Xctd <- matrix(rnorm(n*p) ,n,p)
      AllData[[i]]$X2ctd <- matrix(rnorm(n2*p) ,n2,p)
    }else{
      AllData[[i]]$Xctd <- rmvnorm(n,mean=rep(0,p),sigma=Sigma)
      AllData[[i]]$X2ctd <- rmvnorm(n2,mean=rep(0,p),sigma=Sigma)
    }
    means <- apply(AllData[[i]]$Xctd,2,mean)
    sds <- apply(AllData[[i]]$Xctd,2,sd)

    AllData[[i]]$Y <- rnorm(n,mean=c(AllData[[i]]$Xctd%*%AllData[[i]]$beta),sd=sigma)
    AllData[[i]]$Y2 <- rnorm(n2,mean=c(AllData[[i]]$X2ctd%*%AllData[[i]]$beta),sd=sigma)
  }
  save(Dat,AllData,file=paste(pathResults,"SimDataSparse",setting_q,rho,".Rdata",sep=""))
}else{
  load(paste(pathResults,"SimDataSparse",setting_q,rho,".Rdata",sep=""))
}
```

Configure co-data

```r
#Co-data settings----
G <- c(20,50) #number of splines
Z.all <- list()
ZI.all <- list() #co-data matrix with intercept
Zs.all <- list()
S1 <- list()
```

```
Con.p <- list() #positivity constraints
Con.pmi <- list() #positivity and monotonically increasing constraints
#setting 1: non-informative, unequally spaced
Z.all[["noninformative"]] <- rnorm(p) #for linear co-data model
ZI.all[["noninformative"]] <- cbind(rep(1,p), Z.all[["noninformative"]])
#for generalised additive co-data model:
for(g in 1:length(G)){
  Zs.all[[g]] <- list()
  Zs.all[[g]][["noninformative"]] <- createZforSplines(
    values=Z.all[["noninformative"]], G=G[g], bdeg=3)
  #create 2nd order difference penalty matrix (same for all co-data)
  S1[[g]] <- createS(orderPen=2, G=G[g])

  Con.p[[g]] <- createCon(G=G[g], shape="positive") #create constraints
  Con.pmi[[g]] <- createCon(G=G[g], shape="positive+monotone.i") #create constraints
}
#plot(Z.all[[1]],Dat$beta^2)

#setting 2: informative, unequally spaced, information at edge
set.seed(101010)
Z.all[["size.edge"]] <- abs(Dat$beta)+rnorm(p,sd=sd(Dat$beta)/10)
ZI.all[["size.edge"]] <- cbind(rep(1,p), Z.all[["size.edge"]])
#for generalised additive co-data model:
for(g in 1:length(G)){
  Zs.all[[g]][["size.edge"]] <- createZforSplines(values=Z.all[["size.edge"]],
                                                   G=G[g], bdeg=3)
}
#plot(Z.all[[2]],Dat$beta^2)

#setting 3: informative, unequally spaced, information at 2 edges
set.seed(101010)
Z.all[["size.edge2"]] <- sign(Dat$beta)*abs(Dat$beta)+rnorm(p,sd=sd(Dat$beta)/10)
ZI.all[["size.edge2"]] <- cbind(rep(1,p), Z.all[["size.edge2"]])
#for generalised additive co-data model:
for(g in 1:length(G)){
  Zs.all[[g]][["size.edge2"]] <- createZforSplines(values=Z.all[["size.edge2"]],
                                                    G=G[g], bdeg=3)
}
#plot(Z.all[[3]],Dat$beta^2)

#fwelnet
Z_fwelnet <- list(matrix(Z.all[["noninformative"]],nrow = p),
                  matrix(Z.all[["size.edge"]],nrow = p),
                  matrix(Z.all[["size.edge2"]],nrow = p))
```

ecpc+posterior selection

```
#Fit ecpc+posterior selection on simulated data sets: generalised additive co-data model----
fname <- paste(pathResults,"SimResAppNoteSparse_GAM",setting_q,rho,".Rdata",sep="")
print(fname)

if(run_ecpc){
  #df <- data.frame()
```

```
#for(sim in 1:nSim){
finalMatrix <- foreach(sim=1:nSim, .combine=rbind,
                       .packages = c("ecpc", "squeezy")) %dopar% {
           df <- data.frame()
           dfSelect <- data.frame()
           for(setting in 1:3){
             for(g in 1){
               for(method in c("ML")){
                 tic<-proc.time()[[3]]

                 if(method=="splits"){
                   fit <- ecpc(Y=AllData[[sim]]$Y,X=AllData[[sim]]$Xctd,
                               Z=Zs.all[[g]][setting],
                               paraPen=list(Z1=list(S1=S1[[g]])),
                               intrcpt.bam = F,
                               model=model,maxsel=maxsel,
                               Y2=AllData[[sim]]$Y2,X2=AllData[[sim]]$X2ctd,
                               hypershrinkage = "ridge",
                               est_beta_method = "multiridge")
                 }else{
                   fit <- ecpc(Y=AllData[[sim]]$Y,X=AllData[[sim]]$Xctd,
                               Z=Zs.all[[g]][setting],
                               paraPen=list(Z1=list(S1=S1[[g]])),
                               bam.method=method,intrcpt.bam = F,
                               model=model,maxsel=maxsel,
                               Y2=AllData[[sim]]$Y2,X2=AllData[[sim]]$X2ctd,
                               est_beta_method = "multiridge")
                 }


                 toc <- proc.time()[[3]]-tic

                 vk <- (Zs.all[[g]][[setting]]%*%fit$gamma )*fit$tauglobal
                 #plot(Z.all[[setting]],vk)

                 temp<-data.frame(vkfit=vk,truebeta=Dat$beta,
                                  truevk=Dat$beta^2)
                 temp$Z <- Z.all[[setting]]
                 temp$Time <- toc
                 temp$Covariate <- 1:p
                 temp$G <- G[g]
                 temp$setting <- setting
                 temp$bam.method <- method
                 temp$method <- "gam"
                 temp$MSEridge <- fit$MSEridge
                 temp$MSEecpc <- fit$MSEecpc
                 temp$Sim <- sim
                 temp$Codata <- names(Z.all)[setting]
                 temp$Transform <- F; if(setting>=3) temp$Transform <- T
                 temp$ZUntransformed <- Z.all[[setting]]
                 temp$q <- q
                 temp$rho <- rho
                 if(setting>=3) temp$ZUntransformed <- Z.all[[setting-2]]
```

```
df <- rbind(df,temp)

#sensitivity = true positive rate = true positives/total positives
sensitivity <- apply(fit$betaPost,2,function(x){
  ind_nonzero_est <- which(x!=0)
  TPR <-sum(ind_nonzero_est%in%ind_nonzero)/length(ind_nonzero)
  return(TPR)
})
#precision = true negative rate = true negatives/total negatives
precision <- apply(fit$betaPost,2,function(x){
  ind_zero_est <- which(x==0)
  TNR <-sum(ind_zero_est%in%ind_zero)/length(ind_zero)
  return(TNR)
})
lp_train <- AllData[[sim]]$Xctd%*%fit$betaPost
MSEtrain <- apply(lp_train,2,function(lp)
  mean((lp-AllData[[sim]]$Y)^2))

temp<-data.frame("Tuningparam"=maxsel, sensitivity, precision)
temp$TypeTuning <- "#params"
temp$Time <- toc
temp$G <- G[g]
temp$setting <- setting
temp$bam.method <- method
temp$method <- "gam"
temp$MSEridge <- fit$MSEridge
temp$MSEecpc <- c(fit$MSEPost)
temp$MSEtrain <- c(MSEtrain)
if(is.null(fit$MSEPost)) temp$MSEecpc <- rep(NaN,length(maxsel))
temp$Sim <- sim
temp$Codata <- names(Z.all)[setting]
temp$Transform <- F; if(setting>=3) temp$Transform <- T
temp$q <- q
temp$setting_q <- setting_q
temp$rho <- rho
dfSelect <- rbind(dfSelect, temp)

#fit squeezy
#Use squeezy function to transform estimated ridge penalties to elastic net
#penalties
notinf <- fit$penalties!=Inf
fit.EN <- lapply(alp_range,function(alp){
  fit.EN <- squeezy(Y=AllData[[sim]]$Y,
                    X=AllData[[sim]]$Xctd[,notinf],
                    groupset=lapply(1:sum(notinf),
                                    function(x) x),
                    alpha=alp,
                    Y2=AllData[[sim]]$Y2,
                    X2=AllData[[sim]]$X2ctd[,notinf],
                    lambdas=fit$penalties[notinf],
                    sigmasq=fit$sigmahat)})
beta_squeezy <- sapply(1:length(alp_range),function(i){
  betas <- rep(0,p)
```

```
                      betas[notinf] <- fit.EN[[i]]$betaApprox
                      return(betas)
                    })
                    MSE_squeezy <- sapply(1:length(alp_range),function(i){
                      fit.EN[[i]]$MSEApprox
                    })
                    lp_train <- AllData[[sim]]$Xctd%*%beta_squeezy
                    MSEtrain <- apply(lp_train,2,function(lp)
                      mean((lp-AllData[[sim]]$Y)^2))

                    #sensitivity = true positive rate =
                    #true positives/total positives
                    sensitivity <- apply(beta_squeezy,2,function(x){
                      ind_nonzero_est <- which(x!=0)
                      TPR <-sum(ind_nonzero_est%in%ind_nonzero)/length(ind_nonzero)
                      return(TPR)
                    })
                    #precision = true negative rate =
                    #true negatives/total negatives
                    precision <- apply(beta_squeezy,2,function(x){
                      ind_zero_est <- which(x==0)
                      TNR <-sum(ind_zero_est%in%ind_zero)/length(ind_zero)
                      return(TNR)
                    })

                    temp<-data.frame("Tuningparam"=alp_range, sensitivity, precision)
                    temp$TypeTuning <- "alpha"
                    temp$Time <- toc
                    temp$G <- G[g]
                    temp$setting <- setting
                    temp$bam.method <- method
                    temp$method <- "gam"
                    temp$MSEridge <- fit$MSEridge
                    temp$MSEecpc <- MSE_squeezy
                    temp$MSEtrain <- c(MSEtrain)
                    temp$Sim <- sim
                    temp$Codata <- names(Z.all)[setting]
                    temp$Transform <- F; if(setting>=3) temp$Transform <- T
                    temp$q <- q
                    temp$setting_q <- setting_q
                    temp$rho <- rho
                    dfSelect <- rbind(dfSelect,temp)

                  }
                }
              }
              list("df"=df,"dfSelect"=dfSelect)
            }
#str(finalMatrix)
df2 <- lapply(1:nSim,function(i) finalMatrix[i,1][[1]])
dfSelect2 <- lapply(1:nSim,function(i) finalMatrix[i,2][[1]])
df <- df2[[1]]; for(i in 2:nSim) df <- rbind(df,df2[[i]])
dfSelect <- dfSelect2[[1]]; for(i in 2:nSim) dfSelect <- rbind(dfSelect,dfSelect2[[i]])
```

```
  save(finalMatrix,df,dfSelect,file=fname)
}
```

glmnet

```
#Fit glmnet on simulated data sets----
fname <- paste(pathResults,"SimResAppNoteSparse_glmnet",setting_q,rho,".Rdata",sep="")
print(fname)

if(run_glmnet){
  #df <- data.frame()
  #for(sim in 1:nSim){
  finalMatrix <- foreach(sim=1:nSim, .combine=rbind,
                         .packages = c("glmnet")) %dopar% {
         dfSelect <- data.frame()
         for(setting in 1:3){
           for(alp in alp_range){
             tic<-proc.time()[[3]]

             fit.glmnet <- glmnet::cv.glmnet(y=AllData[[sim]]$Y,
                                             x=AllData[[sim]]$Xctd,
                                             family=fam,alpha=alp)
             beta.glmnet <- coef(fit.glmnet,s="lambda.min", exact=TRUE)
             Ypred.glmnet <- c(predict(fit.glmnet, newx = AllData[[sim]]$X2ctd,
                                       s = "lambda.min", type="response", exact=TRUE))
             MSE.glmnet <- mean((Ypred.glmnet-AllData[[sim]]$Y2)^2)
             lp_train <- AllData[[sim]]$Xctd%*%beta.glmnet[-1] + beta.glmnet[1]
             MSEtrain <- mean((lp_train-AllData[[sim]]$Y)^2)
             toc <- proc.time()[[3]]-tic

             #sensitivity = true positive rate = true positives/total positives
             ind_nonzero_est <- which(beta.glmnet[-1]!=0)
             sensitivity <- sum(ind_nonzero_est%in%ind_nonzero)/length(ind_nonzero)
             #precision = true negative rate = true negatives/total negatives
             ind_zero_est <- which(beta.glmnet[-1]==0)
             precision <- sum(ind_zero_est%in%ind_zero)/length(ind_zero)

             temp<-data.frame("Tuningparam"=alp, sensitivity, precision)
             temp$TypeTuning <- "alpha"
             temp$Time <- toc
             temp$G <- 1
             temp$setting <- setting
             temp$bam.method <- "none"
             temp$method <- "glmnet"
             temp$MSEridge <- NaN
             temp$MSEecpc <- MSE.glmnet
             temp$MSEtrain <- MSEtrain
             temp$Sim <- sim
             temp$Codata <- names(Z.all)[setting]
             temp$Transform <- F; if(setting>=3) temp$Transform <- T
             temp$q <- q
             temp$setting_q <- setting_q
             temp$rho <- rho
```

```
            dfSelect <- rbind(dfSelect,temp)

          }
        }
        list("dfSelect"=dfSelect)
      }
  #str(finalMatrix)
  dfSelect <- finalMatrix[[1]]; for(i in 2:nSim) dfSelect <- rbind(dfSelect,finalMatrix[[i]])
  save(finalMatrix,dfSelect,file=fname)
}
```

fwelnet

```
#Fit fwelnet on simulated data sets----
fname <- paste(pathResults,"SimResAppNoteSparse_fwelnet",setting_q,rho,".Rdata",sep="")
print(fname)

if(run_fwelnet){
  #df <- data.frame()
  #for(sim in 1:nSim){
  finalMatrix <- foreach(sim=1:nSim, .combine=rbind,
                         .packages = c("fwelnet")) %dopar% {
        dfSelect <- data.frame()
        for(setting in 1:3){
          for(alp in alp_range){
            tic<-proc.time()[[3]]

            res.fwEN <-  cv.fwelnet(y=AllData[[sim]]$Y,x=AllData[[sim]]$Xctd,
                                   z=Z_fwelnet[[setting]],
                                   alpha=alp,family=fam,standardize=F)

            ind.minlam <- which(res.fwEN$lambda==res.fwEN$lambda.min)

            betafwEN <- res.fwEN$glmfit$beta[,ind.minlam]
            a0fwEN <- res.fwEN$glmfit$a0[ind.minlam]
            Ypred_fwelnet <- AllData[[sim]]$X2ctd%*%betafwEN+a0fwEN
            MSE_fwelnet <- mean((Ypred_fwelnet-AllData[[sim]]$Y2)^2)
            lp_train <- AllData[[sim]]$Xctd%*%betafwEN + a0fwEN
            MSEtrain <- mean((lp_train-AllData[[sim]]$Y)^2)

            toc <- proc.time()[[3]]-tic

            #sensitivity = true positive rate = true positives/total positives
            ind_nonzero_est <- which(betafwEN!=0)
            sensitivity <- sum(ind_nonzero_est%in%ind_nonzero)/length(ind_nonzero)
            #precision = true negative rate = true negatives/total negatives
            ind_zero_est <- which(betafwEN==0)
            precision <- sum(ind_zero_est%in%ind_zero)/length(ind_zero)

            temp<-data.frame("Tuningparam"=alp, sensitivity, precision)
            temp$TypeTuning <- "alpha"
            temp$Time <- toc
            temp$G <- 1
```

```
            temp$setting <- setting
            temp$bam.method <- "none"
            temp$method <- "fwelnet"
            temp$MSEridge <- NaN
            temp$MSEecpc <- MSE_fwelnet
            temp$MSEtrain <- c(MSEtrain)
            temp$Sim <- sim
            temp$Codata <- names(Z.all)[setting]
            temp$Transform <- F; if(setting>=3) temp$Transform <- T
            temp$q <- q
            temp$setting_q <- setting_q
            temp$rho <- rho
            dfSelect <- rbind(dfSelect,temp)

          }
        }
        list("dfSelect"=dfSelect)
      }
  #str(finalMatrix)
  dfSelect <- finalMatrix[[1]]; for(i in 2:nSim) dfSelect <- rbind(dfSelect,finalMatrix[[i]])
  save(finalMatrix,dfSelect,file=fname)
}
```

Results

Load results

First, set some plotting parameters and load the pre-saved results:

```
#Plots: general parameters----
wdth<-600
hght<-wdth*5/8
wdthpdf <- wdth/75
hghtpdf <- hght/75
ts <- 16 #basis text size in figures
ls <- 1.5 #basis line size in figures
ps <- 2 #basis point size in figures
sz <- 2 #point size
strk <- 1.5 #stroke size
palette <- "Dark2"
#display.brewer.all(10,colorblindFriendly=T)
colpal <- "Dark2"
colsfill <- brewer.pal(3,"Dark2")[1:2]
colsAUC <- brewer.pal(10,"RdYlBu")

#Load data for plots----
#All estimates and predictions
dfAll <- data.frame()
#gam
fname <- paste(pathResults,"SimResAppNoteSparse_GAM",
               setting_q,rho,".Rdata",sep="")
load(fname)
dfSelect$method[dfSelect$TypeTuning=="#params"] <- "ecpc+postselection"
dfSelect$method[dfSelect$TypeTuning=="alpha"] <- "ecpc+squeezy"
```

```
dfAll <- rbind(dfAll,dfSelect)
#glmnet
fname <- paste(pathResults,"SimResAppNoteSparse_glmnet",
               setting_q,rho,".Rdata",sep="")
load(fname)
dfAll <- rbind(dfAll,dfSelect)

#fwelnet
fname <- paste(pathResults,"SimResAppNoteSparse_fwelnet",
               setting_q,rho,".Rdata",sep="")
load(fname)
dfAll <- rbind(dfAll,dfSelect)

dfAll$Tuningparam2 <- dfAll$Tuningparam
dfAll$TypeTuning2 <- dfAll$TypeTuning
dfAll$Tuningparam2[dfAll$TypeTuning=="#params"] <-
  (p-dfAll$Tuningparam2[dfAll$TypeTuning=="#params"])/p
dfAll$TypeTuning2[dfAll$TypeTuning=="#params"] <- "proportion zeros"

dfAll$G <- as.factor(dfAll$G)
dfAll$method <- factor(dfAll$method, levels=unique(dfAll$method)[c(3,4,2,1)],
                       labels=unique(dfAll$method)[c(3,4,2,1)])
dfAll$Codata <- factor(dfAll$Codata,levels=unique(dfAll$Codata),
                       labels=c("Random","Informative+monotone","Informative+convex"))

dfPred <- dfAll %>% group_by(method,Codata,TypeTuning,Tuningparam,TypeTuning2,Tuningparam2) %>%
  summarise(meanMSE=mean(MSEecpc,na.rm=TRUE),method2="EN",
            q50MSE = quantile(MSEecpc,0.5,na.rm=TRUE),
            q95MSE = quantile(MSEecpc,0.95,na.rm=TRUE),
            q05MSE = quantile(MSEecpc,0.05,na.rm=TRUE),
            q75MSE = quantile(MSEecpc,0.75,na.rm=TRUE),
            q25MSE = quantile(MSEecpc,0.25,na.rm=TRUE),
            meanMSEtrain=mean(MSEtrain,na.rm=TRUE),method2="EN",
            q50MSEtrain = quantile(MSEtrain,0.5,na.rm=TRUE),
            q95MSEtrain = quantile(MSEtrain,0.95,na.rm=TRUE),
            q05MSEtrain = quantile(MSEtrain,0.05,na.rm=TRUE),
            q75MSEtrain = quantile(MSEtrain,0.75,na.rm=TRUE),
            q25MSEtrain = quantile(MSEtrain,0.25,na.rm=TRUE),
            meansensitivity=mean(sensitivity,na.rm=TRUE),
            q50sens = quantile(sensitivity,0.5,na.rm=T),
            q95sens = quantile(sensitivity,0.95,na.rm=T),
            q05sens = quantile(sensitivity,0.05,na.rm=T),
            q75sens = quantile(sensitivity,0.75,na.rm=T),
            q25sens = quantile(sensitivity,0.25,na.rm=T),
            meanprecision=mean(precision,na.rm=TRUE),
            q50prec = quantile(precision,0.5,na.rm=T),
            q95prec = quantile(precision,0.95,na.rm=T),
            q05prec = quantile(precision,0.05,na.rm=T),
            q75prec = quantile(precision,0.75,na.rm=T),
            q25prec = quantile(precision,0.25,na.rm=T)) %>% ungroup()
#> `summarise()` has grouped output by 'method', 'Codata', 'TypeTuning',
#> 'Tuningparam', 'TypeTuning2'. You can override using the `.groups` argument.
```

Prediction and variable selection performance

The figures below show the performance of the methods in variable selection and prediction error on the test data. Note that ecpc+postselection may be tuned to select sparse models up to a model that is almost empty, reaching a sensitivity and 1-precision of 0. In contrast, the models selected by the other methods still contain more variables, even in the most sparse models for tuning parameter $\alpha = 1$. Besides, ecpc+squeezy and ecpc+postselection do not always select a full model, explaining why the sensitivities do not reach 1. This is a result from `ecpc()` truncating estimated negative prior variances to 0, deselecting some variables a priori. In the sparse setting, the co-data agnostic glmnet outperforms the other methods both in terms of variable selection and prediction performance when the co-data is random. This in contrast to the dense simulation setting, in which the prediction performance of glmnet and gam were on par. For the informative+monotone co-data, fwelnet slightly outperforms ecpc+squeezy and ecpc+postselection, all outperforming glmnet. For the informative+convex co-data, however, fwelnet is not able to flexibly adapt to the convex shape of the co-data, while the flexible GAM for the co-data in ecpc+squeezy and ecpc+postselection still adequately exploits the co-data.

```
#Figure MSE for all simulations, tuning parameter 2----
ggplot(dfPred)+# & dfEst$method=="ML",])+
  aes(x=Tuningparam2, col=method, fill=method)+
  facet_grid(.~Codata)+
  geom_ribbon(aes(ymin=q05MSE,ymax=q95MSE),linetype=0,alpha=0.1)+
  geom_ribbon(aes(ymin=q25MSE,ymax=q75MSE),linetype=0,alpha=0.2)+
  #geom_line(aes(y=q50MSE, linetype=TypeTuning2),size=1)+ #median
  geom_line(aes(y=meanMSE, linetype=TypeTuning2),size=1)+ #mean
  #scale_y_log10()+
  labs(y="MSE",x="Tuning parameter")+
  guides(fill=guide_legend(title="Method"),
         color=guide_legend(title="Method"),
         linetype=guide_legend(title="Type tuning parameter"))+
  theme_bw()+
  theme(axis.text.x=element_text(size=ts),
        axis.text.y=element_text(size=ts),
        axis.title.x=element_text(size=ts+2),
        axis.title.y=element_text(size=ts+2),
        legend.text=element_text(size=ts),
        legend.title=element_text(size=ts+2),
        strip.text=element_text(size=ts))#,
#> Warning: Using `size` aesthetic for lines was deprecated in ggplot2 3.4.0.
#> i Please use `linewidth` instead.
```

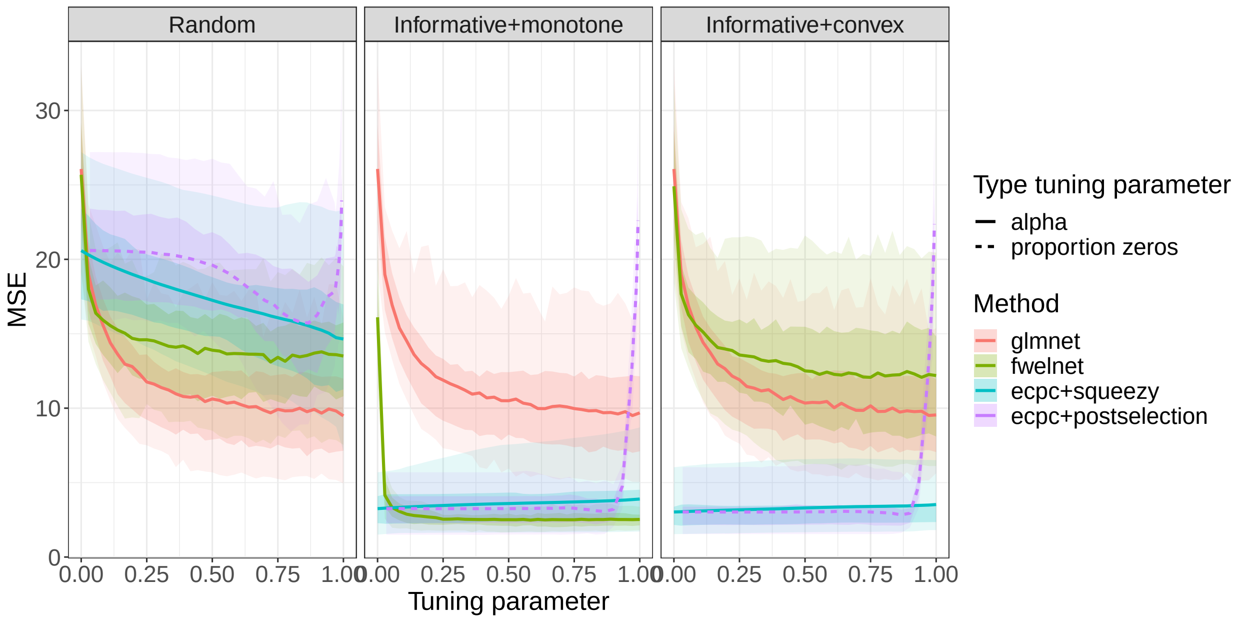


```
#Figure sensitivity vs precision for all simulations----
ggplot(dfPred)+# & dfEst$method=="ML",])+
  aes(col=method, shape=TypeTuning2)+
  facet_grid(.~Codata)+
  geom_line(aes(y=meansensitivity,x=1-meanprecision),linewidth=ls, alpha=0.2)+ #mean
  geom_point(aes(y=meansensitivity,x=1-meanprecision),size=ps,alpha=0.8)+ #mean
  guides(color=guide_legend(title="Method"),
         shape=guide_legend(title="Type tuning parameter"))+
  #geom_line(aes(y=meanVk),size=1)+ #mean
  labs(y="Sensitivity",x="1-precision")+
  theme_bw()+
  theme(axis.text.x=element_text(size=ts),
        axis.text.y=element_text(size=ts),
        axis.title.x=element_text(size=ts+2),
        axis.title.y=element_text(size=ts+2),
        legend.text=element_text(size=ts),
        legend.title=element_text(size=ts+2),
        strip.text=element_text(size=ts))#,
```

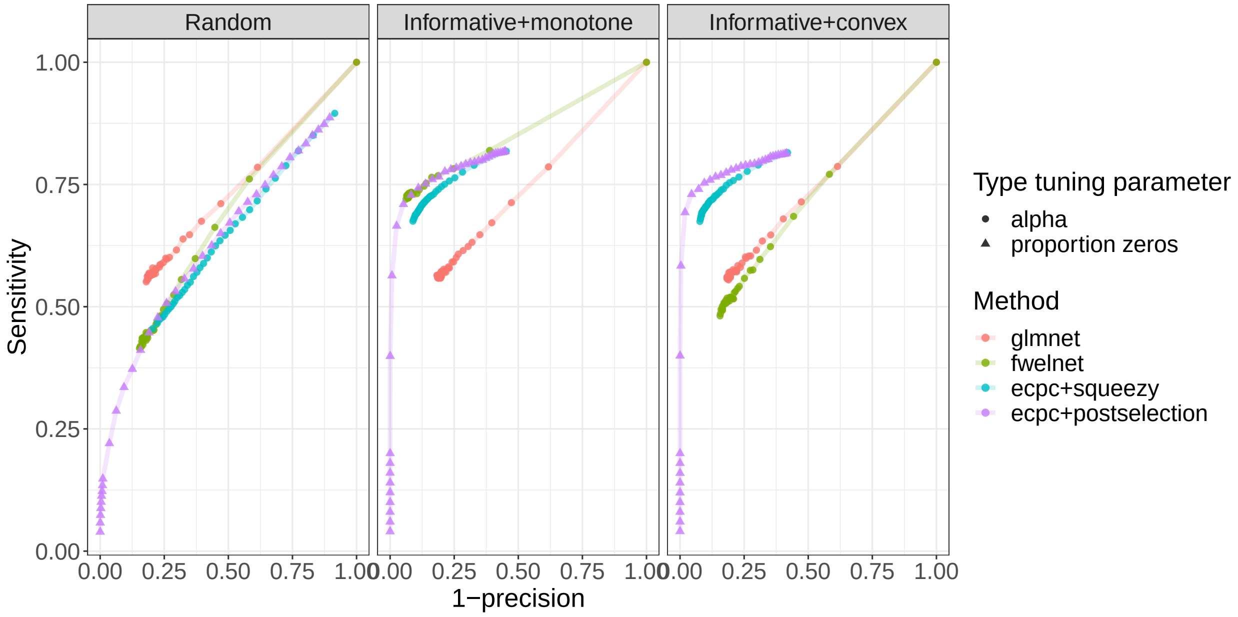

Simulation study 3: Computation time and memory costs

This section reproduces the figures on computation time and memory costs for a varying number of samples n or number of variables p, for the following models:

- ecpc, linear co-data model
- ecpc, generalised additive co-data model (gam)
- ecpc, shape constrained additive co-data model (scam) with positivity constraints
- glmnet, co-data agnostic ridge
- glmnet, co-data agnostic lasso

As co-data, we use one source of the true effect size.

To rerun the analysis and generation of toy data, set the following variables to `TRUE`:

```
run_n <- F
run_p <- F
generateData <- TRUE
```

Load libraries

First, load the libraries needed for this simulation study:

```
#Load libraries-----
library(peakRAM) #for keeping track of time and peak memory
#> Warning: package 'peakRAM' was built under R version 4.2.2
library(ecpc)
library(dplyr) #for data wrangling results
library(ggplot2) #for plotting results
library(ggpubr)
library(RColorBrewer) #for plotting results
```

Generate data

We generate data from a normal distribution for response $Y \in \mathbb{R}^n$, data $X \in \mathbb{R}^{n \times p}$ and regression coefficients $\beta \in \mathbb{R}^p$:

$$\beta_j \overset{i.i.d.}{\sim} N(0, 0.1),\ j = 1, .., p, \tag{1}$$

$$X_{i,j} \overset{i.i.d.}{\sim} N(0, 1),\ i = 1, .., n,\ j = 1, .., p, \tag{2}$$

$$Y_i \overset{ind.}{\sim} N(X_{i,:}\beta, 1),\ i = 1, .., n. \tag{3}$$

```
# Simulate toy data ------
p_all <- c(1000, 2000, 5000, 10000, 20000, 50000, 100000)
n_all <- c(50, 100, 200, 500, 1000)

n2<-100 #sample size test data set
sigma <- 1
tauglobal <- 0.1 #prior variance

#simulate all betas i.i.d. from beta_k~N(mean=0,sd=sqrt(0.1)):
set.seed(5463)
if(generateData){
  AllData_p <- list()
  for(i in 1:length(p_all)){
    p<-p_all[i] #number of covariates
    n<-200 #sample size training data set
    AllData_p[[i]] <- list()
    AllData_p[[i]]$beta <- rnorm(p,sd=sqrt(tauglobal))
    AllData_p[[i]]$Xctd <- matrix(rnorm(n*p) ,n,p)
    AllData_p[[i]]$X2ctd <- matrix(rnorm(n*p) ,n,p)
    AllData_p[[i]]$Y <- rnorm(n,mean=c(AllData_p[[i]]$Xctd%*%AllData_p[[i]]$beta),sd=sigma)
    AllData_p[[i]]$Y2 <- rnorm(n2,mean=c(AllData_p[[i]]$X2ctd%*%AllData_p[[i]]$beta),sd=sigma)
  }
  AllData_n <- list()

  p<-5000 #number of covariates
  betas <- rnorm(p,sd=sqrt(tauglobal))
  for(i in 1:length(n_all)){
    n<-n_all[i] #sample size training data set
    AllData_n[[i]] <- list()
    AllData_n[[i]]$beta <- betas
    AllData_n[[i]]$Xctd <- matrix(rnorm(n*p) ,n,p)
    AllData_n[[i]]$X2ctd <- matrix(rnorm(n*p) ,n,p)
    AllData_n[[i]]$Y <- rnorm(n,mean=c(AllData_n[[i]]$Xctd%*%AllData_n[[i]]$beta),sd=sigma)
    AllData_n[[i]]$Y2 <- rnorm(n2,mean=c(AllData_n[[i]]$X2ctd%*%AllData_n[[i]]$beta),sd=sigma)
  }
  save(AllData_p,AllData_n,file=paste(pathResults,"SimData_p_n.Rdata",sep=''))
}else{
  load(paste(pathResults,"SimData_p_n.Rdata",sep=''))
}
```

Run analysis for varying n

First, we fit the models for varying number of samples n and for a fixed number of variables $p = 5000$.

```r
#Run for p=5000, different n and check time and peak memory----
if(run_n){
  fname <- paste(pathResults, 'res_n.Rdata', sep='')
  df_n = data.frame("Method"=c(), "Time"=c(), "Peak_memory"=c(),
                   "n" = c(), "p"=c())
  p=5000
  #Compute co-data for p=5000
  Z <- abs(AllData_n[[1]]$beta)
  ZI <- cbind(rep(1,p),abs(AllData_n[[1]]$beta))
  Zs <- createZforSplines(values=Z, G=20, bdeg=3)
  #create 2nd order difference penalty matrix (same for all co-data)
  S1 <- createS(orderPen=2, G=20)
  Con.p <- createCon(G=20, shape="positive")

  for(i in 1:length(n_all)){
    n = n_all[i]

    #ecpc linear co-data model
    mem <- peakRAM({
      fit <- ecpc(Y=AllData_n[[i]]$Y,X=AllData_n[[i]]$Xctd,
                 Z=list(ZI), intrcpt.bam = FALSE,
                 model="linear",postselection=FALSE,est_beta_method = "multiridge")
    })
    temp <- data.frame("Method"="ecpc_linear", "Time"=mem[[2]], "Peak_memory"=mem[[4]],
                      "n"=dim(AllData_n[[i]]$Xctd)[1], p=dim(AllData_n[[i]]$Xctd)[2])
    df_n <- rbind(df_n, temp)

    #ecpc GAM co-data model
    mem <- peakRAM({
      fit <- ecpc(Y=AllData_n[[i]]$Y,X=AllData_n[[i]]$Xctd,
                  Z=list(Zs),
                  paraPen=list(Z1=list(S1=S1)),
                  bam.method="ML", intrcpt.bam = FALSE,
                  model="linear",postselection=FALSE,
                  est_beta_method = "multiridge")
    })
    temp <- data.frame("Method"="ecpc_gam", "Time"=mem[[2]], "Peak_memory"=mem[[4]],
                      "n"=dim(AllData_n[[i]]$Xctd)[1], p=dim(AllData_n[[i]]$Xctd)[2])
    df_n <- rbind(df_n, temp)

    #ecpc SCAM co-data model
    mem <- peakRAM({
      fit <- ecpc(Y=AllData_n[[i]]$Y,X=AllData_n[[i]]$Xctd,
                  Z=list(Zs),
                  paraPen=list(Z1=list(S1=S1)),
                  paraCon = list(Z1=Con.p),
                  intrcpt.bam = FALSE,
                  model="linear",postselection = FALSE,
                  est_beta_method = "multiridge")
    })
    temp <- data.frame("Method"="ecpc_scam", "Time"=mem[[2]], "Peak_memory"=mem[[4]],
                      "n"=dim(AllData_n[[i]]$Xctd)[1], p=dim(AllData_n[[i]]$Xctd)[2])
    df_n <- rbind(df_n, temp)
```

```
    #glmnet ridge
    mem <- peakRAM({
      fit.glmnet <- glmnet::cv.glmnet(y=AllData_n[[i]]$Y,x=AllData_n[[i]]$Xctd,
                                      family='gaussian',alpha=0)
      beta.glmnet <- coef(fit.glmnet,s="lambda.min", exact=TRUE)
    })
    temp <- data.frame("Method"="glmnet_ridge", "Time"=mem[[2]], "Peak_memory"=mem[[4]],
                       "n"=dim(AllData_n[[i]]$Xctd)[1], p=dim(AllData_n[[i]]$Xctd)[2])
    df_n <- rbind(df_n, temp)


    #glmnet lasso
    mem <- peakRAM({
      fit.glmnet <- glmnet::cv.glmnet(y=AllData_n[[i]]$Y,x=AllData_n[[i]]$Xctd,
                                      family='gaussian',alpha=1)
      beta.glmnet <- coef(fit.glmnet,s="lambda.min", exact=TRUE)
    })
    temp <- data.frame("Method"="glmnet_lasso", "Time"=mem[[2]], "Peak_memory"=mem[[4]],
                       "n"=dim(AllData_n[[i]]$Xctd)[1], p=dim(AllData_n[[i]]$Xctd)[2])
    df_n <- rbind(df_n, temp)

    save(df_n, file=fname)
  }

}
```

Run analysis for varying p

Then, we fit the models for varying number of samples p and for a fixed number of samples $n = 200$.

```
#Run for n=200, different p and check time and peak memory----
if(run_p){
  fname <- paste(pathResults, 'res_p.Rdata', sep='')
  df_p = data.frame("Method"=c(), "Time"=c(), "Peak_memory"=c(),
                    "n" = c(), "p"=c())

  for(i in 1:length(p_all)){
    p = p_all[i]
    print(Sys.time(),' ',p_all[i])

    #Compute co-data for p
    Z <- abs(AllData_p[[i]]$beta)
    ZI <- cbind(rep(1,p),abs(AllData_p[[1]]$beta))
    Zs <- createZforSplines(values=c(Z), G=20, bdeg=3)
    #create 2nd order difference penalty matrix (same for all co-data)
    S1 <- createS(orderPen=2, G=20)
    Con.p <- createCon(G=20, shape="positive")

    #ecpc linear co-data model
    mem <- peakRAM({
      fit <- ecpc(Y=AllData_p[[i]]$Y,X=AllData_p[[i]]$Xctd,
                  Z=list(ZI), intrcpt.bam=FALSE,
                  model="linear",postselection=FALSE,est_beta_method = "multiridge")
```

```
})
temp <- data.frame("Method"="ecpc_linear", "Time"=mem[[2]], "Peak_memory"=mem[[4]],
                   "n"=dim(AllData_p[[i]]$Xctd)[1], p=dim(AllData_p[[i]]$Xctd)[2])
df_p <- rbind(df_p, temp)

#ecpc GAM co-data model
mem <- peakRAM({
  fit <- ecpc(Y=AllData_p[[i]]$Y,X=AllData_p[[i]]$Xctd,
              Z=list(Zs),
              paraPen=list(Z1=list(S1=S1)),
              bam.method="ML", intrcpt.bam = FALSE,
              model="linear",postselection=FALSE,
              est_beta_method = "multiridge")
})
temp <- data.frame("Method"="ecpc_gam", "Time"=mem[[2]], "Peak_memory"=mem[[4]],
                   "n"=dim(AllData_p[[i]]$Xctd)[1], p=dim(AllData_p[[i]]$Xctd)[2])
df_p <- rbind(df_p, temp)

#ecpc SCAM co-data model
mem <- peakRAM({
  fit <- ecpc(Y=AllData_p[[i]]$Y,X=AllData_p[[i]]$Xctd,
              Z=list(Zs),
              paraPen=list(Z1=list(S1=S1)),
              paraCon = list(Z1=Con.p),
              intrcpt.bam = FALSE,
              model="linear",postselection = FALSE,
              est_beta_method = "multiridge")
})
temp <- data.frame("Method"="ecpc_scam", "Time"=mem[[2]], "Peak_memory"=mem[[4]],
                   "n"=dim(AllData_p[[i]]$Xctd)[1], p=dim(AllData_p[[i]]$Xctd)[2])
df_p <- rbind(df_p, temp)

#glmnet ridge
mem <- peakRAM({
  fit.glmnet <- glmnet::cv.glmnet(y=AllData_p[[i]]$Y,x=AllData_p[[i]]$Xctd,
                                  family='gaussian',alpha=0)
  beta.glmnet <- coef(fit.glmnet,s="lambda.min", exact=TRUE)
})
temp <- data.frame("Method"="glmnet_ridge", "Time"=mem[[2]], "Peak_memory"=mem[[4]],
                   "n"=dim(AllData_p[[i]]$Xctd)[1], p=dim(AllData_p[[i]]$Xctd)[2])
df_p <- rbind(df_p, temp)


#glmnet lasso
mem <- peakRAM({
  fit.glmnet <- glmnet::cv.glmnet(y=AllData_p[[i]]$Y,x=AllData_p[[i]]$Xctd,
                                  family='gaussian',alpha=1)
  beta.glmnet <- coef(fit.glmnet,s="lambda.min", exact=TRUE)
})
temp <- data.frame("Method"="glmnet_lasso", "Time"=mem[[2]], "Peak_memory"=mem[[4]],
                   "n"=dim(AllData_p[[i]]$Xctd)[1], p=dim(AllData_p[[i]]$Xctd)[2])
df_p <- rbind(df_p, temp)
```

```
    save(df_p, file=fname)
  }

}
```

Plot results

Plot parameters

First, set some general parameters for plotting:

```
#Plots: general parameters----
wdth<-600
hght<-wdth*5/8
wdthpdf <- wdth/75
hghtpdf <- hght/75
ts <- 16 #basis text size in figures
ls <- 1.5 #basis line size in figures
ps <- 2 #basis point size in figures
sz <- 2 #point size
strk <- 1.5 #stroke size
```

Load results for plotting

Load the pre-saved results with:

```
#Load data for plots----
#different n, p=5000 fixed
fname <- paste(pathResults, 'res_n.Rdata', sep='')
load(fname)
df_n$Method <- factor(df_n$Method,levels=unique(df_n$Method),
                        labels=unique(df_n$Method))

#different p, n=200 fixed
fname <- paste(pathResults, 'res_p.Rdata', sep='')
load(fname)
df_p$Method <- factor(df_p$Method,levels=unique(df_p$Method),
                      labels=unique(df_p$Method))
```

Figure time vs n

```
p1 <- ggplot(df_n)+
  aes(x=n,y=Time,col=Method,linetype=Method,shape=Method)+
  geom_line(linewidth=1.2)+
  geom_point(size=ps*1.5)+
  scale_color_manual(values=c('black','black','black','grey40','grey40'))+
  scale_linetype_manual(values=c(1,2,3,1,2))+
  scale_shape_manual(values=c(15,16,17,15,16))+
  #scale_y_log10()+
  labs(y="Time (s)",x="n")+
  theme_bw()+
  theme(axis.text.x=element_text(size=ts),
        axis.text.y=element_text(size=ts),
        axis.title.x=element_text(size=ts+2),
        axis.title.y=element_text(size=ts+2),
```

```
        legend.text=element_text(size=ts),
        legend.title=element_text(size=ts+2),
        strip.text=element_text(size=ts),
        legend.key.width = unit(2, "cm"))#,
p1
```

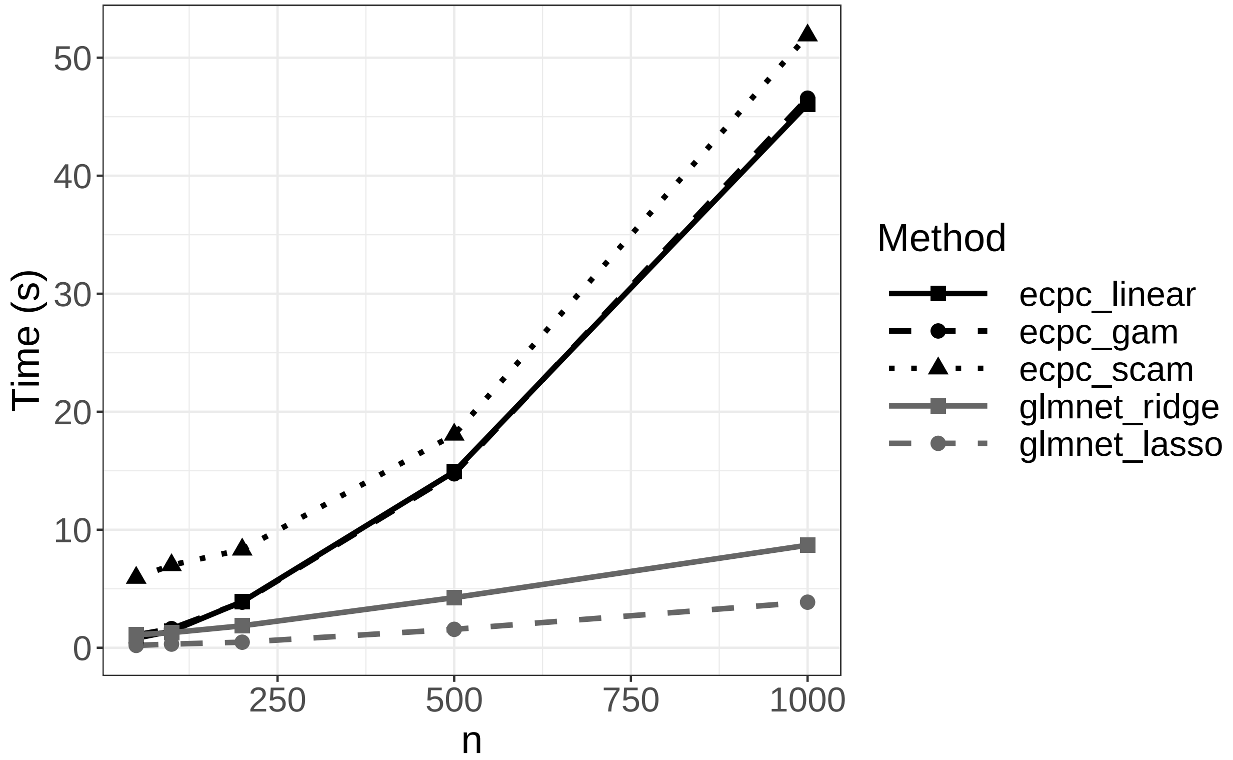

Figure memory vs n

```
p2 <- ggplot(df_n)+
  aes(x=n,y=Peak_memory,col=Method,linetype=Method,shape=Method)+
  geom_line(linewidth=1.2)+
  geom_point(size=ps*1.5)+
  scale_color_manual(values=c('black','black','black','grey40','grey40'))+
  scale_linetype_manual(values=c(1,2,3,1,2))+
  scale_shape_manual(values=c(15,16,17,15,16))+
  #scale_y_log10()+
  labs(y="Peak memory (MiB)",x="n")+
  theme_bw()+
  theme(axis.text.x=element_text(size=ts),
        axis.text.y=element_text(size=ts),
        axis.title.x=element_text(size=ts+2),
        axis.title.y=element_text(size=ts+2),
        legend.text=element_text(size=ts),
        legend.title=element_text(size=ts+2),
        strip.text=element_text(size=ts),
        legend.key.width = unit(2, "cm"))#,
p2
```

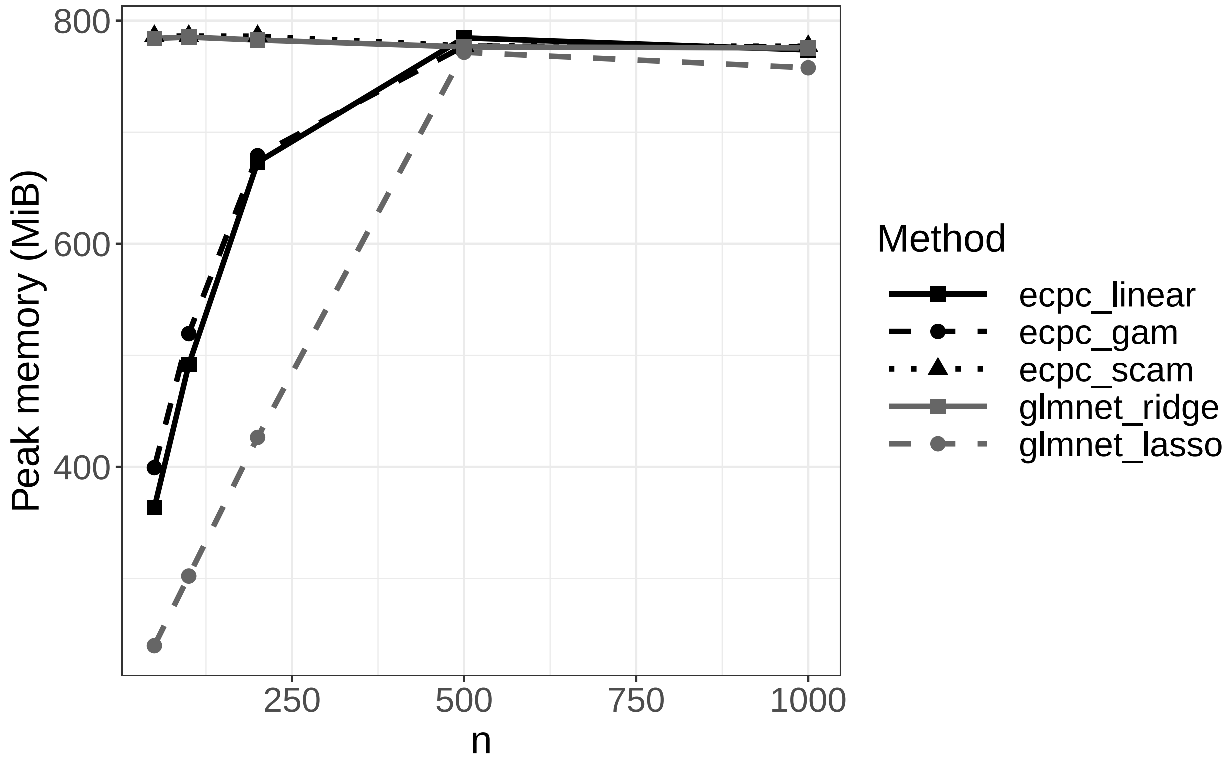

Figure time vs p

```
p3 <- ggplot(df_p)+
  aes(x=p,y=Time,col=Method,linetype=Method,shape=Method)+
  geom_line(linewidth=1.2)+
  geom_point(size=ps*1.5)+
  scale_color_manual(values=c('black','black','black','grey40','grey40'))+
  scale_linetype_manual(values=c(1,2,3,1,2))+
  scale_shape_manual(values=c(15,16,17,15,16))+
  #scale_y_log10()+
  labs(y="Time (s)",x="p")+
  theme_bw()+
  theme(axis.text.x=element_text(size=ts),
        axis.text.y=element_text(size=ts),
        axis.title.x=element_text(size=ts+2),
        axis.title.y=element_text(size=ts+2),
        legend.text=element_text(size=ts),
        legend.title=element_text(size=ts+2),
        strip.text=element_text(size=ts),
        legend.key.width = unit(2, "cm"))#,
p3
```

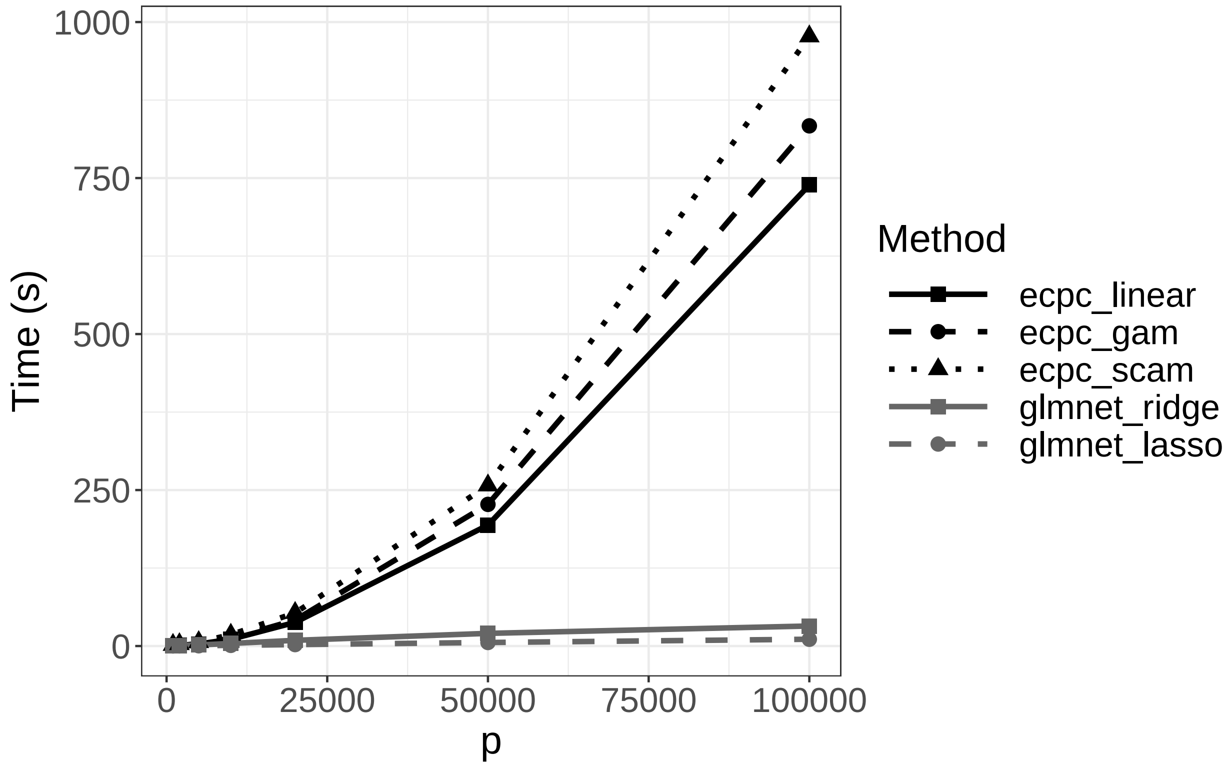

Figure memory vs p

```
p4 <- ggplot(df_p)+
  aes(x=p,y=Peak_memory,col=Method,linetype=Method,shape=Method)+
  geom_line(linewidth=1.2)+
  geom_point(size=ps*1.5)+
  scale_color_manual(values=c('black','black','black','grey40','grey40'))+
  scale_linetype_manual(values=c(1,2,3,1,2))+
  scale_shape_manual(values=c(15,16,17,15,16))+
  #scale_y_log10()+
  labs(y="Peak memory (MiB)",x="p")+
  theme_bw()+
  theme(axis.text.x=element_text(size=ts),
        axis.text.y=element_text(size=ts),
        axis.title.x=element_text(size=ts+2),
        axis.title.y=element_text(size=ts+2),
        legend.text=element_text(size=ts),
        legend.title=element_text(size=ts+2),
        strip.text=element_text(size=ts),
        legend.key.width = unit(2, "cm"))#,
p4
```

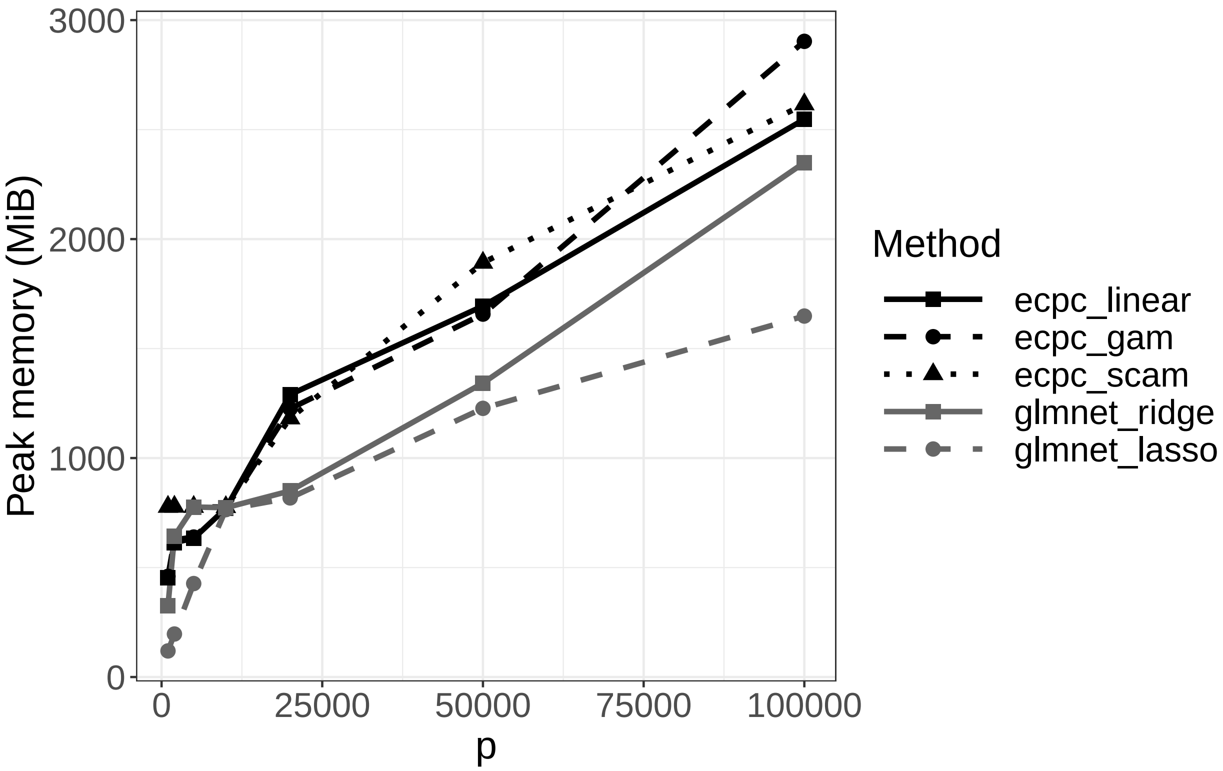

Combine plots

```
p <- ggarrange(
  p1, p2, p3, p4,
  common.legend = TRUE, legend = "bottom"
)
p
```

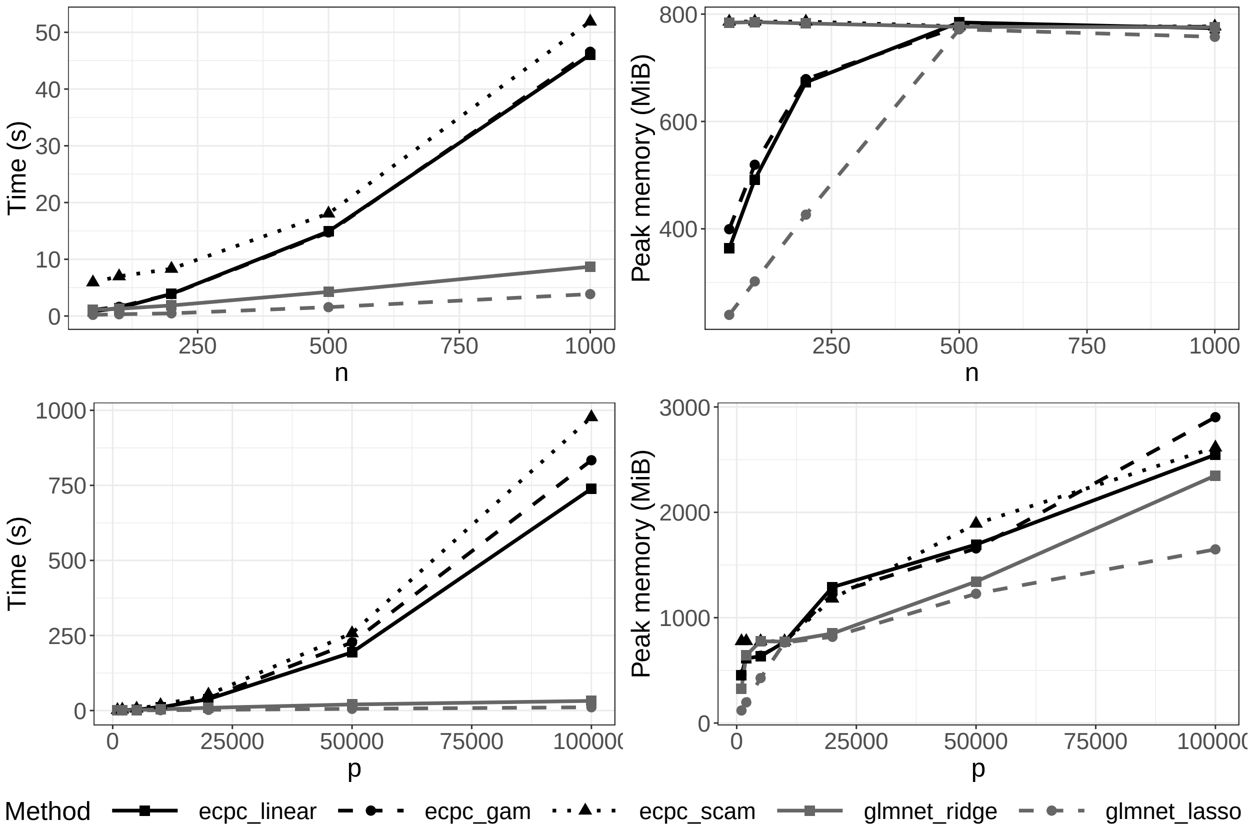

References

[1] van Nee, M.M., Wessels, L.F.A., van de Wiel, M.A.: ecpc: an R-package for generic co-data models for high-dimensional prediction

[2] van Nee, M.M., Wessels, L.F.A., van de Wiel, M.A.: Flexible co-data learning for high-dimensional prediction. Statistics in Medicine 40(26), 5910–5925 (2021)

[3] Eilers, P.H., Marx, B.D.: Practical Smoothing: The Joys of P-splines. Cambridge University Press, Cambridge (2021)
