# Supplementary material for: ecpc: an R-package for generic co-data models for high-dimensional prediction
Source: BMC Bioinformatics. 2023 Apr 26;24:172. doi: 10.1186/s12859-023-05289-x (PMC10134536; doi:10.1186/s12859-023-05289-x)
Supplement: Supplementary file 3 — Additional file 3. Vignette as pdf file to reproduce the analysis example. [file 12859_2023_5289_MOESM3_ESM.pdf]

Vignette ecpc: analysis example

Mirrelijn van Nee

Contents

Overview 1

Analysis example 1

- Install and load R-packages 1
- Load data 2
- Prepare co-data 2
- Fit model 3
- Plot co-data source contributions 4
- Predictions 4
- Variable selection 4

Analysis other co-data models 4

- Run analysis 5
- Set plotting parameters 11
- Load presaved results 11
- Figure co-data source contributions 12
- Figure prediction performance 13

References 15

Overview

This vignette can be used to reproduce the analysis example from [1].

We demonstrate the different co-data models by applying the method to an application in classifying lymph node metastasis (coded 1) from other types of cancer (coded 0). We use the data readily available from the R-package CoRF, providing high-dimensional RNA expression training data for $p = 12838$ probes and $n = 133$ patients, and validation data for $n_2 = 97$ patients.

Analysis example

Install and load R-packages

First install the following packages needed below.

```
if(!requireNamespace("devtools")) install.packages("devtools")
library("devtools")
install_github("DennisBeest/CoRF")
if(!requireNamespace("ecpc")) install.packages("ecpc")
if(!requireNamespace("squeezy")) install.packages("squeezy")
if(!requireNamespace("dplyr")) install.packages("dplyr")
if(!requireNamespace("ggplot2")) install.packages("ggplot2")
```

```r
if(!requireNamespace("ggpubr")) install.packages("ggpubr")
if(!requireNamespace("scales")) install.packages("scales")
if(!requireNamespace("pROC")) install.packages("pROC")
if(!requireNamespace("ggh4x")) install_github("teunbrand/ggh4x")
```

Load the packages:

```r
library("ecpc")
library("squeezy")
library("CoRF")
library("ggplot2")
#> Warning: package 'ggplot2' was built under R version 4.2.2
library("dplyr")
#>
#> Attaching package: 'dplyr'
#> The following objects are masked from 'package:stats':
#>
#>     filter, lag
#> The following objects are masked from 'package:base':
#>
#>     intersect, setdiff, setequal, union
library("pROC")
#> Type 'citation("pROC")' for a citation.
#>
#> Attaching package: 'pROC'
#> The following objects are masked from 'package:stats':
#>
#>     cov, smooth, var
library("ggpubr")
library("scales")
#> Warning: package 'scales' was built under R version 4.2.2
library("ggh4x")
pathResults <- "./Results_analysis_example/"
```

Load data

Then we load the data and transform the response of the validation data set to match the format of the training data:

```r
data("LNM_Example")
RespValidationNum <- as.numeric(RespValidation)-1
```

The data provide three different sources of co-data: - `Signature`: a published signature of genes. Probes either match a gene in the signature or not. - `Correlation`: cis-correlations between RNA expression and copy number. - `P-values`: p-values from an external, similar study, using a different technique to measure RNA expression.

Prepare co-data

First, we prepare the co-data.

Categorical co-data: gene signature

The first co-data source is categorical. We use the helper functions `createGroupset()` and `createZforGroupset()` to transform the vector of categories to a group set and co-data matrix:

```
GroupsetSig <- createGroupset(as.factor(CoDataTrain$RoepmanGenes))
#> [1] "Summary of group sizes:"
#>     0     1
#> 12324   514
Z_sig <- createZforGroupset(GroupsetSig)
```

Continuous co-data: correlations

The second co-data source with correlations is continuous. We use 20 splines to flexibly model the relation between the prior variance and the correlations. Furthermore, we constrain the relation to be positively monotonically increasing, as we expect larger correlations to be of more importance. The co-data spline basis matrix, difference penalty matrix and constraints are obtained by:

```
G <- 20 #number of splines
Z_cor <- createZforSplines(values=CoDataTrain$Corrs, G=G)
S1_cor <- createS(orderPen=2, G=G)
Con_cor <- createCon(G=G, shape="positive+monotone.i")
```

Continuous co-data: p-values

We prepare the co-data with p-values similarly to the correlations, but constrain the relation to be positively monotonically decreasing, as we expect smaller p-values to be of more importance. We set the p-value of two variables that have missing p-values to the maximum observed p-value and compute the co-data (related) matrices:

```
CoDataTrain$pvalsVUmc[is.na(CoDataTrain$pvalsVUmc)] <-
  max(CoDataTrain$pvalsVUmc, na.rm=TRUE)
Z_pvals <- createZforSplines(values=CoDataTrain$pvalsVUmc, G=G)
S1_pvals <- createS(G=G)
Con_pvals <- createCon(G=G, shape="positive+monotone.d")
```

Concatenate co-data sources

As the last step of the preparation of the co-data, we save the continuous co-data variables in a separate list for the `plot()` function that we use below, and concatenate the co-data matrices in a list:

```
values <- list("Signature" = NULL,
               "Correlation" = CoDataTrain$Corrs,
               "P-values" = CoDataTrain$pvalsVUmc)
Z_all <- list("Signature" = Z_sig,
              "Correlation" = Z_cor,
              "P-values" = Z_pvals)
```

Fit model

Then we fit the model and select variables a posteriori, with a range of the maximum number of variables that should be selected defined in the input argument `maxsel`:

```
set.seed(3)
maxSel <- c(2:10,10*(2:10)) #maximum posterior selected variables
Res<-ecpc(Y=RespTrain, X=TrainData, Z=Z_all,
          paraPen = list(Z2=list(S1=S1_cor), Z3=list(S1=S1_pvals)),
          paraCon = list(Z2=Con_cor, Z3=Con_pvals),
          Y2=RespValidationNum, X2=ValidationData,
          maxsel=maxSel)
```

```r
maxSel <- c(2:10,10*(2:10)) #maximum posterior selected variables
fname <- paste(pathResults,"Res.Rdata",sep='')
load(fname)
```

Plot co-data source contributions

We plot the contributions from each co-data source:

```r
plot(Res, show="priorweights", Z=Z_all, values=values)
#> [1] "Prior variance contribution per group/continuous values and co-data source is plotted."
```

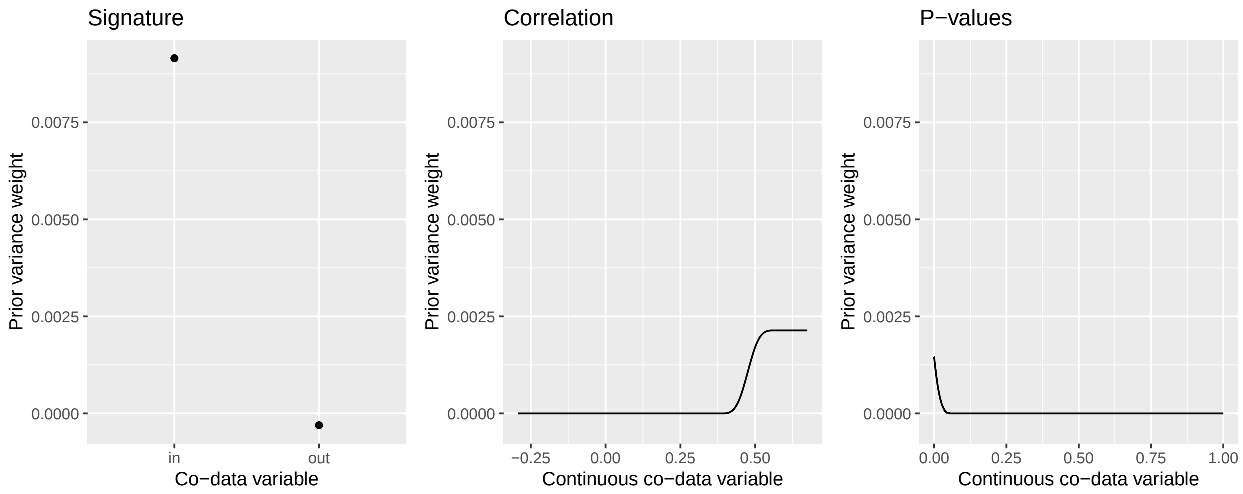

Predictions

The predicted values for the validation data are given in `Res\$Ypred` as `X2` was provided to `ecpc()`. Alternatively, the predictions may be retrieved with the method `predict()`:

```r
Ypred <- predict(Res, X2=ValidationData)
```

Variable selection

The posterior selected variables are given in `Res\$betaPost` as `maxsel` was provided to `ecpc()`. Alternatively, the same posterior selection method may be performed with the function `postSelect()` on the fitted ecpc-object `Res`:

```r
sparseModels <- postSelect(Res, X=TrainData, Y=RespTrain, maxsel=maxSel)
```

A second approach for variable selection is to use `squeezy()` to transform the ridge penalties to elastic net penalties, with elastic net tuning parameter α. As mentioned above, in practice one may try a range of tuning parameters to choose the sparsest model with close to optimal performance. For this example, we include the lasso model ($\alpha = 1$):

```r
sparseModel2 <- squeezy(Y=RespTrain, X=TrainData, alpha=1,
                        lambdas=Res$penalties,
                        X2=ValidationData, Y2=RespValidationNum)
```

Analysis other co-data models

Instead of fitting monotone and positively constrained functions for the correlation and p-values co-data, one could consider other co-data models. We run the model for for three different settings:

1. a generalised additive co-data model (GAM), i.e. without constraints (`run_withoutConstraints` in the code below);
2. a shape-constrained additive co-data model (SCAM) with positivity constraints (`run_withConstraints`);
3. a SCAM with positivity and monotonicity constraints, as in the exemplified code above (`run_withConstraints2`).

The results include the dense models obtained with `ecpc()` and a co-data agnostic ordinary ridge model, and sparse models for a range of posterior selected variables obtained with `postSelect()`, the lasso model obtained with transformed penalties from `squeezy()` and a co-data agnostic lasso model obtained with glmnet (`run_lasso`).

Run analysis

The figures below are reproduced using presaved results from the analysis to save time. To rerun the analysis, run the following code-block with `run_withoutConstraints`, `run_withConstraints`, `run_withConstraints2` and `run_lasso` set to `TRUE`:

```
#full analyses for 4 different settings:
run_withConstraints <- F
run_withoutConstraints <- F
run_withConstraints2 <- F
run_lasso <- F

#Load data from CoRF package----
data(LNM_Example)
#make response of RespValidation numeric such that its format matches that of RespTrain
RespValidationNum <- as.numeric(RespValidation)-1
maxSel <- c(2:10,10*(2:10)) #maximum selected variables
G_all <- c(20,50) #use 20 or 50 spline basis functions
n <- dim(TrainData)[1]
p <- dim(TrainData)[2]

#Run all analyses and save results for plotting----
Z_all <- list(); values <- list()
Z_all_rank <- list(); values_rank <- list()
for(g in 1:length(G_all)){
  #Format co-data----
  set.seed(3)
  G <- G_all[g] #number of splines
  #co-data source 1: genes corresponding to a known signature or not
  GroupsetSig <- createGroupset(as.factor(CoDataTrain$RoepmanGenes)) #list of groups
  Z_sig <- createZforGroupset(GroupsetSig) #co-data matrix
  colnames(Z_sig) <- c("out","in")

  #Co-data 2: correlation
  Z_cor <- createZforSplines(values=CoDataTrain$Corrs, G=G)
  S1_cor <- createS(orderPen=2, G=G) #create difference penalty matrix
  Con_cor <- createCon(G=G, shape="positive+monotone.i")
  Con_cor2 <- createCon(G=G, shape="positive")

  #Co-data 2a: rank correlation
  ecdfZ <- ecdf(CoDataTrain$Corrs)
  ZRankCorrs <- ecdfZ(CoDataTrain$Corrs)
  Z_cor_rank <- createZforSplines(values=ZRankCorrs, G=G)
  S1_cor_rank <- createS(G=G) #create difference penalty matrix
```

```r
#Co-data 3: p-values of external study using a different measurement technique
#2 probes have NA p-value, set to maximum observed p-value (approx. 1)
CoDataTrain$pvalsVUmc[is.na(CoDataTrain$pvalsVUmc)] <- max(CoDataTrain$pvalsVUmc,na.rm=TRUE)
Z_pvals <- createZforSplines(values=CoDataTrain$pvalsVUmc, G=G)
S1_pvals <- createS(G=G) #create difference penalty matrix
Con_pvals <- createCon(G=G, shape="positive+monotone.d")
Con_pvals2 <- createCon(G=G, shape="positive")

#Co-data 3a: rank p-values
ZRankPvals <- ecdf(CoDataTrain$pvalsVUmc)(CoDataTrain$pvalsVUmc)
Z_pvals_rank <- createZforSplines(values=ZRankPvals, G=G)
S1_pvals_rank <- createS(G=G) #create difference penalty matrix


#save continuous co-data values in seperate list for plotting
values[[g]] <- list("Signature" = NULL,
                    "Correlation" = CoDataTrain$Corrs,
                    "P-values" = CoDataTrain$pvalsVUmc)
values_rank[[g]] <- list("Signature" = NULL,
                         "Correlation" = ZRankCorrs,
                         "P-values" = ZRankPvals)

Z_all[[g]] <- list("Signature" = Z_sig,
                   "Correlation" = Z_cor,
                   "P-values" = Z_pvals)

Z_all_rank[[g]] <- list("Signature" = Z_sig,
                        "Correlation" = Z_cor_rank,
                        "P-values" = Z_pvals_rank)



#Fit model 1: all three co-data matrices, with constraints----
fname <- paste("Res1G",g,".Rdata",sep="")
print(fname)

if(run_withConstraints){
  #ecpc
  tic <- proc.time()[[3]]
  Res<-ecpc(Y=RespTrain, X=TrainData, #training data
            Z=Z_all[[g]],
            paraPen = list(Z2=list(S1=S1_cor), Z3=list(S1=S1_pvals)),
            paraCon = list(Z2=Con_cor, Z3=Con_pvals),
            Y2=RespValidationNum, X2=ValidationData, #test data
            maxsel=maxSel) #maximum number of selected covariates for posterior selection
  toc <- proc.time()[[3]];
  print(toc - tic) #elapsed time
  Res$time <- toc-tic

  plot(Res, show = "priorweights", Z=Z_all[[g]], values=values[[g]])

  #with squeezy for lasso
  tic <- proc.time()[[3]]
```

```
  sparseModel2 <- squeezy(Y=RespTrain, X=TrainData, alpha=1,
                         lambdas=Res$penalties,
                         X2=ValidationData, Y2=RespValidationNum)
  toc <- proc.time()[[3]]-tic

  #concatenate results on predictions in data frame
  ntest <- length(RespValidation) #number of test samples in the validation data
  p<-dim(CoDataTrain)[1] #number of genes
  df<-data.frame("Ypred"= c(Res$Ypred, Res$Ypredridge,
                          c(Res$YpredPost),c(sparseModel2$YpredApprox)),
               "Truth" = rep(RespValidationNum, 2+length(maxSel)+1),
               "Sample" = rep(1:ntest,2+length(maxSel)+1),
               "NumberSelectedVars" =rep(c(sum(Res$beta!=0),p,maxSel,
                                         sum(sparseModel2$betaApprox!=0)),each=ntest),
               "Method" = as.factor(rep(c("ecpc","ordinary.ridge",rep("ecpc",length(maxSel)),
                                         "ecpc+squeezy"),each=ntest)),
               "Sparsity" = as.factor(rep(c("dense","dense", rep("sparse",length(maxSel)),
                                          "sparse"),each=ntest)),
               "MethodxNumberVars" = as.factor(rep(c("ecpc","ordinary.ridge",
                                                    paste("ecpc",maxSel,"vars",sep=""),
                                                    "ecpc+squeezy"),each=ntest)))

  #Compute AUC for each method and store in a data frame
  dfAUC <- data.frame()
  for (i in levels(df$MethodxNumberVars)) {
    temp <- data.frame("Method" = unique(df$Method[df$MethodxNumberVars==i]),
                     "Sparsity"=unique(df$Sparsity[df$MethodxNumberVars==i]),
                     "MethodxNumberVars"=i)
    rocpROC <- pROC::roc(predictor = df$Ypred[df$MethodxNumberVars == i],
                       response = df$Truth[df$MethodxNumberVars == i],
                       smooth = F, auc = T, levels = c(0, 1), direction = "<")
    temp$AUC <- rocpROC$auc[1]
    temp$NumberSelectedVars <- mean(df$NumberSelectedVars[df$MethodxNumberVars == i])
    dfAUC <- rbind(dfAUC, temp)
  }
  lims <- range(dfAUC$AUC,0.5) #set limits for AUC plots
  dfAUC$Codata <- "all"

  save(Res,df,dfAUC,file=fname)
}


#Fit model 2: all three co-data matrices, no constraints----
fname <- paste("Res2G",g,".Rdata",sep="")
print(fname)

if(run_withoutConstraints){
  tic <- proc.time()[[3]]
  Res<-ecpc(Y=RespTrain, X=TrainData, #training data
          Z=Z_all[[g]],
          paraPen = list(Z2=list(S1=S1_cor), Z3=list(S1=S1_pvals)),
          Y2=RespValidationNum, X2=ValidationData, #test data
          maxsel=maxSel) #maximum number of selected covariates for posterior selection
```

```
  toc <- proc.time()[[3]];
  print(toc - tic) #elapsed time
  Res$time <- toc-tic

  plot(Res, show = "priorweights", Z=Z_all[[g]], values=values[[g]])

  #with squeezy for lasso
  tic <- proc.time()[[3]]
  sparseModel2 <- squeezy(Y=RespTrain, X=TrainData, alpha=1,
                          lambdas=Res$penalties,
                          X2=ValidationData, Y2=RespValidationNum)
  toc <- proc.time()[[3]]-tic

  #concatenate results on predictions in data frame
  ntest <- length(RespValidation) #number of test samples in the validation data
  p<-dim(CoDataTrain)[1] #number of genes
  df<-data.frame("Ypred"= c(Res$Ypred, Res$Ypredridge,
                            c(Res$YpredPost),c(sparseModel2$YpredApprox)),
                 "Truth" = rep(RespValidationNum, 2+length(maxSel)+1),
                 "Sample" = rep(1:ntest,2+length(maxSel)+1),
                 "NumberSelectedVars" = rep(c(sum(Res$beta!=0),p,maxSel,
                                              sum(sparseModel2$betaApprox!=0)),each=ntest),
                 "Method" = as.factor(rep(c("ecpc","ordinary.ridge",rep("ecpc",length(maxSel)),
                                             "ecpc+squeezy"),each=ntest)),
                 "Sparsity" = as.factor(rep(c("dense","dense", rep("sparse",length(maxSel)),
                                               "sparse"),each=ntest)),
                 "MethodxNumberVars" = as.factor(rep(c("ecpc","ordinary.ridge",
                                                        paste("ecpc",maxSel,"vars",sep=""),
                                                        "ecpc+squeezy"),each=ntest)))

  #Compute AUC for each method and store in a data frame
  dfAUC <- data.frame()
  for (i in levels(df$MethodxNumberVars)) {
    temp <- data.frame("Method" = unique(df$Method[df$MethodxNumberVars==i]),
                       "Sparsity"=unique(df$Sparsity[df$MethodxNumberVars==i]),
                       "MethodxNumberVars"=i)
    rocpROC <- pROC::roc(predictor = df$Ypred[df$MethodxNumberVars == i],
                         response = df$Truth[df$MethodxNumberVars == i],
                         smooth = F, auc = T, levels = c(0, 1), direction = "<")
    temp$AUC <- rocpROC$auc[1]
    temp$NumberSelectedVars <- mean(df$NumberSelectedVars[df$MethodxNumberVars == i])
    dfAUC <- rbind(dfAUC, temp)
  }
  lims <- range(dfAUC$AUC,0.5) #set limits for AUC plots
  dfAUC$Codata <- "all"

  save(Res,df,dfAUC,file=fname)
}


#Fit model 3: all three co-data matrices, with positivity constraints----
fname <- paste("Res3G",g,".Rdata",sep="")
print(fname)
```

```r
if(run_withConstraints2){
  tic <- proc.time()[[3]]
  Res<-ecpc(Y=RespTrain, X=TrainData, #training data
            Z=Z_all[[g]],
            paraPen = list(Z2=list(S1=S1_cor), Z3=list(S1=S1_pvals)),
            paraCon = list(Z2=Con_cor2, Z3=Con_pvals2),
            Y2=RespValidationNum, X2=ValidationData, #test data
            maxsel=maxSel) #maximum number of selected covariates for posterior selection
  toc <- proc.time()[[3]];
  print(toc - tic) #elapsed time
  Res$time <- toc-tic

  plot(Res, show = "priorweights", Z=Z_all[[g]], values=values[[g]])

  #with squeezy for lasso
  tic <- proc.time()[[3]]
  sparseModel2 <- squeezy(Y=RespTrain, X=TrainData, alpha=1,
                          lambdas=Res$penalties,
                          X2=ValidationData, Y2=RespValidationNum,reCV=T)
  toc <- proc.time()[[3]]-tic

  #concatenate results on predictions in data frame
  ntest <- length(RespValidation) #number of test samples in the validation data
  p<-dim(CoDataTrain)[1] #number of genes
  df<-data.frame("Ypred"= c(Res$Ypred, Res$Ypredridge,
                            c(Res$YpredPost),c(sparseModel2$YpredApprox)),
                 "Truth" = rep(RespValidationNum, 2+length(maxSel)+1),
                 "Sample" = rep(1:ntest,2+length(maxSel)+1),
                 "NumberSelectedVars" = rep(c(sum(Res$beta!=0),p,maxSel,
                                              sum(sparseModel2$betaApprox!=0)),each=ntest),
                 "Method" = as.factor(rep(c("ecpc","ordinary.ridge",rep("ecpc",length(maxSel)),
                                            "ecpc+squeezy"),each=ntest)),
                 "Sparsity" = as.factor(rep(c("dense","dense", rep("sparse",length(maxSel)),
                                              "sparse"),each=ntest)),
                 "MethodxNumberVars" = as.factor(rep(c("ecpc","ordinary.ridge",
                                                       paste("ecpc",maxSel,"vars",sep=""),
                                                       "ecpc+squeezy"),each=ntest)))

  #Compute AUC for each method and store in a data frame
  dfAUC <- data.frame()
  for (i in levels(df$MethodxNumberVars)) {
    temp <- data.frame("Method" = unique(df$Method[df$MethodxNumberVars==i]),
                       "Sparsity"=unique(df$Sparsity[df$MethodxNumberVars==i]),
                       "MethodxNumberVars"=i)
    rocpROC <- pROC::roc(predictor = df$Ypred[df$MethodxNumberVars == i],
                         response = df$Truth[df$MethodxNumberVars == i],
                         smooth = F, auc = T, levels = c(0, 1), direction = "<")
    temp$AUC <- rocpROC$auc[1]
    temp$NumberSelectedVars <- mean(df$NumberSelectedVars[df$MethodxNumberVars == i])
    dfAUC <- rbind(dfAUC, temp)
  }
  lims <- range(dfAUC$AUC,0.5) #set limits for AUC plots
  dfAUC$Codata <- "all"
```

```
    save(Res,df,dfAUC,file=fname)
  }

  #Fit lasso----
  fname <- paste("Resg",g,"Lasso.Rdata",sep="")
  print(fname)

  if(run_lasso){
    ntest <- length(RespValidation) #number of test samples in the validation data
    p<-dim(CoDataTrain)[1] #number of genes

    tic <- proc.time()[[3]]
    fit.glmnet <- glmnet::glmnet(y=RespTrain,x=TrainData,
                                 family="binomial",alpha=1)
    dfAUC <- data.frame()
    for(k in 2:length(fit.glmnet$lambda)){
      beta.glmnet <- coef(fit.glmnet,s=fit.glmnet$lambda[k], exact=TRUE)
      Ypred.glmnet <- c(predict(fit.glmnet, newx = ValidationData,
                             s = fit.glmnet$lambda[k], type="response", exact=TRUE))

      temp <- data.frame("Method" = "lasso",
                         "Sparsity"= "sparse",
                         "MethodxNumberVars"=paste("lasso",sum(beta.glmnet!=0),sep=""))
      rocpROC <- pROC::roc(predictor = Ypred.glmnet,
                           response = RespValidationNum,
                           smooth = F, auc = T, levels = c(0, 1), direction = "<")
      temp$AUC <- rocpROC$auc[1]
      temp$NumberSelectedVars <- sum(beta.glmnet!=0)
      dfAUC <- rbind(dfAUC, temp)
    }
    #take length(maxSel) unique values
    ind_unique <- sapply(unique(dfAUC$NumberSelectedVars),function(x)
      rev(which(dfAUC$NumberSelectedVars==x))[1])
    dfAUC <- dfAUC[ind_unique[c(1:10,floor(seq(11,length(ind_unique),length.out=10)))],]
    dfAUC$Codata <- "none"
    toc <- proc.time()[[3]];
    print(toc - tic) #elapsed time

    save(fit.glmnet,dfAUC,file=fname)
  }
}
#> [1] "Summary of group sizes:"
#>     0     1
#> 12324   514
#> [1] "Res1G1.Rdata"
#> [1] "Res2G1.Rdata"
#> [1] "Res3G1.Rdata"
#> [1] "Resg1Lasso.Rdata"
#> [1] "Summary of group sizes:"
#>     0     1
#> 12324   514
#> [1] "Res1G2.Rdata"
#> [1] "Res2G2.Rdata"
```

```
#> [1] "Res3G2.Rdata"
#> [1] "Resg2Lasso.Rdata"
```

Set plotting parameters

First, set some general parameters that we use in plotting the figures:

```
#Plots: general parameters----
wdth<-600
hght<-wdth*5/8
wdthpdf <- wdth/75
hghtpdf <- hght/75
ts <- 16 #basis text size in figures
ls <- 1.5 #basis line size in figures
ps <- 2 #basis point size in figures
sz <- 2 #point size
strk <- 1.5 #stroke size
palette <- "Dark2"
gg_color_hue <- function(n) {
  hues = seq(15, 375, length = n + 1)
  hcl(h = hues, l = 65, c = 100)[1:n]
}
colsAUC <- gg_color_hue(4)
colsAUC <- seq_gradient_pal(low="black",high="white")((0:4)/5)
```

Load presaved results

Then, load the pre-saved results to reproduce the figures:

```
#Load data for plots----
#analyses with other co-data model settings
setting_names <- c("Positive+monotone constraints","GAM_ML",
                   "Positive constraints")
dfAUCall <- data.frame()
dfVk <- list(data.frame(),data.frame(),data.frame())
for(i in 1:length(setting_names)){
  for(g in 1:length(G_all)){
    fname <- paste(pathResults,"Res",i,"G",g,".Rdata",sep="")
    load(fname)
    dfAUC$Setting <- setting_names[i]
    dfAUC$Groups <- G_all[g]
    dfAUCall <- rbind(dfAUCall, dfAUC)
    #lasso (same for settings)
    fname <- paste(pathResults,"Resg",g,"Lasso.Rdata",sep="")
    load(fname)
    dfAUC$Setting <- setting_names[i]
    dfAUC$Groups <- G_all[g]
    dfAUCall <- rbind(dfAUCall, dfAUC)

    indCodata <- attributes(Res$gamma)$codataSource
    #prior variance before truncation for each co-data source
    for(c in 1:length(Z_all[[g]])){
      vk <- as.vector(Res$tauglobal*(Z_all[[g]][[c]]%*%Res$gamma[indCodata==c]))
      if(c==3) vk <- as.vector(Res$tauglobal*(Z_all_rank[[g]][[c]]%*%Res$gamma[indCodata==c]))
```

```
      temp <- data.frame("Variable"=1:p,
                         "Priorvar"=vk,
                         "Priorvar*weight"=vk*Res$w[c])
      temp$Groups <- G_all[g]
      temp$Setting <- setting_names[i]
      temp$Gamma0 <- Res$gamma0
      temp$Tau2 <- Res$tauglobal
      temp$Sigma2 <- Res$sigmahat
      temp$Codata <- names(Z_all[[g]])[c]

      if(c==1){
        temp$values <- factor(CoDataTrain$RoepmanGenes,levels=c(0,1),
                              labels=c("out","in"))
      }else{
        temp$values <- values[[g]][[c]]
      }
      if(i%in%2:3){
        temp$Constraints <- F
      }else{
        temp$Constraints <- T
      }
      dfVk[[c]] <- rbind(dfVk[[c]],temp)
    }
  }
}
for(c in 1:3){
  dfVk[[c]]$Groups <- as.factor(dfVk[[c]]$Groups)
}
dfAUCall$Groups <- factor(dfAUCall$Groups)
dfAUCall$Setting <- factor(dfAUCall$Setting, levels=unique(dfAUCall$Setting)[c(2,3,1)],
                           labels=unique(dfAUCall$Setting)[c(2,3,1)])
dfAUCall$SettingNum <- factor(dfAUCall$Setting, levels=unique(dfAUCall$Setting)[c(2,3,1)],
                              labels=paste("Setting",1:3))
dfAUCall$Method <- as.character(dfAUCall$Method)
dfAUCall$Method[grepl("vars",dfAUCall$MethodxNumberVars)] <- "ecpc+postSelect"
```

Figure co-data source contributions

The contribution of each co-data source for either 20 or 50 splines is plotted with the next code block. The estimated prior variance contribution for the correlation co-data shows large deviations near the boundaries, which increase when 50 instead of 20 splines are used (Setting 1). While p-splines have no boundary effects in regular regression models [2], these effects may be the result from the signal regression nature of the model used in estimating the co-data variable weights. To dampen the boundary effects, it may be beneficial to transform the co-data values such that the values spread out more evenly, e.g. using the empirical cumulative distribution function. Fitting a co-data model with positive (and monotone) constraints (Setting 2 and 3) results in smoother functions than when it is fit without constraints.

```
dfVk2 <- rbind(dfVk[[2]],dfVk[[3]])
dfVk2$SettingNum <- factor(dfVk2$Setting, levels=unique(dfVk2$Setting)[c(2,3,1)],
                           labels=paste("Setting",1:3))

ggplot(dfVk2)+
  aes(x=values,y=Priorvar)+
  geom_line(aes(linetype=Groups),alpha=0.6,size=ls)+
```

```
  facet_wrap(Codata~SettingNum, scales="free", nrow=2)+
  facetted_pos_scales(x = list(NULL,NULL,NULL,
                               NULL,scale_x_log10(),scale_x_log10()))+
  #facet_wrap(Codata~SettingNum, scales="free_x", nrow=2)+
  labs(y="Prior variance", x="Continuous co-data variable")+
  guides(linetype=guide_legend(title="# Splines"))+
  theme_bw()+
  theme(axis.text.x=element_text(size=ts),
        axis.text.y=element_text(size=ts),
        axis.title.x=element_text(size=ts+2),
        axis.title.y=element_text(size=ts+2),
        legend.text=element_text(size=ts),
        legend.title=element_text(size=ts+2),
        legend.key.width = unit(2,"cm"),
        #legend.position = "bottom",
        strip.text=element_text(size=ts))#,
#> Warning: Using `size` aesthetic for lines was deprecated in ggplot2 3.4.0.
#> i Please use `linewidth` instead.
```

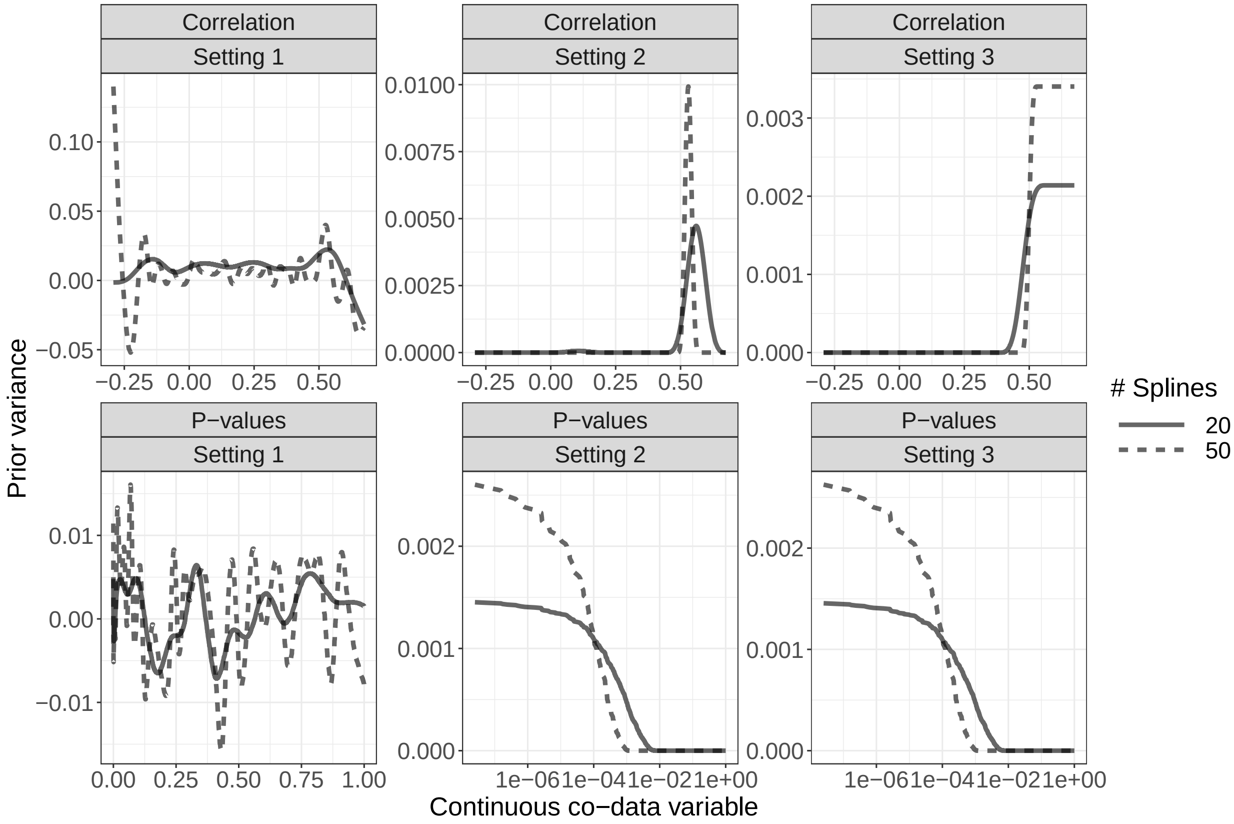

Figure prediction performance

The AUC for different models is plotted with the following code block. While adding the constraints stabilises posterior selection for highly sparse models, it generally does not benefit the prediction performance when compared to the GAM co-data model. The GAM co-data model results in the best prediction performance among sparse models, though, in practice, the simpler lasso model may be preferred as it shows competitive performance. The overall best prediction performance on the test data is retrieved by the full, dense model when the (unconstrained) generalised additive co-data model is used with 50 splines.

```r
#Plot AUC----
lims <- range(dfAUCall$AUC,0.5) #set limits for AUC plots

ggplot(dfAUCall[dfAUCall$Sparsity=="sparse",])+
  aes(x=NumberSelectedVars,y=AUC,col=Method)+
  geom_point(aes(shape=Method),size=ps,stroke=1)+
  geom_line(aes(linetype=Method),size=ls,alpha=0.2)+
  facet_grid(Groups~SettingNum)+
  geom_hline(data=dfAUCall[dfAUCall$Sparsity=="dense",],
             aes(yintercept=AUC,col=Method,linetype=Method), size=ls)+
  geom_point(data=dfAUCall[dfAUCall$Sparsity=="dense",],
             aes(y=AUC,col=Method,shape=Method),x=1, size=0)+
  scale_shape_manual(values=c("ecpc"=32,"ecpc+postSelect"=19,
                              "ecpc+squeezy"=4,
                              "ordinary.ridge"=32,"lasso"=17))+
  scale_linetype_manual(values=c("ecpc"=1,"ecpc+postSelect"=1,
                                 "ecpc+squeezy"=0,
                                 "ordinary.ridge"=5,"lasso"=5))+
  scale_color_manual(values=c("ecpc"=colsAUC[1],"ecpc+postSelect"=colsAUC[1],
                                 "ecpc+squeezy"=colsAUC[1],
                                 "ordinary.ridge"=colsAUC[3],"lasso"=colsAUC[3]))+
  coord_cartesian(ylim=lims)+
  labs(x="# variables")+
  theme_bw()+
  theme(axis.text.x=element_text(size=ts),
        axis.text.y=element_text(size=ts),
        axis.title.x=element_text(size=ts+2),
        axis.title.y=element_text(size=ts+2),
        legend.text=element_text(size=ts),
        legend.title=element_text(size=ts+2),
        legend.key.width = unit(2,"cm"),
        #legend.position = "bottom",
        strip.text=element_text(size=ts))#,
```

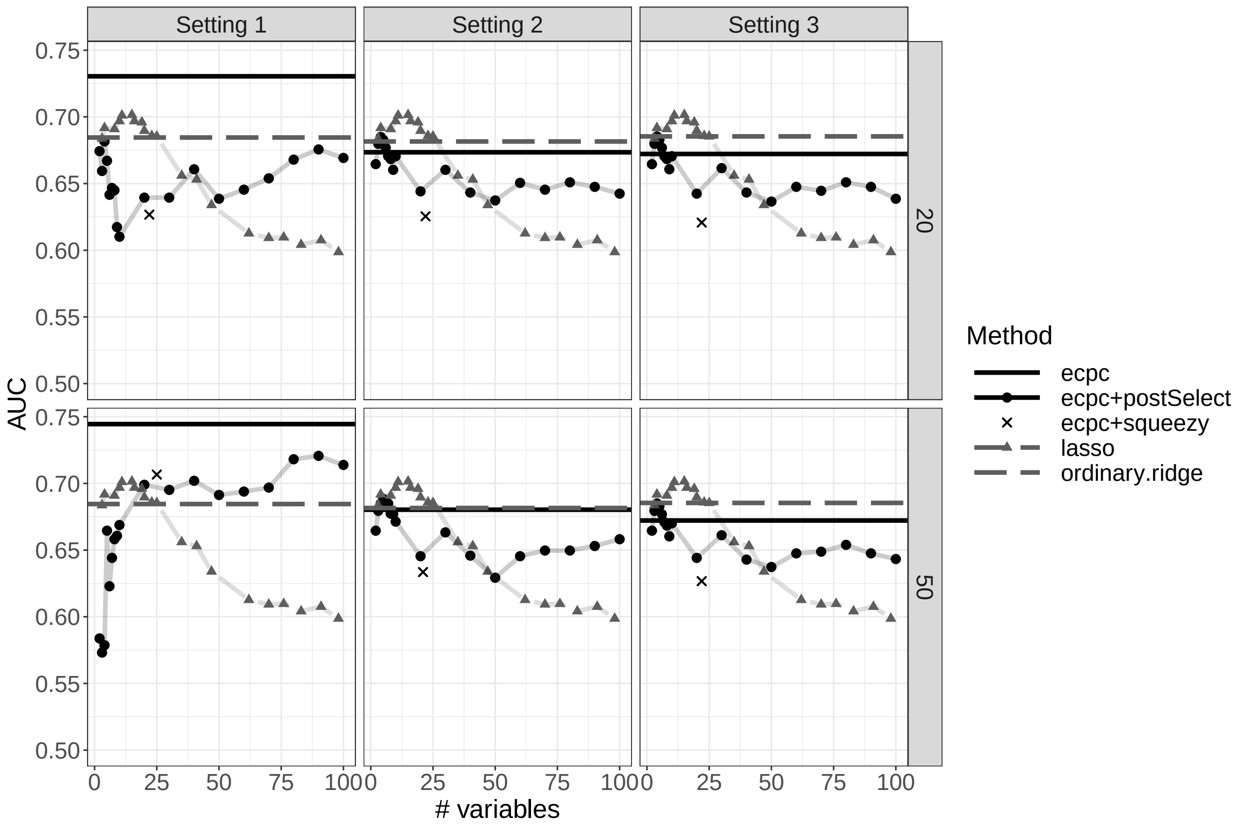

References

[1] van Nee, M.M., Wessels, L.F.A., van de Wiel, M.A.: ecpc: an R-package for generic co-data models for high-dimensional prediction

[2] Eilers, P.H.C., Marx, B.D.: Flexible smoothing with b-splines and penalties. Statistical Science 11(2), 89–102 (1996).
